# Fast and accurate modelling of longitudinal and repeated measures neuroimaging data

**DOI:** 10.1016/j.neuroimage.2014.03.029

**Published:** 2014-07-01

**Authors:** Bryan Guillaume, Xue Hua, Paul M. Thompson, Lourens Waldorp, Thomas E. Nichols

**Affiliations:** a Cyclotron Research Centre, University of Liège, 4000 Liège, Belgium; b Department of Statistics, University of Warwick, Coventry, UK; c Global Imaging Unit, GlaxoSmithKline, Stevenage, UK; d Imaging Genetics Center, Laboratory of Neuro Imaging, Dept. of Neurology & Psychiatry, UCLA School of Medicine, Los Angeles, CA 90095, USA; e Department of Psychological Methods, University of Amsterdam, Amsterdam, The Netherlands; f Warwick Manufacturing Group, University of Warwick, Coventry, UK; g Oxford Centre for Functional MRI of the Brain, University of Oxford, Oxford, UK

**Keywords:** Longitudinal Modelling, Sandwich Estimator, Marginal Modelling, ADNI

## Abstract

Despite the growing importance of longitudinal data in neuroimaging, the standard analysis methods make restrictive or unrealistic assumptions (e.g., assumption of Compound Symmetry—the state of all equal variances and equal correlations—or spatially homogeneous longitudinal correlations). While some new methods have been proposed to more accurately account for such data, these methods are based on iterative algorithms that are slow and failure-prone. In this article, we propose the use of the Sandwich Estimator method which first estimates the parameters of interest with a simple Ordinary Least Square model and second estimates variances/covariances with the “so-called” Sandwich Estimator (SwE) which accounts for the within-subject correlation existing in longitudinal data. Here, we introduce the SwE method in its classic form, and we review and propose several adjustments to improve its behaviour, specifically in small samples. We use intensive Monte Carlo simulations to compare all considered adjustments and isolate the best combination for neuroimaging data. We also compare the SwE method to other popular methods and demonstrate its strengths and weaknesses. Finally, we analyse a highly unbalanced longitudinal dataset from the Alzheimer's Disease Neuroimaging Initiative and demonstrate the flexibility of the SwE method to fit within- and between-subject effects in a single model. Software implementing this SwE method has been made freely available at http://warwick.ac.uk/tenichols/SwE.

## Highlights

• Standard neuroimaging longitudinal methods may lead to invalid results.
• The Sandwich Estimator (SwE) method is proposed as an alternative approach.
• Adjustments to the standard SwE method are reviewed and proposed.
• Monte Carlo simulations are used to isolate a good combination of adjustments.
• The SwE method is shown to be a fast, easy to specify and accurate approach.

## Introduction

Longitudinal data analysis is of increasing importance in neuroimaging, particularly in structural and functional MRI studies. There were over 1000 publications in 2012 to mention “longitudinal fMRI”, which is 3.9% of all “fMRI” 2012 publications and up from 1.5% in 2000.^2^ Unfortunately, while the current versions of the two most widely used packages (i.e. SPM and FSL) are computationally efficient, when they model more than two time points per subject they must make quite restrictive assumptions. In particular, FSL v5.0 must assume Compound Symmetry, a simple covariance structure where the variances and correlations of the repeated measures are constant over time, and a fully balanced design. SPM12 unrealistically assumes a common longitudinal covariance structure for the whole brain. This motivates recent publications proposing methods to better model neuroimaging longitudinal data (Bernal-Rusiel et al., 2013a, Bernal-Rusiel et al., 2013b, Chen et al., 2013, Li et al., 2013, Skup et al., 2012), however, all of these methods entail iterative optimisation at each voxel.

In neuroimaging, the two most widely longitudinal approaches currently used are the Naïve Ordinary Least Squares (N-OLS) modelling and the Summary Statistics Ordinary Least Squares (SS-OLS) modelling. The N-OLS method tries to account for the intra-visit correlations existing in the data by including subject indicator variables (i.e. an intercept per subject) in an OLS model. This approach is fast, but does not allow one to make valid inferences on pure between-subject covariates (e.g., group intercept or gender) and is valid only under a balanced design and Compound Symmetry (CS). The SS-OLS method proceeds by first extracting a summary statistic of interest for each subject (e.g., slope with time) and then uses a group OLS model to infer on the summary measures. This method is also fast and has the advantage of reducing the analysis of correlated data to an analysis of independent data, but this summary data may be highly variable as it is based on single-subject fits. In the context of one-sample t-tests, Mumford and Nichols (2009) showed that this approach is robust under heterogeneity, but warned that it is probably not the case for more general regression models.

In biostatistics, the analysis of longitudinal data is a long-standing problem and is generally performed by using either Linear Mixed Effects (LME) models or marginal models. The LME models include random effects which account for the intra-visit correlations existing in the data. Nevertheless, they require iterative algorithms which are generally slow and may fail to converge to a correct solution. Another issue with LME models is the complexity of specifying and fitting the model. For example, the random effects and the covariance structure of the error terms need to be specified (e.g., only random intercepts? Also random slopes?) and, unfortunately, a misspecification of those may lead to invalid results. These are particularly serious problems in neuroimaging as model assessment is difficult and a single model must be used for the whole brain. As a consequence, the use of LME models in neuroimaging may be prohibitively slow, and may lead to statistical images with missing or invalid results for some voxels in the brain. To limit the convergence issues, one may be tempted to use a LME model with only a random intercept per subject. Unfortunately, like the N-OLS model, this model assumes CS which is probably not realistic, especially for long studies carried out over years and with many visits. In contrast, the marginal modelling approach implicitly accounts for random effects, treats the intra-visit correlations as a nuisance and focuses the modelling only on population averages. They have appealing asymptotic properties, are robust against model misspecification and, as there are no explicit random effects, are easier to specify than LME models. However, they only focus on population-averaged inferences or predictions, typically require iterative algorithms and assume large samples.

Recently, Bernal-Rusiel et al. (2013a) proposed the use of LME models to analyse longitudinal neuroimaging data, but only on a small number of regions of interest or biomarkers, Chen et al. (2013) and Bernal-Rusiel et al. (2013b) extended the use of the LME models to mass-univariate settings. In particular, Bernal-Rusiel et al. (2013b) proposed the use of a spatiotemporal LME method based on a parcellation of the brain into homogeneous areas; in each area, they model the full spatiotemporal covariance structure by assuming a common temporal covariance structure across all the points and a simple spatial covariance structure. Skup et al. (2012) and Li et al. (2013) proposed to use marginal models to analyse neuroimaging longitudinal data. Specifically, Skup et al. (2012) proposed a Multiscale Adaptive Generalised Method of Moments (MA-GMM) approach which combines a spatial regularisation method with a marginal model called Generalised Methods of Moments (GMM; Hansen, 1982, Lai and Small, 2007) and Li et al. (2013) proposed a Multiscale Adaptive Generalised Estimating Equations (MA-GEE) approach which also combines a spatial regularisation method, but with a marginal model called Generalised Estimating Equations (GEE; Liang and Zeger, 1986). Thanks to their appealing theoretical asymptotic properties, the two latter methods seem very promising for analysing longitudinal neuroimaging data. Nevertheless, like the LME models, they require iterative algorithms, which make them slow, and – due to the fact that they rely on asymptotic theoretical results – their use may be problematic in small samples.

In this paper, we propose an alternative marginal approach. We use a simple OLS model for the marginal model (i.e. no subject indicator variables) to create estimates of the parameters of interest. For standard errors of these estimates, we use the so-called Sandwich Estimator (SwE; Eicker, 1963) to account for the repeated measures correlation. The main property of the SwE is that, under weak conditions, it is asymptotically robust against misspecification of the covariance model. In particular, this robustness allows us to combine the SwE with a simple OLS model which has no covariance model. Thus, this method is easy to specify and, with no need for iterative computations, is fast and has no convergence issues. Moreover, the proposed method can deal with unbalanced designs and heterogeneous variances across time and groups (or even subjects; more below on this). In addition, note that the SwE method can also be used for cross-sectional designs where repeated measures exist, such as fMRI studies where multiple contrasts of interests are jointly modelled, or even for family designs where subjects from the same family cannot be assumed independent. Nevertheless, like the MA-GMM and MA-GEE methods, the SwE method relies on asymptotic theoretical results, guaranteeing accurate inference only in large samples. Therefore, we also review and propose small sample adjustments that improve its behaviour in small samples.

The remainder of this paper is organised as follows. Starting from the LME model and its implied marginal model, we introduce the SwE method in its standard form. Then, we review and propose different adjustments to the SwE in order to improve its behaviour, mainly in the case of small samples. Finally, we assess the SwE method with intensive Monte Carlo simulations in a large range of settings and, more particularly, by analysing real brain images acquired as part of the Alzheimer's Disease Neuroimaging Initiative (ADNI; Mueller et al., 2005).

## Methods

### The Linear Mixed Effects model and the marginal model

Using the formulation of Laird and Ware (1982), the LME model for individual *i* is

(1) $$y_{i}=X_{i}\beta +Z_{i}b_{i}+\epsilon_{i}$$

where *y_i_* is a vector of *n_i_* observations for individual *i* = 1,2,…,*m*, *β* is a vector of *p* fixed effects which is linked to *y_i_* by the *n_i_* × *p* design matrix *X_i_*, *b_i_* is a vector of *r* individual random effects which is linked to *y_i_* by the *n_i_* × *r* design matrix *Z_i_*, and *ϵ_i_* is a vector of *n_i_* individual error terms which is normally distributed with mean 0 and covariance Σ*_i_*. The individual random effects *b_i_* are also normally distributed, independently of *ϵ_i_*, with mean 0 and covariance *D*. Typically, the *p* fixed effects might include an intercept per group, a linear effect of time per group, a quadratic effect of time per-group or per-visit measures effects like, in the case of Alzheimer's Disease, the MMSE (Mini-Mental State Examination) score. The *r* random effects usually include a “random intercept” for each subject (modelled by a constant in *Z_i_*) and may also include a “random slope” for each subject.

Instead of posing a model for each subject consisting of (common) fixed and (individual) random components, we can fit a model with only fixed components and let the random components induce structure on the random error. This is the so-called marginal model, which, for subject *i*, has the form

(2) $$y_{i}=X_{i}\beta +\epsilon_{i}^{*}$$

where the individual marginal error terms ϵ_*i*_^∗^ have mean 0 and covariance *V_i_*. Typically, the covariance is taken to be unstructured, but if data arise as per the LME model specified above, then *V*_*i*_ = *Σ*_*i*_ + *Z*_*i*_*DZ*_*i*_. We will denote by *X* the grand design matrix, the *n* × *p* stacked matrix of the *m X_i_*'s, where *n* = ∑ _*i*_ *n*_*i*_ is the total number of observations.

In LME models, the randomness of the data is modelled by both the random effects *b_i_* and the error terms *ϵ_i_*. The random effects *b_i_* have an important impact on the variance modelling and have to be chosen carefully. This makes LME models quite difficult to specify in practice. In contrast, in the marginal model, all the randomness is treated as a nuisance and is modelled by the marginal error terms ϵ_*i*_^∗^. Therefore, the marginal models do not require the specification of random effects, making them easier to specify than LME models. Moreover, the marginal models are more flexible because they only require that the *V_i_* be positive semi-definite. In the LME models, both Σ*_i_* and *D* have to be positive semi-definite which is more restrictive (Molenberghs and Verbeke, 2011, Verbeke and Molenberghs, 2009, West et al., 2006). However, the marginal models are only focused on population-averaged inferences and predictions, and do not offer the possibility to make inferences on random effects or to predict subject-specific profiles like LME models do. Nevertheless, subject-specific inferences or predictions are not generally of interest in longitudinal neuroimaging studies and therefore, a marginal approach should be sufficient to analyse the data (for inferences on random effects parameters, see Lindquist et al., 2012).

In both models, the fixed effects parameters are estimated by

(3) $$\hat{\beta }={\left(\sum_{i=1}^{m}\;X_{i}^{\prime }W_{i}X_{i}\right)}^{-1}\sum_{i=1}^{m}\;X_{i}^{\prime }W_{i}y_{i}$$

where *W_i_* is the so-called working covariance matrix of individual *i* (Diggle et al., 1994, Liang and Zeger, 1986). If *W_i_* = I the identity matrix, it is the Ordinary Least Squares (OLS) estimate. If *W*_*i*_ = *V*_*i*_^− 1^, it is the Generalized Least Squares (GLS) estimate, the Uniform Minimum Variance Unbiased Estimate.

The covariance matrix of the fixed parameter estimates $\mathrm{var}\left\{\hat{\beta }\right\}$ is estimated by

(4) $$S=\underset{\mathrm{Bread}}{\underset{︸}{{\left(\sum_{i=1}^{m}\;X_{i}^{\prime }W_{i}X_{i}\right)}^{-1}}}\underset{\mathrm{Meat}}{\underset{︸}{\left(\sum_{i=1}^{m}\;X_{i}^{\prime }W_{i}{\hat{V}}_{i}W_{i}X_{i}\right)}}\underset{\mathrm{Bread}}{\underset{︸}{{\left(\sum_{i=1}^{m}\;X_{i}^{\prime }W_{i}X_{i}\right)}^{-1}}},$$

where ${\hat{V}}_{i}$ is an estimate of the subject covariance *V_i_*. The central part of this estimate can be conceptualised as a piece of meat between two slices of bread, giving rise to the name of Sandwich Estimator (SwE). If $m^{-1}\sum_{i=1}^{m}\;X_{i}^{\prime }W_{i}{\hat{V}}_{i}W_{i}X_{i}$ consistently^3^ estimates *m*^− 1^ ∑ _*i* = 1_^*m*^*X*_*i*_′*W*_*i*_*V*_*i*_*W*_*i*_*X*_*i*_, the SwE converges asymptotically to the true covariance matrix $\mathrm{var}\left\{\hat{\beta }\right\}$, even if *W_i_* is misspecified (Diggle et al., 1994, Eicker, 1963, Eicker, 1967, Huber, 1967, White, 1980). For GLS with $W_{i}={{\hat{V}}_{i}}^{-1}$, the first two terms of *S* cancel and only the rightmost term remains. For OLS with *W_i_* = *I*, we obtain the simplest version of the SwE which was first introduced by Eicker, 1963, Eicker, 1967. Note that, in practice, other choices for *W_i_* are considered by assuming a non-identity structure for *W_i_* and parametrising it with a vector of parameters, which then has to be estimated (Diggle et al., 1994, Liang and Zeger, 1986). These alternative choices are motivated by the fact that, even if the use of *W_i_* = I yields consistent estimates and has been shown to be almost as efficient as the GLS estimator in some settings (Liang and Zeger, 1986, McDonald, 1993), it may lead to a non-negligible loss of efficiency^4^ that can be ameliorated by more complicated forms of *W_i_* (Fitzmaurice, 1995, Zhao et al., 1992). In particular, Fitzmaurice (1995) shows that, in the context of clustered binary data, an important loss of efficiency may arise for within-cluster covariates when the within-cluster correlation is high. Nevertheless, Pepe and Anderson (1994) showed that using a non-diagonal working covariance matrix may lead to inaccurate estimates of $\hat{\beta }$ and, further, using a non-identity covariance matrix requires generally the use of iterative algorithms to estimate the covariance parameters. Finally, as shown in the subsection Construction of the design matrix below, the loss of efficiency can be limited by an appropriate construction of the design matrix. For all these reasons, in this paper, we only focus on the use of the identity for *W_i_*. See, however, Li et al. (2013) for the use of non-diagonal working covariance matrix within the framework of neuroimaging data, and Pepe and Anderson (1994) on the validity of using such working covariance matrices.

In LME models, the elements of *V_i_* are generally defined as functions of a set of covariance parameters *θ* such that *V_i_* = *V_i_*(*θ*). These covariance parameters *θ* are estimated by either Maximum Likelihood (ML) or Restricted Maximum Likelihood (ReML) and are used to construct an estimate of *V_i_* (Harville, 1977). In the SwE, *V_i_* is usually estimated from the residuals $e_{i}=y_{i}-X_{i}\hat{\beta }$ by

(5) $${\hat{V}}_{i}=e_{i}e_{i}^{\prime }$$

(Diggle et al., 1994). In the literature, the corresponding SwE is often referred to as HC0 (see, e.g., Long and Ervin, 2000) where “HC” stands for “Heteroscedasticity Consistent” and “0” stands for the fact that no small sample adjustment (see subsection Small sample adjustments) is made. Following this numbering, in this paper, we will refer to the corresponding SwE as *S*_0_.

To perform inference on a linear combination of the parameters, H_0_ : *Cβ* = 0, a Wald test is generally used:

(6) $$T={\left(C\hat{\beta }\right)}^{\prime }{\left(\mathrm{CS}C^{\prime }\right)}^{-1}\left(C\hat{\beta }\right)/q$$

where *C* is a matrix (or a vector) defining the combination of the parameters (contrast) tested and *q* is the rank of *C*. In large samples, this Wald test follows a *χ*_*q*_^2^ distribution. In small samples, while the obvious choice is an F-distribution with *q* and *n–p* degrees of freedom, we show in the subsection Small sample adjustments that this is not a good approximation of the true null distribution of *T* when the SwE method is used.

### Construction of the design matrix

In longitudinal data, the covariates have generally a between-subject component and a within-subject component. In the ADNI study, for example, the *Age* covariate has a between-subject component which can be summarised by the subject mean ${\bar{\mathrm{Age}}}_{i}$ and a within-subject component which can be summarised by the difference with the subject mean $\mathrm{Age}-{\bar{\mathrm{Age}}}_{i}$. Including only the *Age* covariate in the design matrix means that we implicitly assume that the effects on the response is the same for both components. Actually, the effects of each component can be very different and, as shown by Neuhaus and Kalbfleisch (1998), the assessment of the effect of such between/within-subject covariates on the response can be very misleading. Therefore, we follow the recommendation of Neuhaus and Kalbfleisch (1998) and systematically split this kind of covariates into between- and within-subject components and include both in the design matrix. Moreover, as shown in Table 1, this helps also to improve the efficiency of the SwE method when assuming an identity working covariance matrix. This result shows that splitting the *Age* covariate makes the SwE nearly as efficient as GLS. It also demonstrates the (well-known) importance of centring covariates when inference is made on the intercepts, as this can be of interest in longitudinal fMRI studies. As the only reason to use a nontrivial working covariance matrix is to improve efficiency, we find that these covariate-splitting results are a compelling reason to only consider an identity working covariance matrix, and hence, in this paper, we exclusively use *W_i_* = I.

**Table 1:** Impact of splitting covariates into separate within- and between-subject covariates. Ages for full 817 subjects ADNI dataset were used to construct 4 models: (1) *Intercept* and *Age*, (2) *Intercept* and centred *Age*, (3) *Intercept*, mean age per subject ${\bar{\mathrm{Age}}}_{i}$, and intra-subject-centred age $\mathrm{Age}-{\bar{\mathrm{Age}}}_{i}$, and (4) *Intercept*, centred mean age per subject ${\bar{\mathrm{Age}}}_{i}-\bar{\mathrm{Age}}$, and intra-subject-centred age $\mathrm{Age}-{\bar{\mathrm{Age}}}_{i}$. The relative efficiency is shown for each model for 3 possible values of *ρ*, the common intra-visit correlation. Here, we define relative efficiency as the ratio between the variance of the GLS estimate and the variance of the SwE estimate.

		Relative efficiency		
Model	Covariate	*ρ* = 0	*ρ* = 0.5	*ρ* = 0.95
1	*Intercept*	1	0.88	0.40
	*Age*	1	0.88	0.40
2	*Intercept*	1	0.94	0.89
	$\mathrm{Age}-\bar{\mathrm{Age}}$	1	0.88	0.40
3	*Intercept*	1	0.92	0.87
	${\bar{\mathrm{Age}}}_{i}$	1	0.92	0.87
	$\mathrm{Age}-{\bar{\mathrm{Age}}}_{i}$	1	1.00	1.00
4	*Intercept*	1	0.94	0.89
	${\bar{\mathrm{Age}}}_{i}-\bar{\mathrm{Age}}$	1	0.92	0.87
	$\mathrm{Age}-{\bar{\mathrm{Age}}}_{i}$	1	1.00	1.00

### Homogeneous SwE

The standard SwE estimates a separate *V_i_* for each subject, based only on the residuals of the *i*-th subject (Eq. (5)). Nevertheless, if the studied population can be subdivided into *n_G_* groups within which the subjects are sharing similar properties, we may assume that the variances and covariances over subjects within each group are actually homogeneous (Pan, 2001). For instance, in the ADNI study, the whole population can be divided into 3 groups: the Normal control (N), Mild Cognitive Impairment (MCI) and Alzheimer's Disease (AD) groups in which the subjects may be assumed to share the same variances and covariances. We argue that this is a reasonable assumption as virtually all standard longitudinal neuroimaging analyses assumes homogeneous variance over all subjects. Therefore, in this paper, we propose an alternative version of the SwE which relies on the assumption of a common covariance matrix *V*_0g_ for all the individuals belonging to group *g* = 1,…,*n_G_*. To estimate *V*_0g_, the observations have to be firstly classified into *k_g_* visit categories (homogeneous groups) consistently defined between subjects in group *g*. For example, in the ADNI study, the MCI subjects were scanned at 0, 6, 12, 18, 24 and 36 months allowing us to divide the observations into *k*_MCI_ = 6 visit categories. Then, defining $m_{\mathrm{gk}k^{\prime }}$ as the number of subjects in group *g* who have data at both visit *k* and *k*′, *e*_*ik*_ as the residual of subject *i* at visit *k* and I(*g*, *k*, *k*′) as the subset of subjects in group *g* who have data at both visit *k* and *k*′, the *k*^*th*^ diagonal element of *V*_0g_ can then be estimated by

(7) $${\left({\hat{V}}_{0g}\right)}_{\mathrm{kk}}=\frac{1}{m_{\mathrm{gkk}}}\sum_{i\in I\left(g,k,k\right)}\;e_{\mathrm{ik}}^{2}.$$

The off-diagonal elements of *V*_0g_ corresponding to the visits *k* and *k*′ can be estimated by

(8) $${\left({\hat{V}}_{0g}\right)}_{kk^{\prime }}={\hat{\rho }}_{0\mathrm{gk}k^{\prime }}\sqrt{{\left({\hat{V}}_{0g}\right)}_{\mathrm{kk}}{\left({\hat{V}}_{0g}\right)}_{k^{\prime }k^{\prime }}}$$

where ${\hat{\rho }}_{0\mathrm{gk}k^{\prime }}$ is an estimate of the correlation at visits *k* and *k*′ in the group *g* and which can be computed by

(9) $${\hat{\rho }}_{0\mathrm{gk}k^{\prime }}=\frac{\sum_{i\in I\left(g,k,k^{\prime }\right)}\;e_{\mathrm{ik}}e_{ik^{\prime }}}{\sqrt{\left(\sum_{i\in I\left(g,k,k^{\prime }\right)}\;e_{\mathrm{ik}}^{2}\right)\left(\sum_{i\in I\left(g,k,k^{\prime }\right)}\;e_{ik^{\prime }}^{2}\right)}}.$$

Note that, due to the possible presence of missing data, ${\hat{V}}_{0g}$ may not be positive semi-definite and, as a consequence, may lead to inaccurate results. Therefore, in presence of missing data, we make a spectral decomposition of ${\hat{V}}_{0g}$ and check whether all the eigenvalues of ${\hat{V}}_{0g}$ are positive. If this is not the case, we set all the negative eigenvalues to zero and reconstruct ${\hat{V}}_{0g}$ with the new eigenvalues, ensuring that ${\hat{V}}_{0g}$ is positive semi-definite. Note also that we normalise with 1/*m*_*gkk*_ instead of the usual bias corrective term 1/(*m*_*gkk*_ − 1) as we consider this sort of bias correction with other small sample adjustments in the next subsection, Small sample adjustments. Thus, in this SwE version, each ${\hat{V}}_{i}$ corresponds to a subset of the corresponding common covariance matrix ${\hat{V}}_{0g}$ depending on the visits measured for subject *i*. If the assumption of a common covariance matrix over subjects in a same group is valid, then the *V*_*i*_ should be more efficiently estimated in comparison to the standard approach. Note that this new SwE version depends on the way the population is subdivided and has two extreme cases, one assuming a single group and the other considering *m* homogeneous groups, equivalent to the standard SwE. We differentiate the various SwE versions using subscripts and superscripts on *S*: superscripts refer to the use of groups, with *S*^*Hom*^ referring to the use of *n*_*G*_ homogeneous groups, and *S*^*Het*^ referring to the standard SwE where heterogeneous, per-subject covariance estimates are used; subscripts refer to different possible small sample adjustments, described in the next subsection.

### Small sample adjustments

In small samples, it is well known that the use of the standard SwE *S*_0_^*Het*^ (heterogeneous, standard SwE, no small sample adjustment) may lead to inaccurate inferences (Chesher and Jewitt, 1987, Long and Ervin, 2000, MacKinnon and White, 1985). There are two explanations for this effect. The first explanation is that, since *S*_0_ uses the Maximum Likelihood Estimate for each *V*_*i*_, it is generally biased and tends to make liberal inferences (i.e. inflated False Positive Rates). The second explanation is that, because the standard Wald test (Eq. (6)) does not account for the randomness in *S*, the sampling distribution of *T* has heavier tails than the usual *χ*_*q*_^2^ distribution. Therefore, a naïve use of *S*_0_^*Het*^ with *T* following a *χ*_*q*_^2^ null distribution also gives liberal inferences. Those two issues have led several authors to propose different adjustments to improve the behaviour of the SwE in small samples.

The first improvements proposed in the literature were focused on the correction of the bias of the SwE. The simplest adjustments proposed consist of multiplying the raw residuals *e*_*ik*_ by a correction factor before using them to estimate *V*_*i*_. There are three principal alternative estimates based on this approach: *S*_1_^*Het*^ (Hinkley, 1977, MacKinnon and White, 1985), *S*_2_^*Het*^ (Horn et al., 1975, MacKinnon and White, 1985) and *S*_3_^*Het*^ (MacKinnon and White, 1985). Note that, in the SwE literature, they are often referred to as HC1, HC2 and HC3, respectively. *S*_1_^*Het*^ consists of using the raw residuals *e*_*ik*_ multiplied by $\sqrt{n/\left(n-p\right)}$ instead of the raw residuals *e*_*ik*_; *S*_2_^*Het*^ consists of using the adjusted residuals *e*_*ik*_/(1 − *h*_*ik*_)^1/2^ (where *h*_*ik*_ is the diagonal element of the Hat matrix *X*(*X*′*X*)^− 1^*X*′ corresponding to the observation of subject *i* at visit *k*) instead of the raw residuals *e*_*ik*_; and *S*_3_^*Het*^ consists of using *e*_*ik*_/(1 − *h*_*ik*_) instead of the raw residuals *e*_*ik*_. Here, we also propose to use these small sample adjustments to compute each ${\hat{V}}_{0g}$ in the homogeneous versions of the SwE.

Subsequently, other authors proposed another type of improvement, altering the null distribution of the Wald test to account for the additional variability of the SwE (Bell and McCaffrey, 2002, Fay and Graubard, 2001, Hardin, 2001, Kauermann and Carroll, 2001, Lipsitz et al., 1999, Mancl and DeRouen, 2001, Pan and Wall, 2002, Waldorp, 2009). Most of the proposed adjustments consist of using a t-distribution (or an F-distribution) instead of a Normal distribution (or a *χ*^2^ distribution) for the statistical test null distribution. The challenge is then to correctly define the degrees of freedom of the distribution. Here, we propose to use an approximate test statistic and null distribution similar to the one proposed in Pan and Wall (2002):

(10) $$\frac{\nu -q+1}{\mathrm{\nu q}}{\left(C\hat{\beta }\right)}^{\prime }{\left(\mathrm{CS}C^{\prime }\right)}^{-1}\left(C\hat{\beta }\right)\sim F\left(q,\nu -q+1\right)$$

where *v* is a degrees of freedom parameter that has to be estimated. The justification of the proposed test and details about the estimation of *v* can be found in Appendix A. The proposed approximate test is valid for both the standard Heterogeneous and modified homogeneous SwE versions, but generally yields different estimates for *v*. As explained in Appendix A, homogeneous versions of the SwE produce more precise estimates of *v* than heterogeneous versions, further motivating the use of the homogeneous SwE. Note that, for a contrast of rank *q* = 1, the test simply becomes

(11) $$\frac{C\hat{\beta }}{\sqrt{\mathrm{CS}C^{\prime }}}\sim t\left(\nu \right).$$

### Monte Carlo simulations

Intensive Monte Carlo simulations were used in R (R Core Team, 2013) to assess the SwE method and compare it to the N-OLS, LME and SS-OLS methods. A variety of realistic settings were considered (detailed below), with 10,000 realisations created for each setting.

#### Simulations I

As a first set of simulations, we considered a selection of balanced and unbalanced designs. We used balanced designs consisting of longitudinal data generated for sample sizes of *m* = 12, 25, 50, 100 or 200 subjects with 3, 5 or 8 visits for each subject (a total of 5 × 3 = 15 distinct sample sizes). The subjects were divided into two groups A and B of equal sizes (except for *m* = 25 where the group A and B had 13 and 12 subjects, respectively) and we considered models consisting of, for each group, an intercept, a linear effect of visit and a quadratic effect of visit using orthogonal polynomials. In addition to these 15 balanced designs, we also considered the unbalanced design corresponding to the real ADNI dataset described in subsection Real data analysis. In order to also assess the methods in an unbalanced design but with a smaller number of subjects, we also considered four subsets of the full ADNI dataset obtained by iteratively removing half of the subjects at random in each group, leading to smaller and smaller sample sizes (*m*_N_ = 229, 114, 57, 29 and 14; *m*_MCI_ = 400, 200, 100, 50 and 25; *m*_AD_ = 188, 94, 47, 24 and 12). For this real unbalanced data design, we considered models consisting of, for each group, an intercept, the centred mean age per subject ${\bar{\mathrm{Age}}}_{i}-\bar{\mathrm{Age}}$ (referred to as cross-sectional “age” effect), the intra-subject centred age $\mathrm{Age}-{\bar{\mathrm{Age}}}_{i}$ (referred to as longitudinal “visit” effect) and their interaction (referred to as “acceleration”).

For each realised dataset, each observation was first generated independently from a standard Normal distribution $N\left(01\right)$. Then, the data for each subject $y_{i}={\left(y_{i1},\ldots ,y_{\mathrm{ik}},\ldots ,y_{in_{i}}\right)}^{⊤}$ was correlated according to one of four different types of intra-visit covariance structure by premultiplying *y_i_* by a square-root factor of the desired covariance matrix. The four covariance structures were generated according to the two following equations:

(12) $$\mathrm{var}\left(y_{\mathrm{ik}}\right)=\alpha_{g}\left(1+\gamma t_{k}\right)$$

(13) $$\mathrm{corr}\left(y_{\mathrm{ik}},y_{ik^{\prime }}\right)=\rho \left(1-\psi |t_{k}-t_{k^{\prime }}|\right),$$

where *α_g_* allows for different variances in each group, *γ* allows the variance to vary with visit, *t*_*k*_ ($t_{k^{\prime }}$, respectively) is the time of measurement at visit *k* (visit *k*′), *ρ* controls the constant correlation over time and *ψ* > 0 allows for a linear decrease of the correlation over time. Table 2 summarises the parameter values used for the four covariance structures in the simulations for both the balanced and unbalanced ADNI designs.

**Table 2:** Covariance parameter values used in the simulations; *γ* and *Ψ* are expressed as “per visit” for the balanced design and “per year” for the ADNI design.

		Covariance parameters							
Design	Covariance structure	*α*_A_	*α*_B_	*α*_N_	*α*_MCI_	*α*_AD_	*γ*	*ρ*	*Ψ*
Balanced	CS	1	1	–	–	–	0	0.95	0
	Toeplitz	1	1	–	–	–	0	1	0.1
	Group heterogeneity	1	2	–	–	–	0	0	0
	Visit heterogeneity	1	1	–	–	–	1	0	0
ADNI	CS	–	–	1	1	1	0	0.95	0
	Toeplitz	–	–	1	1	1	0	1	0.2
	Group heterogeneity	–	–	1	2	3	0	0	0
	Visit heterogeneity	–	–	1	1	1	2	0	0

For null simulations, the data was used immediately after being correlated. For non-null simulations, a signal was added according to the (per-subject centred) effect of visit.

For a given realised dataset and a given design, each of the four estimation methods were used in turn. Using custom R functions, eight versions of the SwE were used: *S*_0_^*Het*^, *S*_1_^*Het*^, *S*_2_^*Het*^, *S*_3_^*Het*^, *S*_0_^*Hom*^, *S*_1_^*Hom*^, *S*_2_^*Hom*^ and *S*_3_^*Hom*^ where the homogeneous groups were defined as groups A and B for the balanced designs and Normal, MCI and AD groups for the real unbalanced designs (see subsections Homogeneous SwE and Small sample adjustments, Homogeneous SwE and Small sample adjustments for descriptions about these SwE versions); the SwE design matrices included all the effects described at the beginning of this subsection; the Wald tests were performed according to Eq. (10) estimating *v* in two different ways: as proposed in Eq. (A.16) and also, naïvely, by *m* − *p*_*B*_ where *p*_*B*_ is the number of pure between-subject covariates (having a constant value for each subject) included in the model (e.g., intercepts, cross-sectional age effect) leading to 16 different variants for the SwE approach. The N-OLS included per-subject dummy variables, and thus precluded the use of the age effect (as age is a linear combination of the dummy variables). The SS-OLS approach used per-subject models, with a design matrix extracted from the appropriate rows and columns of the SwE design matrices, and contrasts that extracted quantities equivalent to the contrasts of interest used with the other models; the final model used with the SS-OLS approach was always a one-measure-per-subject OLS model allowing to test group effects equivalent to the one tested with the other methods. For both the N-OLS and SS-OLS methods, the function lm of the stats R package was used to estimate the model parameters, their variances/covariances and the degrees of freedom used in the Wald tests (i.e. the number of observations minus the number of parameters present in the considered model). The functions lme from the R package nlme (Pinheiro et al., 2013) and lmer from the R package lme4 (Bates et al., 2012) were used to fit the LME models with the SwE design matrices for the fixed effects and a random intercept per subject as random effect; note that, as suggested by one of the reviewers, richer LME models were assessed in a second set of simulations (see subsection Simulations II). As the lme4 package did not propose any estimation for the degrees of freedom, we used the ones estimated by the nlme package (Pinheiro and Bates, 2000) for all the nlme and lme4 Wald tests.

For each realisation and contrast, several Wald tests *T* were computed and compared to F-distributions at a nominal level of significance of 5%. For null dataset, each significant realisation was counted as a False Positive detection and was used to compute the expected False Positive Rates (FPRs) for each method. The FPR of a valid test does not exceed the nominal level, while an invalid or liberal test will have an FPR in excess of the nominal level. Using a Normal approximation to binomial counts over 10,000 realisations, an exact test (FPR = 5%) should have a FPR between (4.57%, 5.43%) with 95% probability. Non-null simulations allowed the estimation of power with the True Positive Rates (TPRs) for each method.

#### Simulations II

Following the suggestions of the reviewers, we performed an additional three sets of simulations. In this set simulations similar to the first set were used, but we also considered LME models with a random intercept and time effect (slope), and LME models with a random intercept, linear and quadratic time effects. For these simulations, the ADNI design and its subsets were considered with a residual error covariance structure consisting of a Toeplitz correlation and an increasing variance over time obtained from the Eqs. (12), (13) with parameters α_N_ = 1, α_MCI_ = 1, α_AD_ = 1, *γ* = 2/year, *ρ* = 1 and *ψ* = 0.2/year. The SwE, N-OLS and SS-OLS methods were fitted as in the first set of simulations. The LME models were fitted using the lme4 package in the same way as the first set of simulations, but, in addition, we used more advanced functions to determine the degrees of freedom for each Wald test. Specifically, we used the vcovAdj and get_ddf_Lb functions of the pbkrtest R package (Halekoh and Højsgaard, 2013) to compute the Kenward-Roger covariance matrix correction and the Kenward–Roger effective degrees of freedom (Kenward and Roger, 1997), respectively.

#### Simulations III

The third set of simulations focused on the power analysis of all the methods (so, also including the two richer LME models investigated in subsection Simulations II) under CS and Toeplitz covariance structures in the unbalanced ADNI design. The covariance structures were produced with the same parameters as in the first set of simulations (see Table 2).

#### Simulations IV

In this final set, we conducted an experiment recording the failure rates of the LME models. For this, we used the same settings as in the first set of simulations (see subsection Simulations I), but only recorded the number of times the functions lme and lmer did not converge to a solution. The LME models considered were the same as the ones investigated in the second set of simulations (see subsection Simulations II), but, in addition, we included a model with a random intercept and a Toeplitz covariance structure for the error terms. Note that the latter model was only fitted with the nlme package as the lme4 package do not allow the specification of correlation structure for the error terms.

### Box's test of Compound Symmetry

As mentioned in the Introduction section, CS is the key assumption that justifies the use of N-OLS or random-intercept LME models. To assess whether the assumption of CS holds, Box (1950) proposed a test based on the determinant of the covariance matrix. It does not, however, accommodate missing data. In the presence of missing data, we construct a CS test using the largest possible subset of the full dataset which has no missingness. Eqs. (7), (8), (9) (assuming only one group) are used to produce, at each voxel, an estimate of the common covariance matrix which can then be tested through the Box's test of CS, producing an image of F-scores (or p-values). Next, this image can be thresholded using a multiple testing correction (e.g., False Discovery Rate) and, if any voxels survive the threshold, we can conclude that there is evidence of violation of the assumption of CS.

### Real data analysis

Data used in the preparation of this article were obtained from the Alzheimer's Disease Neuroimaging Initiative (ADNI) database (adni.loni.usc.edu). The ADNI was launched in 2003 by the National Institute on Aging (NIA), the National Institute of Biomedical Imaging and Bioengineering (NIBIB), the Food and Drug Administration (FDA), private pharmaceutical companies and non-profit organisations, as a $60 million, 5-year public-private partnership. The primary goal of ADNI has been to test whether serial magnetic resonance imaging (MRI), positron emission tomography (PET), other biological markers, and clinical and neuropsychological assessment can be combined to measure the progression of mild cognitive impairment (MCI) and early Alzheimer's disease (AD). Determination of sensitive and specific markers of very early AD progression is intended to aid researchers and clinicians to develop new treatments and monitor their effectiveness, as well as lessen the time and cost of clinical trials.

The Principal Investigator of this initiative is Michael W. Weiner, MD, VA Medical Center and University of California – San Francisco. ADNI is the result of efforts of many co-investigators from a broad range of academic institutions and private corporations, and subjects have been recruited from over 50 sites across the U.S. and Canada. The initial goal of ADNI was to recruit 800 subjects but ADNI has been followed by ADNI-GO and ADNI-2. To date these three protocols have recruited over 1500 adults, ages 55 to 90, to participate in the research, consisting of cognitively normal older individuals, people with early or late MCI, and people with early AD. The follow up duration of each group is specified in the protocols for ADNI-1, ADNI-2 and ADNI-GO. Subjects originally recruited for ADNI-1 and ADNI-GO had the option to be followed in ADNI-2. For up-to-date information, see www.adni-info.org.

The dataset analysed in this paper is a modified version of the dataset produced and detailed by Hua et al. (2013). In brief, the dataset in Hua et al. (2013) consisted on 3314 images obtained after applying Tensor Based Morphometry (TBM) on 3314 brain MRI scans from 229 healthy elderly Normal controls (age: 76.0 ± 5.0 years, 119 Male (M)/110 Female (F)), 400 individuals with amnestic MCI (age: 74.8 ± 7.4 years, 257 M/143 F), and 188 probable AD patients (age at screening: 75.4 ± 7.5 years, 99 M/89 F). As shown in Table 3, the subjects were scanned at screening and followed up at 6, 12, 18 (MCI only), 24, and 36 months (Normal and MCI only) with visits counts of 4.16 ± 1.21, 4.43 ± 1.61 and 3.14 ± 1.07 for the Normal, MCI and AD subjects, respectively. More precisely, 817 screening TBM images were produced by considering the 817 screening scans and a Minimal Deformation Target (MDT) image, obtained from the scans of 40 randomly selected Normal subjects, as baseline; 2497 longitudinal TBM images were produced by considering, for each subject, the follow-up scans and the corresponding screening scan as baseline. More details about this dataset can be found in Hua et al. (2013). The 2497 longitudinal TBM images measure change *relative* to each subject's screening and *not* absolute volume (relative to a template). Therefore, we modified them by multiplying them with their corresponding TBM screening image in order to produce 2497 TBM images reflecting the brain volumes relative to a common baseline, the MDT image. We considered these modified 2497 TBM images with the unchanged 817 screening TBM images as the dataset to be analysed.

**Table 3:** Numbers of subjects scanned at baseline (0 month) and follow-up (6, 12, 18, 24 and 36 months) for the Normal controls (N), Mild Cognitive Impairment (MCI) and Alzheimer's Disease (AD) subjects in the ADNI dataset.

Scanning time	N	MCI	AD	Total
0 month	229	400	188	817
6 months	208	346	159	713
12 months	196	326	138	660
18 months	-	286	-	286
24 months	172	244	105	521
36 months	147	170	-	317

The modified dataset was analysed by using the N-OLS, SS-OLS and SwE methods with the same design matrices as used in the simulations (see subsection Monte Carlo simulations). SPM8 was used for the N-OLS and SS-OLS methods and a homemade SPM8 plug-in was used for the SwE method.

## Results

### SwE versions comparison in very small samples

**Fig. 1:**
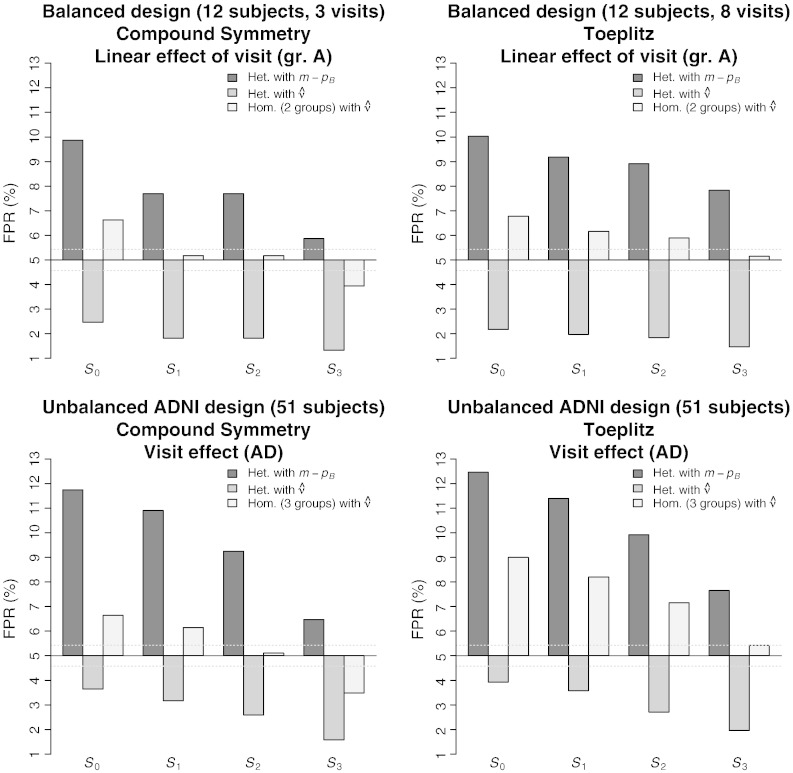
FPR with different versions of the SwE in small samples with Compound Symmetry (*ρ* = 0.95) and Toeplitz (*ψ* = 0.1 per visit in the balanced design and *ψ* = 0.2 per year in the ADNI design) for the balanced and unbalanced ADNI design; all results are based on an F-test at nominal level 5%; *S*_0_, *S*_1_, *S*_2_ and *S*_3_ correspond to the SwE using the raw residuals *e_ik_*, the adjusted residuals $e_{\mathrm{ik}}\sqrt{N/\left(N-p\right)}$, the adjusted residuals *e*_*ik*_/(1 − *h*_*ik*_)^1/2^ and the adjusted residuals *e*_*ik*_/(1 − *h*_*ik*_), respectively; “Het. with *m* − *p_B_*”, “Het. with $\hat{\nu }$” and “Hom with $\hat{\nu }$” correspond to the standard heterogeneous SwE using (naïvely) *m* − *p_B_* as degrees of freedom, the standard heterogeneous SwE using the estimate proposed in Eq. (A.16) as degrees of freedom and the modified homogeneous SwE using the estimate proposed in Eq. (A.16) as degrees of freedom, respectively.

Here, and for all results, we summarise the immense volume of Monte Carlo simulations by selecting the subset of findings that conveys the typical behaviour exhibited by the methods. Exhaustive results can be found in the Web Supplementary Material. Fig. 1 shows typical results obtained for 12 variants of the SwE in very small sample settings for a balanced design (12 subjects) and the unbalanced ADNI design (51 subjects). The standard *S*^*Het*^ tends to be liberal with the use of a naïve estimation of *v* by *m* − *p*_*B*_ (Fig. 1, dark grey bar) and conservative with the estimation of *v* by the estimate proposed in Eq. (A.16) (Fig. 1, medium grey bar). The homogeneous version (assuming homogeneity within groups) controls the FPR more accurately than the heterogeneous versions, with the better results obtained with the versions *S*_2_^*Hom*^ and *S*_3_^*Hom*^ (Fig. 1, light grey bar). Note that the settings selected in Fig. 1 were chosen in order to show some of the most severe adverse behaviour of these two versions, meaning that, they were, in general, controlling the FPR better in the simulations. Overall, *S*_2_^*Hom*^ appears to be slightly liberal and *S*_3_^*Hom*^ slightly conservative.

### Methods comparison

#### FPR control

**Fig. 2:**
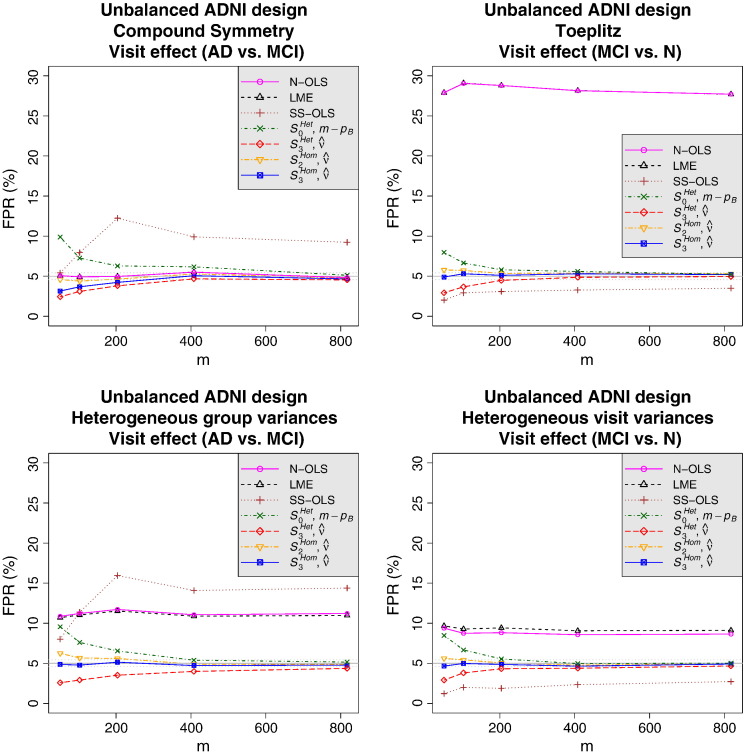
FPR comparison on the visit effect with Compound Symmetry (top left, *ρ* = 0.95), Toeplitz (top right, *ψ* = 0.2 per year), heterogeneous group variances (bottom left, α_N_ = 1, α_MCI_ = 2 and α_AD_ = 3) and heterogeneous visit variances (bottom right, *γ* = 2 per year) for the ADNI design. All results are based on an F-test at nominal level 5%, and the results for the LME method correspond to the random-intercept LME model. See Fig. 1 for a description of the SwE versions.

For the random-intercept LME models, we only show the results obtained with the lme4 package as the results obtained with the nlme package were almost identical. Table 4 summarises qualitatively how the methods were able to control the FPR in the first set of simulations with different settings. The N-OLS method cannot provide inference on between-subject effects, but otherwise shows a performance similar to the random-intercept LME method. Specifically, the N-OLS and random-intercept LME methods struggle with variance heterogeneity (between groups or over time) and Toeplitz covariance structures, being either conservative or liberal depending on the setting. On between-subject effects, the random-intercept LME method has problems with variance heterogeneity. The SS-OLS method fares somewhat better than the N-OLS and random-intercept LME methods for balanced designs, but falls down on variance heterogeneity between groups and within-subject effects in the unbalanced design. Finally, with enough subjects, the SwE (*S*_3_^*Hom*^) seems accurate in all the settings, but, as shown in Fig. 1, Fig. 2, it may slightly suffer from conservativeness in very small samples. Note that, as suggested by one of the reviewers, we also simulated a SwE assuming a common covariance matrix for all the subjects (one group in the modified homogeneous SwE) and found, under heterogeneous group variances, similar poor behaviours as the three other methods. See Web Supplementary Material for additional quantitative results comparing the methods.

**Table 4:** Summary of simulation results for the False Positive Rate (FPR) control in different covariance settings, for between- and within-subject effects, and in the balanced and unbalanced ADNI designs. “R.-int.” stands for Random-intercept; “n/a” stands for not applicable indicating that this type of inference is not possible for that particular method; “●” stands for an accurate FPR control, “+” for an invalid (liberal) FPR control, “−” for a conservative FPR control, “+/−” for both behaviours; “++/−” indicates an FPR control that is generally invalid, but also sometimes conservative; and “●/−” stands for an FPR control sometimes slightly conservative in small sample settings (*m* < 50 in the balanced design and *m* < 200 in the unbalanced ADNI design) but accurate otherwise. See Web Supplementary Material for detailed quantitative results.

Design	Cov. type	Effect type	N-OLS	R.-int. LME	SS-OLS	SwE (*S*_3_^*Hom*^)
Balanced	CS	Between	n/a	●	●	●/−
		Within	●	●	●	●/−
	Toeplitz	Between	n/a	●	●	●/−
		Within	++/−	++/−	●	●/−
	Het. groups	Between	n/a	+/−	+/−	●/−
		Within	+/−	+/−	+/−	●/−
	Het. visits	Between	n/a	●/−	●	●/−
		Within	●	●	●	●/−
Unbalanced (ADNI)	CS	Between	n/a	●	●	●
		Within	●	●	+/−	●/−
	Toeplitz	Between	n/a	●	●	●
		Within	+	+	+/−	●
	Het. groups	Between	n/a	+/−	+/−	●
		Within	+/−	+/−	+/−	●
	Het. visits	Between	n/a	−	●	●
		Within	+	+	+/−	●

Regarding the second set of simulations, the N-OLS, SS-OLS, random-intercept LME and SwE methods exhibited similar behaviours to the ones observed in the first set of simulations under variance heterogeneity over time. The LME models with a random intercept and a random effect of time per subject, and the LME models with a random intercept, a random effect of time and a random quadratic effect of time per subject had similar results to the ones of the SwE (*S*_3_^*Hom*^) method and seemed accurate for all the settings. Note that in the third set of simulations, only the SwE (*S*_3_^*Hom*^) seemed to be able to control the FPR accurately under a Toeplitz covariance structure. In particular, as it can be seen in the Web Supplementary Material, the 2 richer LME models seemed liberal (e.g., in the full ADNI design and testing for a difference of visit effect between AD and MCI subjects at 5% level of significance, the LME model with a random intercept and a random effect of time per subject had a FPR of 6.1% while the LME model with a random intercept, a random effect of time and a random quadratic effect of time per subject had a FPR of 7.4 %).

#### Power analysis

Power comparisons are only interpretable when the methods considered control the FPR. Thus, as the majority of the compared methods had issues to control the FPR under Toeplitz covariance or variance heterogeneity (between groups, over time), we only show power comparisons for CS. Note that a comparison in the Toeplitz case can be found in the Web Supplementary Material.

**Fig. 3:**
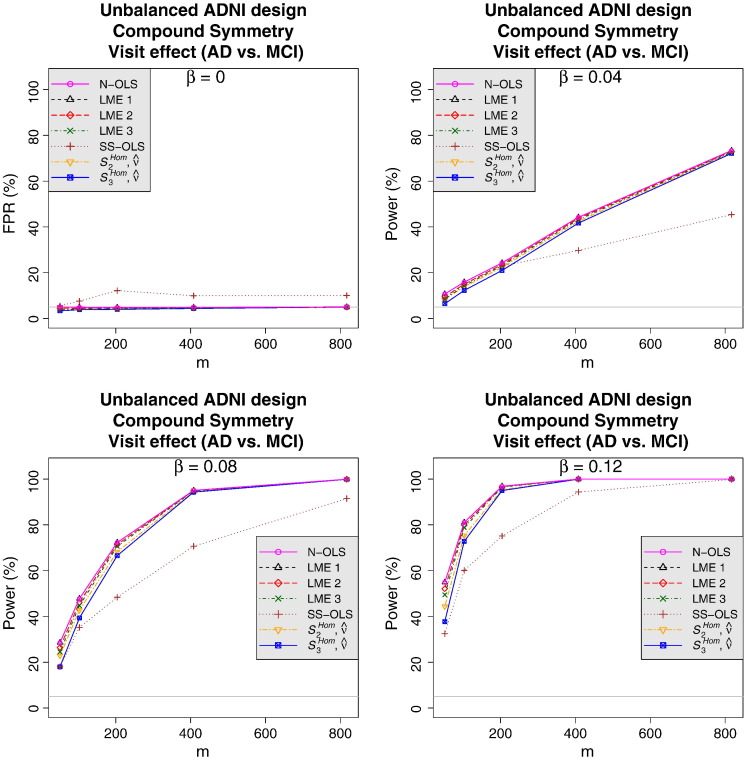
Power with Compound Symmetry (*ρ* = 0.95) for the unbalanced ADNI design, for varying effect sizes. The tested effect is the difference in the visit effect between AD and MCI groups. All results are based on an F-test at nominal level 5%. LME 1, LME 2 and LME 3 correspond to the LME model including a random intercept per subject, the LME model with a random intercept and a random effect of time per subject and the LME model with a random intercept, a random effect of time and a quadratic effect of time per subject, respectively. See Fig. 1 for a description of the SwE versions.

Fig. 3 shows the results of the power analysis for a greater visit effect in AD relative to MCI subjects under the assumption of CS obtained from the third set of simulations (see subsection Simulations III). The SwE method is less powerful than the N-OLS and LME methods with a difference of power larger in very small samples, but becoming narrower and narrower when the sample size increases. Finally, even if the SS-OLS method is liberal for the FPR control (see Fig. 3, top left), it seems clearly less powerful than the SwE approach. This effect may seem counterintuitive, but is explained by noting that the SS-OLS method tends to be less efficient than the SwE method (i.e. that the true variance of the parameters obtained with the SS-OLS method tends to be higher than the true variance of the parameters obtained with the SwE). This can be confirmed by computing the Monte Carlo estimates of the true variances of the SS-OLS and SwE methods and comparing them. For the particular setting of Fig. 3 considering the full ADNI design, it appears that the true variance is 2.9 times bigger for the SS-OLS method than the SwE method. As a consequence, the power of the SS-OLS method will increase more slowly than the one of the SwE method when the effect size increases. Thus, provided that the effect size is large enough to overcome the invalid additional power due to the liberal behaviour of the SS-OLS method, the SwE method will be more powerful than the SS-OLS method, as observed in Fig. 3.

While these results highlight the principal weakness of the SwE method, i.e. a reduced power at low *m*, we stress that these results are only for CS with no variance heterogeneity. When CS or variance homogeneity cannot be safely assumed, only the SwE or LME (with appropriate random effects or covariance structure for the error terms) methods can provide valid inferences.

Note that a power analysis for a greater visit effect in AD relative to MCI subjects under the assumption of a Toeplitz covariance structure can be found in the Web Supplementary Material. For this case, only the SwE (*S*_3_^*Hom*^) seemed to be able to control accurately the FPR, with the N-OLS and random-intercept LME methods appearing highly liberal and the SS-OLS and the two richer LME methods appearing slightly liberal, making them invalid. An interesting observation about these results is that the SS-OLS method seemed to be slightly more or equally as powerful as the SwE method, contradicting the observation made in the CS case (see Fig. 3). Comparing the Monte Carlo estimates of the true variances of each method showed that the SS-OLS method is 1.2 times larger than the SwE method, indicating that the SS-OLS method should be less powerful than the SwE method, like in the CS case. Nevertheless, in this setting, the SS-OLS method actually underestimates the variance and in turn inflates the test statistic to such a degree that the SS-OLS is slightly more powerful.

### LME convergence failure rates

Regarding the convergence failure experiment (see, subsection Simulations IV), the lmer function did not exhibit any convergence failures. However, the lme function exhibited a high rate of convergence problems in many designs. The detailed results about the convergence failures can be found in the Web Supplementary Material.

### Real ADNI analysis

Prior to the analysis of the real ADNI data, we conducted a Box's test of Compound Symmetry as described in subsection Box's test of Compound Symmetry with a reduced dataset of 483 subjects who were all scanned at screening and followed up at 6, 12 and 24 months. After controlling for a False Discovery Rate of 5% (using a Bonferroni correction at level 5%, respectively), 97% (56%, respectively) of the voxels survived the thresholding indicating a strong evidence of non-CS in the data.

**Fig. 4:**
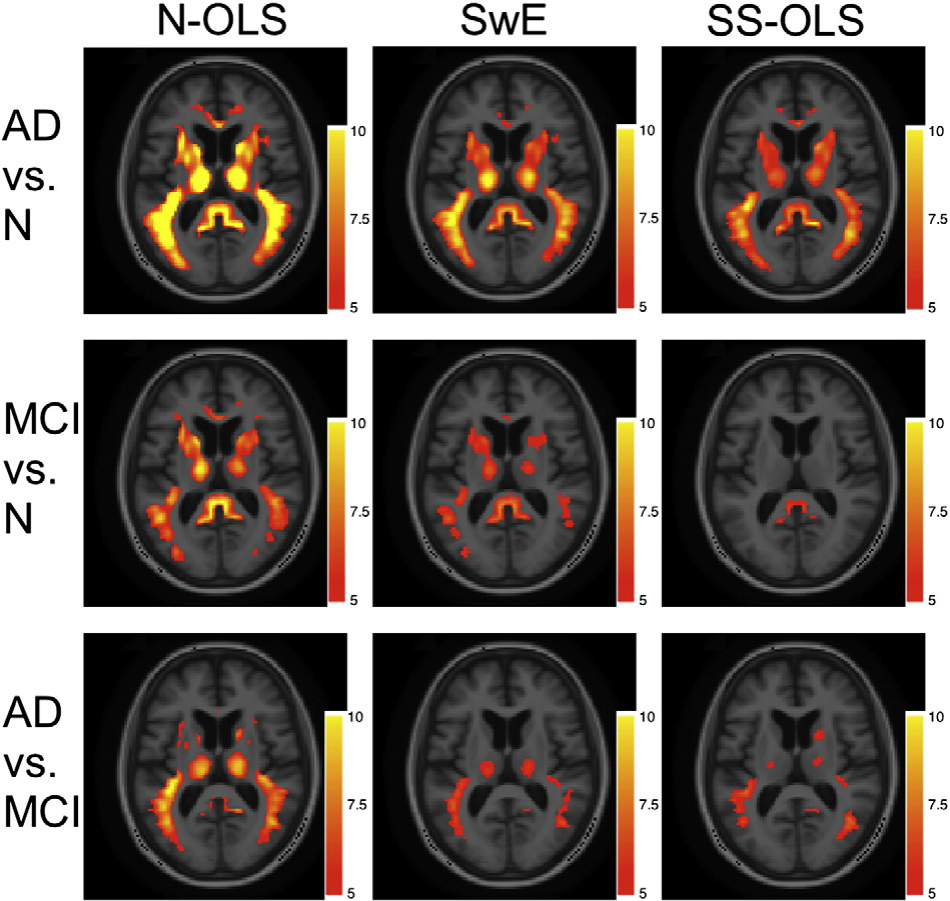
Thresholded one-sided *t* images for the differential visit effect, greater decline in volume in AD relative to N, MCI relative to N and AD relative to MCI, for the N-OLS, SwE (*S*_3_^*Hom*^) and SS-OLS methods; threshold of 5 used for all methods; axial section shown at *z* = 14 mm. Apparent superior sensitivity of the N-OLS method (left) is likely due to inflated significance and poor FPR control; see text and Fig. 2.

Fig. 4 compares the t-score images obtained by the N-OLS, SwE (*S*_3_^*Hom*^) and SS-OLS methods with the real images for contrasts on the difference between groups in terms of visit effect on the brain atrophy (all methods thresholded at 5 for comparison). The N-OLS method has larger t-values and more supra-threshold voxels than the SwE method. While this could be attributed to power differences, with 817 subjects, we expect negligible differences in power. Hence a more likely explanation is the presence of a complex (non-CS) longitudinal covariance structure that results in inflated significance (Fig. 2, top left and bottom). The SS-OLS has smaller t-values and fewer supra-threshold voxels than the SwE method, likely attributable to conservativeness (Fig. 2, right) and/or reduced power (Fig. 3, top left and bottom).

**Fig. 5:**
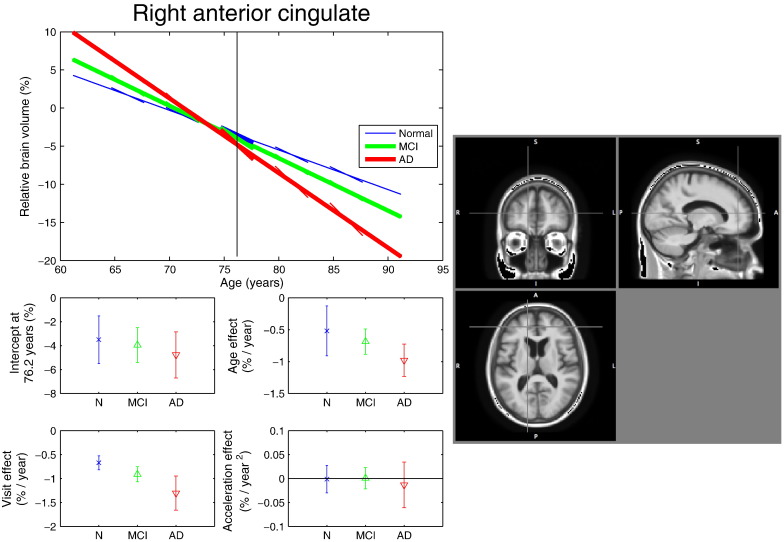
Model fit in the right anterior cingulate cortex. Top plot: linear regression fit obtained with the SwE method (*S*_3_^*Hom*^) at voxel (*x*, *y*, *z*) = (16, 45, 14) mm; the vertical line at 76.2 years marks the average age of the study participants; the thickness of the lines reflects the strength of the t-scores obtained for the age effect (the three main lines), the visit effect (the three secondary lines centred at 76.2 years) and the acceleration effect (the secondary lines centred at 66.2, 71.2, 81.2 and 86.2 years). Bottom plots: 95% confidence intervals for all the parameters of the linear regression. Right image: location of the selected voxel. The confidence intervals suggest that the rate of brain atrophy seems similar for each group and is similar for both the age and the visit effect, indicating consistent cross-sectional and longitudinal volume changes.

**Fig. 6:**
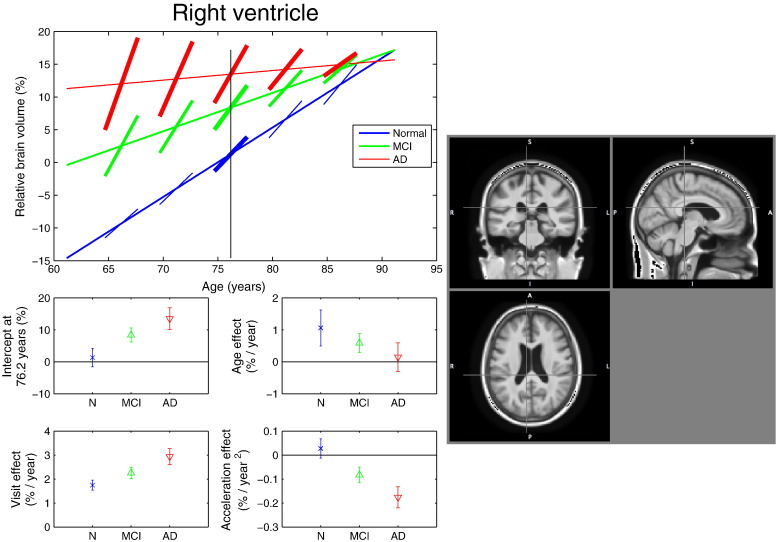
Model fit in the right ventricle. Top plot: Linear regression fit obtained with the SwE method (*S*_3_^*Hom*^) for voxel (*x*, *y*, *z*) = (8 − 35, 24) mm. (See Fig. 5 caption for a description of the different figure components). In the AD and MCI groups a mismatch is observed between cross-sectional and longitudinal effects of time, with a reduced rate of change with increasing age; see body text for more discussion.

**Fig. 7:**
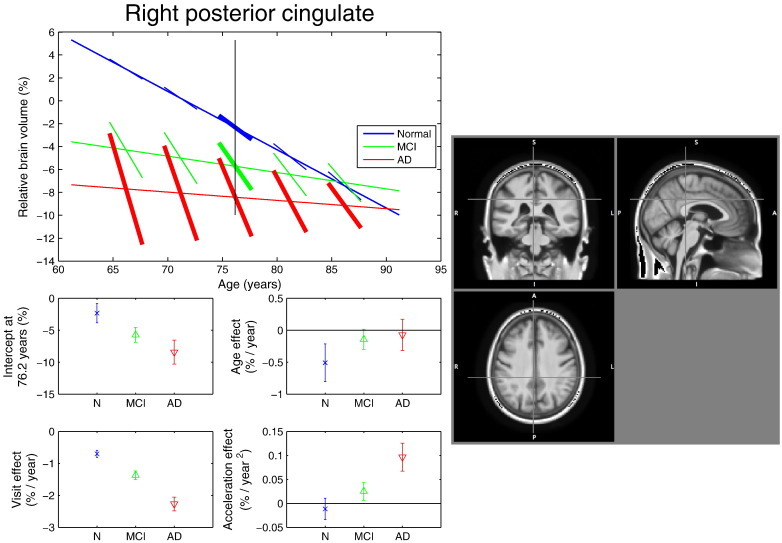
Model fit in the right posterior cingulate. Top plot: Linear regression fit obtained with the SwE method (*S*_3_^*Hom*^) for voxel (*x*, *y*, *z*) = (4, − 39,38) mm. (See Fig. 5 caption for a description of the different figure components). In the AD and MCI groups, there is a mismatch between cross-sectional and longitudinal effects of time, with a reduced rate of change with increasing age; see body text for more discussion.

Fig. 5, Fig. 6, Fig. 7 shows the regression fits for three particular voxels situated in different areas of the brain. Note that these voxels were not selected based on maximal difference between the SwE and N-OLS (or SS-OLS) methods, but rather based on relatively high significance in term of age, visit or acceleration effects in all of the methods (qualitatively, the statistic maps for the three methods are similar). As a reminder from subsection Real data analysis, all the scans represent the relative difference in brain volume from the MDT reference image, as such, a value of 10% in the plots indicates that the brain volume is 10% bigger than in the MDT image. Fig. 5 shows results for a voxel in the right anterior cingulate where there is strong evidence of brain atrophy with age and also with the visit effect. The rate of brain atrophy seems similar for each group and is similar for both the age and the visit effect, indicating consistent cross-sectional and longitudinal volume changes. Fig. 6 shows a voxel in the right ventricle where there is strong evidence of an expansion in volume. As expected, this is greater in AD subjects than in MCI or Normal subjects. Fig. 7 shows a voxel in the right posterior cingulate where we observe strong brain atrophy for the AD subjects compared to the Normal subjects. In Fig. 5, Fig. 6, Fig. 7, the Normal subjects have similar intra- and inter-subject effects of time (visit and age effects, respectively), and we generally observe this throughout the brain. In contrast, in the AD and MCI groups, there are inconsistent longitudinal and cross-sectional effects of time. Specifically, there is evidence of a “deceleration”, where the oldest patients exhibit reduced rates of expansion (or contraction) relative to younger patients. One interpretation is a “saturation” effect, where, with advancing disease progress, there is less gray matter left to atrophy and less space in the cranial vault for the ventricles to expand. However, as the ADNI only follows subjects for at most 3 years, an alternative interpretation must be considered. Specifically, instead of this deceleration reflecting an aspect of the disease process, it rather reflects age-dependent heterogeneity in the ADNI cohort. For example, MCI subjects in their 80’s are likely to have systematic differences from the MCI subjects in their 60's, as the former group have survived to their 8th decade free of severe dementia, while some of the latter group will convert to AD in the next 20 years. As pointed out by one of the reviewers, this kind of explanation has already been reported in Thompson et al. (2011).

### Computation time

As suggested by one of the reviewers, we compared the elapsed computation times of the SwE and LME methods obtained on a 2.7 GHz quad-core Intel Core i7 MacBook Pro with 16 GB of memory. For this, we considered the scenario where we would like to analyse the 336,331 in-mask voxels of the ADNI dataset (see subsection Real data analysis) with the two methods in R and test for the presence of a visit effect (AD vs. N subjects). Table 5 shows the results obtained with the SwE version *S*_3_^*Hom*^ (SwE in the table), the LME model including a random intercept per subject (LME 1 in the table), the LME model with a random intercept and a random effect of time per subject (LME 2 in the table), and the LME model with a random intercept, a random effect of time and a quadratic effect of time per subject (LME 3 in the table). Note that our home built R implementation of the SwE method uses four different functions. The first one computes voxel-independent variables which need to be computed only once for the whole brain; the second one computes voxel-specific estimates of *β*, Var(*β*) and other variables needed for the estimation of *v*; the third one computes contrast-specific and voxel-independent variables needed for the estimation of *v*; and the fourth one computes contrast- and voxel-specific estimates of *v*. For the LME models, the (voxel-specific) lmer, (voxel-specific) vcovAdj and (contrast- and voxel-specific) get_ddf_Lb functions were used for each voxel.

**Table 5:** Estimated computation times in days, hours, minutes and seconds in the scenario where the 336,331 in-mask voxels of the TBM ADNI dataset would be tested for an effect of visit (AD vs. N subjects) in R. The setting used corresponded to the one of the second set of simulations (see subsection Simulations II). “n/a”, “ind.” and “spec.” stands for not applicable, independent and specific, respectively; “KR voxel specific” corresponds to the use of the function vcovAdj; see text for additional detail.

Computation level	LME 1	LME 2	LME 3	SwE
Voxel-ind.	n/a	n/a	n/a	0d 0 h 0′ 3″
Voxel-spec.	0d 7 h 41′ 57″	1d 1 h 55′ 22″	9d 6 h 5′ 50″	0d 0 h 7′ 11″
KR voxel-spec.	66d 13 h 28′ 44″	111d 20 h 56′ 57″	213d 23 h 46′ 28″	n/a
Contrast-spec. and voxel-ind.	n/a	n/a	n/a	0d 0 h 0′ 1″
Contrast- and voxel-spec.	71d 8 h 57′ 9″	112d 13 h 45′ 56″	215d 21 h 38′ 46″	0d 0 h 0′ 30″
Total	138d 6 h 7′ 50″	225d 12 h 38′ 15″	439d 3 h 31′ 4″	0d 0 h 7′ 44

## Discussion

While the SwE is an ubiquitous biostatistical tool, to our knowledge, we are the first authors to provide a detailed study of its small sample properties in a range of settings important for neuroimaging and identify a *non-iterative* estimator that works well for the analysis of longitudinal neuroimaging data.

We have shown that the SwE method is a flexible computationally efficient alternative to the N-OLS, SS-OLS and LME methods. When the simplest covariance structure, CS, cannot be assumed, the SwE (*S*_3_^*Hom*^) method and the LME method using appropriate random effects to model correctly the true covariance structure were the only methods that consistently controlled the FPR. In particular, the SS-OLS method was not able to control the FPR in the ADNI design. This effect can be explained by the fact that an inhomogeneity in the distribution of the summary statistics is likely to occur when subjects do not have the same number of observations, leading to a lack of control of the FPR as observed in our simulations. We also have shown that the N-OLS, SS-OLS and LME methods may be inaccurate when there exists heterogeneity in group variance. Nevertheless, it is worth noting that all of these methods can be adapted to accommodate such a heterogeneity by, for example, specifying different variances for each group in their model. In the SwE method, the use of a marginal model simplifies the specification of the predictors and the interpretation of parameters. In particular, both within- and between-subject covariates can be used, and we have illustrated the ease with which cross-sectional and longitudinal time effects can be used. In particular, testing the interaction of these two time effects revealed a “deceleration” effect in the MCI and AD patient groups that was missing from the healthy controls. We have noted, however, the importance of replacing an arbitrary covariate with two, one purely within-subject and one purely between-subject.

We note that, with our focus on structural data, we did not investigate one-sample t-tests on subject summary statistics. While one-sample t-tests have been shown to be robust under heterogeneity (Mumford and Nichols, 2009), these methods are however less flexible than other regression methods which allow for the inclusion of covariates. Another approach not investigated in this manuscript and which is implemented in SPM12, first estimates a common covariance matrix structure for the whole brain and assumes it to be the true covariance structure for all the voxels in the brain. While there are likely voxels where this common covariance structure is valid, in order to safely use this approach, tests for the accuracy of the assumed covariance should be examined.

In this manuscript, we have also made a comparison between the computation times needed by the SwE method (see subsection Computation time) compared to the LME method, demonstrating the computational efficiency of the SwE method. Nevertheless, it is worth noting that the R implementation of the LME method does not make use of any voxel-independent pre-computations as we used for the SwE method, and thus the LME method could potentially be accelerated. Also, the computation time of the Kenward–Roger covariance matrix correction and the Kenward–Roger effective degrees of freedom were surprisingly high, indicating a likely inefficient implementation in the pbkrtest R package. This seems to indicate that the computation time of the LME models could be reduced. Nevertheless, we doubt that this reduction would be large enough to match the computational efficiency of the SwE method.

We have discussed the use of the Box's test for CS, and found ample evidence that the ADNI data's covariance structure is inconsistent with CS.

The principal limitation of the SwE method regards power. When CS holds, it has slightly inferior power to the LME and N-OLS methods, and the recommended *S*_3_^*Hom*^ SwE was sometimes slightly conservative for samples smaller than 50 in a balanced design and 200 in the highly unbalanced ADNI design. However, when CS doesn't hold, or when there is variance heterogeneity, the N-OLS, SS-OLS and random-intercept LME fail to control False Positives and are *unusable*. Thus, this conservativeness seems like a reasonable price to pay for validity. Also, even when CS holds, it may be desirable to use the SwE method over the N-OLS method to allow fitting of a mix of within- and between-subject covariates.

If more power is needed, one can use some form of spatial regularisation or more complicated models like in Skup et al. (2012), Bernal-Rusiel et al. (2013b) or Li et al. (2013). Nevertheless, while those methods are expected to be more powerful, they require iterative algorithms, which makes them slower than the SwE method. Moreover, there is no evidence that, at least in some settings, they will do this with a good control of the FPR. Notably, Zhang (2008) showed that using a spatial regularisation will tend to decrease the variance of the estimates (which will tend to increase the power), but also increase their bias (which will tend to alter the accuracy).

It would be desirable to use permutation methods (see, e.g., Nichols and Holmes, 2002) in combination with the SwE to produce non-parametric inferences. However, permutation tests assume that the scans are exchangeable under the null hypothesis, incompatible with longitudinal or repeated measures data. Bootstrap methods (see, e.g., Efron and Tibshirani, 1994), in contrast, do not require the exchangeability assumption and may be applicable. As there are different types of bootstrap tests to consider and extensive small-sample simulations needed to validate this asymptotic method, we have left this for future study.

As another future direction, we intend to check the validity of the Random Field Theory (see, e.g., Worsley et al., 1996) with the SwE method. It is indeed not guaranteed that the assumptions required by the Random Field Theory hold when the SwE method is used. As such, at present, we can only recommend the use of a False Discovery Rate control in order to deal with the multiple comparison problem.

While the present work was motivated and illustrated on a longitudinal dataset, we stress that the SwE can be used to analyse other types of correlated data encountered in neuroimaging. For example, it can be used to analyse cross-sectional fMRI studies where multiple contrasts of interests are jointly modelled or cross-sectional family studies where subjects from the same family cannot be assumed independent.

Finally, for the real data analysis, the N-OLS, SS-OLS and SwE methods show clearly different results with the SwE method finding fewer significant voxels than the N-OLS method, but more than the SS-OLS method. This seems to be in accordance with our non-CS simulations in which the N-OLS method poorly controls the FPR (and thus has inflated significance; Fig. 2) and the SS-OLS method which is less powerful than the SwE method (Fig. 3). In the simulations, the SwE was accurate for all the different type of covariance structure tested and this seems to make the SwE one of the most trustworthy methods for the analysis of the ADNI data. An SPM extension implementing the SwE method has been made available for use from the authors (http://warwick.ac.uk/tenichols/SwE).

## Appendix A Approximate test with the SwE

Let *U*_1_, *U*_2_, …, *U*_*l*_ be a sequence of *p*-dimensional random variables, each independently distributed as a Wishart distribution *W*_*p*_(*ν*_*i*_, *Σ*_*i*_/*ν*_*i*_). Nel and Van der Merwe (1986) showed that

(A.1) $$\sum_{i=1}^{l}\;U_{i}\;\dot{\sim }\;W_{p}\left(\nu ,\Sigma /\nu \right),$$

where $\dot{\sim }$ stands for “is approximately distributed as”, *Σ* = ∑ _*i* = 1_^*l*^ *Σ*_*i*_ and,

(A.2) $$\nu =\frac{\mathrm{tr}\left(\Sigma^{2}\right)+{\left(\mathrm{tr}\left(\Sigma \right)\right)}^{2}}{\sum_{i=1}^{l}\;\frac{\mathrm{tr}\left(\Sigma_{i}^{2}\right)+{\left(\mathrm{tr}\left(\Sigma_{i}\right)\right)}^{2}}{\nu_{i}}}.$$

where tr is the trace operator. We will refer to this result as the NVDM approximation.

In the context of a homogeneous version of the SwE with *n_G_* groups, if there is no missing data, *V*_0g_ is estimated by

(A.3) $${\hat{V}}_{0g}=\frac{1}{m_{g}}\sum_{i\in I\left(g\right)}\;e_{i}^{*}e_{i}^{{*}^{\prime }}$$

where I(*g*) is the subset of subjects belonging to group *g* and *e*_*i*_^∗^ is an adjusted version of the residuals of subject *i*. If each *e*_*i*_^∗^ is correctly adjusted in such a way that each covariance matrix var(*e*_*i*_^∗^) is equal to the covariance matrix of its corresponding true error term var(ϵ_*i*_), then they can be assumed to follow a Normal distribution with mean 0 and variance *V*_0g_ for all *i* ∈  I(*g*). Then, for all *i* ∈  I(*g*), we would have

(A.4) $$B_{i}=\frac{1}{m_{g}}e_{i}^{*}e_{i}^{{*}^{\prime }}\sim W_{k_{g}}\left(1,V_{0g}/m_{g}\right)$$

by the definition of a Wishart distribution (Härdle and Simar, 2007), where *k_g_* is the size of *e*_*i*_^∗^. If the different subjects' residuals *e*_*i*_^∗^ were independent, we would have

(A.5) $${\hat{V}}_{0g}=\sum_{i\in I\left(g\right)}\;B_{i}\sim W_{k_{g}}\left(m_{g},V_{0g}/m_{g}\right),$$

by the additive property of Wishart distributions. However, this is not the case due to covariates shared between subjects. To account for this dependence, let us first consider a n × *p* design matrix *X* that is separable into *n*_X_ sub-design matrices *X_u_* of size *n*_u_ × *p* such that, defining *A_u_* as the set of non-zero columns in *X_u_*, the collection of sets {*A_u_*: u = 1,…, *n*_X_} is pairwise disjoint. Further, let *X* be composed of *p_B_* pure between-subject covariates (e.g., group intercept, cross-sectional effect of age) and *p_W_* pure within-subject (e.g., longitudinal effect of visit) as recommended in subsection Construction of the design matrix. In such a situation, the residuals *e*_*i*_^∗^ can be considered to be in a space of dimension *m*_*i*_ − *p*_*Bi*_ where *m_i_* is the number of subjects included in the sub-design matrix containing subject *i* and *p_Bi_* is the number of pure between-subject covariates in this sub-design matrix that are not all-zero. Now, we treat the *B_i_*'s as independent random variables following a Wishart distribution $W_{k_{g}}\left(\nu_{i},V_{0g}/\left(m_{g}\nu_{i}\right)\right)$ with effective degrees of freedom *v_i_* that are estimated by 1 − *p*_*Bi*_/*m*_*i*_. Then, using the NVDM approximation, we get

(A.6) $${\hat{V}}_{0g}\;\dot{\sim }\;W_{k_{g}}\left(\nu_{g},V_{0g}/\nu_{g}\right)$$

where

(A.7) $$\nu_{g}=\frac{m_{g}^{2}}{\sum_{i\in I\left(g\right)}\;\frac{1}{\nu_{i}}}.$$

Now, let us consider the test H_0_ : *Cβ* = 0 where *C* is a matrix (or a vector) of rank *q* defining a combination of parameters (contrast). Contrasting the SwE *S* with *C*, we have

(A.8) $$\mathrm{CS}C^{\prime }=C{\left(\sum_{i=1}^{m}\;X_{i}^{\prime }X_{i}\right)}^{-1}\left(\sum_{g=1}^{n_{G}}\;\sum_{i\in I\left(g\right)}\;X_{i}^{\prime }{\hat{V}}_{0g}X_{i}\right){\left(\sum_{i=1}^{m}\;X_{i}^{\prime }X_{i}\right)}^{-1}C^{\prime }$$

(A.9) $$=\sum_{g=1}^{n_{G}}\;\left(C{\left(\sum_{i=1}^{m}\;X_{i}^{\prime }X_{i}\right)}^{-1}\left(\sum_{i\in I\left(g\right)}\;X_{i}^{\prime }{\hat{V}}_{0g}X_{i}\right){\left(\sum_{i=1}^{m}\;X_{i}^{\prime }X_{i}\right)}^{-1}C^{\prime }\right)$$

(A.10) =∑g=1nGCSC′g

where (*CSC*′)_*g*_ is the contribution of group *g* to the contrasted SwE *CSC*′ and which can be rewritten as

(A.11) $${\left(\mathrm{CS}C^{\prime }\right)}_{g}=\sum_{i\in I\left(g\right)}\;D_{i}{\hat{V}}_{0g}D_{i}^{\prime }$$

where

(A.12) $$D_{i}=C{\left(\sum_{j=1}^{m}\;X_{j}^{\prime }X_{j}\right)}^{-1}X_{i}^{\prime }.$$

Then, for all *i* ∈ I(*g*), we get

(A.13) $$D_{i}{\hat{V}}_{0g}D_{i}^{\prime }\;\dot{\sim }\;W_{q}\left(\nu_{g},D_{i}V_{0g}D_{i}^{\prime }/\nu_{g}\right)$$

where *q* is the rank of *C*. As each component $D_{i}{\hat{V}}_{0g}D_{i}^{\prime }$ is obtained with the same estimate ${\hat{V}}_{0g}$, there is no contribution of additional degrees of freedom and thus

(A.14) $${\left(\mathrm{CS}C^{\prime }\right)}_{g}\;\dot{\sim }\;W_{q}\left(\nu_{g},\sum_{i\in I\left(g\right)}\;D_{i}V_{0g}D_{i}^{\prime }/\nu_{g}\right).$$

Assuming that the contributions (*CSC*′)_*g*_'s are independent, using the NVDM approximation and noting that $\sum_{g=1}^{n_{G}}\;\sum_{i\in I\left(g\right)}\;D_{i}V_{0g}D_{i}^{\prime }=C\mathrm{var}\left(\hat{\beta }\right)C^{\prime },$ we get

(A.15) $$\mathrm{CS}C^{\prime }=\sum_{g=1}^{n_{G}}\;{\left(\mathrm{CS}C^{\prime }\right)}_{g}\;\dot{\sim }\;W_{q}\left(\nu ,C\mathrm{var}\left(\hat{\beta }\right)C^{\prime }/\nu \right).$$

where

(A.16) $$\nu =\frac{\mathrm{tr}\left({\left(C\mathrm{var}\left(\hat{\beta }\right)C^{\prime }\right)}^{2}\right)+{\left(\mathrm{tr}\left(C\mathrm{var}\left(\hat{\beta }\right)C^{\prime }\right)\right)}^{2}}{\sum_{g=1}^{n_{G}}\;\frac{\mathrm{tr}\left({\left(\sum_{i\in I\left(g\right)}\;D_{i}V_{0g}D_{i}^{\prime }\right)}^{2}\right)+{\left(\mathrm{tr}\left(\sum_{i\in I\left(g\right)}\;D_{i}V_{0g}D_{i}^{\prime }\right)\right)}^{2}}{\nu_{g}}}.$$

Noting that $C\hat{\beta }/\sqrt{\nu }\sim N\left(0,C\mathrm{var}\left(\hat{\beta }\right)C^{\prime }/\nu \right)$ and assuming that $C\hat{\beta }$ and *CSC*′ are independent, we obtain

(A.17) $$\nu {\left(C\hat{\beta }/\sqrt{\nu }\right)}^{\prime }{\left(\mathrm{CS}C^{\prime }\right)}^{-1}\left(C\hat{\beta }/\sqrt{\nu }\right)\;\dot{\sim }\;\frac{\mathrm{\nu q}}{\nu -q+1}F\left(q,\nu -q+1\right)$$

and we finally get the test statistic

(A.18) $$\frac{\nu -q+1}{\mathrm{\nu q}}{\left(C\hat{\beta }\right)}^{\prime }{\left(\mathrm{CS}C^{\prime }\right)}^{-1}\left(C\hat{\beta }\right)\;\dot{\sim }\;F\left(q,\nu -q+1\right).$$

The extension to the heterogeneous SwE case is straightforward as it is equivalent to the homogeneous SwE considering *m* groups composed by a single subject. In practice, var$\left(\hat{\beta }\right)$ and *V*_0g_'s are unknown, thus, their estimates *S* and ${\hat{V}}_{0g}$'s are used instead in Eq. (A.16) to get an estimation of *v*. When a group has a very small number of subjects, it will produce poor variance estimates and, consequently, affects the quality of the estimation of *v*. This motivates our idea of assuming homogeneous variances between subjects in the computation of the SwE.

## Appendix B Supplementary data

Supplementary material Web-based appendix with additional results.

## Footnotes

2 Based on Pubmed searches of “longitudinal AND fMRI” in all fields, versus just “fMRI”. This is a crude measure, but does reflect the growing role of this type of study.

3 An estimator of a parameter is said to be consistent if it converges in probability to the true value of the parameter. Here, this is the case if $\mathrm{li}m_{m\to \infty }m^{-1}\sum_{i=1}^{m}\;X_{i}^{\prime }W_{i}\left({\hat{V}}_{i}-V_{i}\right)W_{i}X_{i}=0$.

4 The efficiency of a scalar estimator is the inverse of estimator variance.
