# Supplementary material for: Fast and accurate modelling of longitudinal and repeated measures neuroimaging data
Source: Neuroimage. 2014 Jul 1;94:287–302. doi: 10.1016/j.neuroimage.2014.03.029 (PMC4073654; doi:10.1016/j.neuroimage.2014.03.029)
Supplement: Supplementary material — Web-based appendix with additional results. [file mmc1.pdf]

Web-based Supplementary Materials for Fast and Accurate Modelling of Longitudinal and Repeated Measures Neuroimaging Data

Bryan Guillaume[a,b,c], Xue Hua[d], Paul M. Thompson[d], Lourens Waldorp[e], Thomas E. Nichols[b,f], the Alzheimer's Disease Neuroimaging Initiative

[a] *Cyclotron Research Centre, University of Liège, 4000 Liège, Belgium*
[b] *Department of Statistics, University of Warwick, Coventry, United Kingdom*
[c] *Global Imaging Unit, GlaxoSmithKline, Stevenage, United Kingdom*
[d] *Imaging Genetics Center, Laboratory of Neuro Imaging, Dept. of Neurology & Psychiatry, UCLA School of Medicine, Los Angeles, CA 90095, USA*
[e] *Department of Psychological Methods, University of Amsterdam, Amsterdam, The Netherlands*
[f] *Warwick Manufacturing Group, University of Warwick, Coventry, United Kingdom*

Supplementary Appendix A: Monte Carlo simulations I results

In this Supplementary Appendix, we report on the detailed evaluations of the False Positive Rate (FPR) obtained with the Monte Carlo simulations described in Subsection 2.5.1 of the main paper. These results, which were used to assess the Sandwich Estimator (SwE) method and compare it to the naïve Ordinary Least Squares (N-OLS), the Summary Statistics Ordinary Least Squares (SS-OLS) and the random-intercept Linear Mixed Effects (LME) methods, are a crucial aspect of this work since the SwE method is an asymptotic approach and we seek to establish the best method to use in small samples.

To enhance the clarity in each Supplementary Figure, we only shows the results obtained for four versions of the SwE method as compared to the random-intercept LME, the N-OLS and the SS-OLS methods. The four SwE versions were chosen according to their performance to control the FPR by selecting the two worst and the two best combinations of adjustments corresponding to S_0^{Het} using $m - p_B$ (the number of subject minus the number of pure between covariates in the model) as degrees of freedom, S_3^{Het} using the estimate proposed in Eq. (A.16) of the main paper as degrees of freedom, S_2^{Hom} using the estimate proposed in Eq. (A.16) of the main paper as degrees of freedom and S_3^{Hom} using the estimate proposed in Eq. (A.16) of the main paper as degrees of freedom, respectively. In order to ease the

browsing through the large amount of Supplementary Figures, Tables 1 and 2 can be used to find the number of the Supplementary Figure associated to a particular setting. A general discussion of this results can be found in Subsections 3.1 and 3.2.1 of the main paper.

Balanced Design

		Group tested		
Effect tested	Design type	A	B	B vs. A
Mean effect	3 visits	1	4	7
	5 visits	2	5	8
	8 visits	3	6	9
Linear effect of visits	3 visits	10	13	16
	5 visits	11	14	17
	8 visits	12	15	18
Quadratic effect of visits	3 visits	19	22	25
	5 visits	20	23	26
	8 visits	21	24	27

Table 1: Supplementary Figure numbers correspondence for the balanced design.

Unbalanced ADNI Design

	Group tested					
Effect tested	N	MCI	AD	MCI vs. N	AD vs. N	AD vs. MCI
Mean effect	28	29	30	31	32	33
Age effect	34	35	36	37	38	39
Visit effect	40	41	42	43	44	45
Acceleration effect	46	47	48	49	50	51

Table 2: Supplementary Figure numbers correspondence for the unbalanced ADNI design.

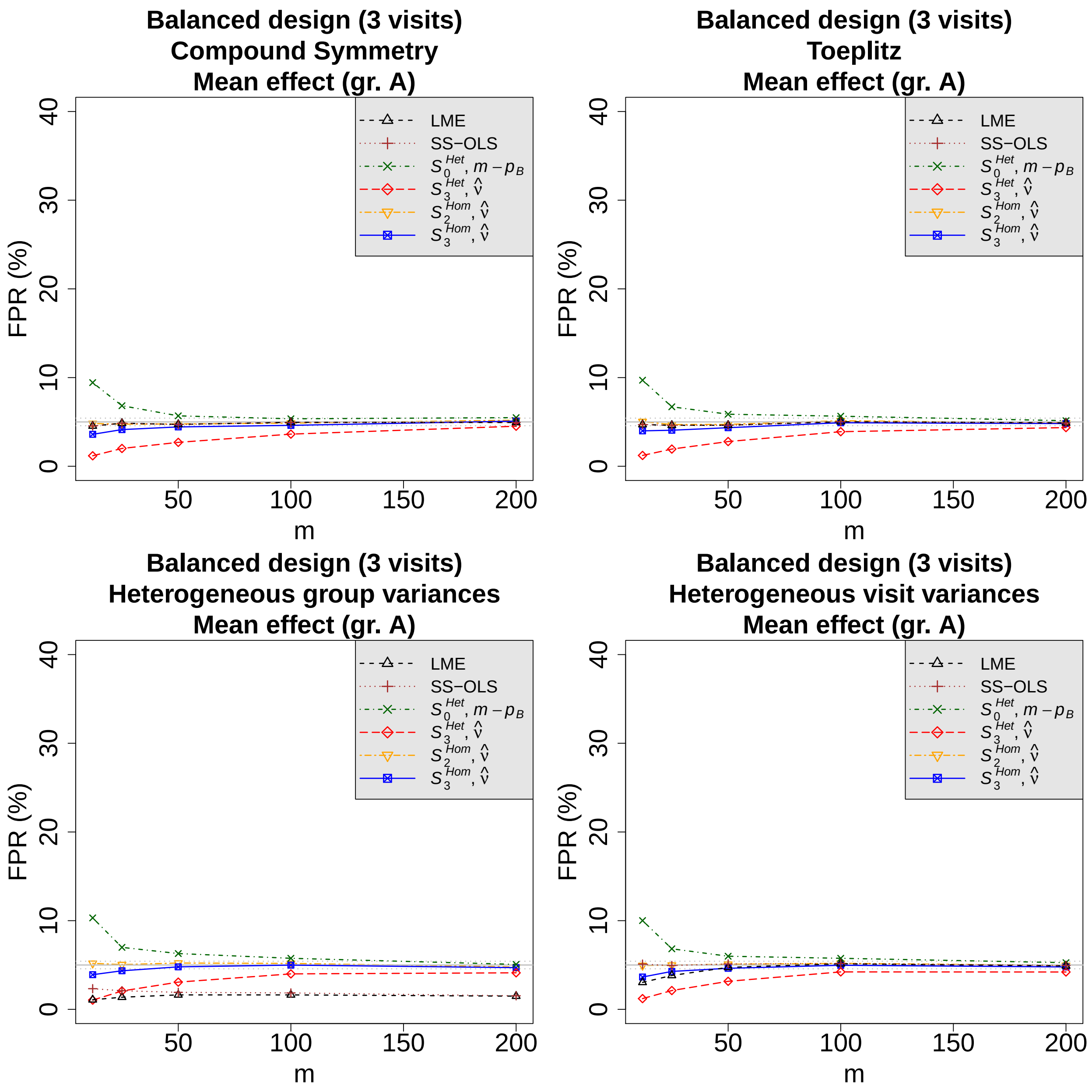

Supplementary Figure 1: FPR comparison on the mean effect of group A with Compound Symmetry (top left, $\rho = 0.95$), Toeplitz (top right, $\psi = 0.1$ per visit), heterogeneous group variances (bottom left, $\alpha_{\text{A}} = 1$ and $\alpha_{\text{B}} = 2$) and heterogeneous visit variances (bottom right, $\gamma = 1$ per visit) for the balanced design with 3 visits per subject; all results are based on an F-test at nominal level 5%; "S_0^{Het}, $m - p_B$" corresponds to the standard heterogeneous SwE using the raw residuals e_{ik} and $m - p_B$ as degrees of freedom; "S_3^{Het}, $\hat{\nu}$" corresponds to the standard heterogeneous SwE using the adjusted residuals $e_{ik}/(1 - h_{ik})$ and the estimate proposed in Eq. (A.16) as degrees of freedom; "S_2^{Hom}, $\hat{\nu}$" corresponds to the modified homogeneous SwE using the adjusted residuals $e_{ik}/(1 - h_{ik})^{1/2}$ and the estimate proposed in Eq. (A.16) as degrees of freedom; S_3^{Hom}, $\hat{\nu}$ corresponds to the modified homogeneous SwE using the adjusted residuals $e_{ik}/(1 - h_{ik})$ and the estimate proposed in Eq. (A.16) as degrees of freedom.

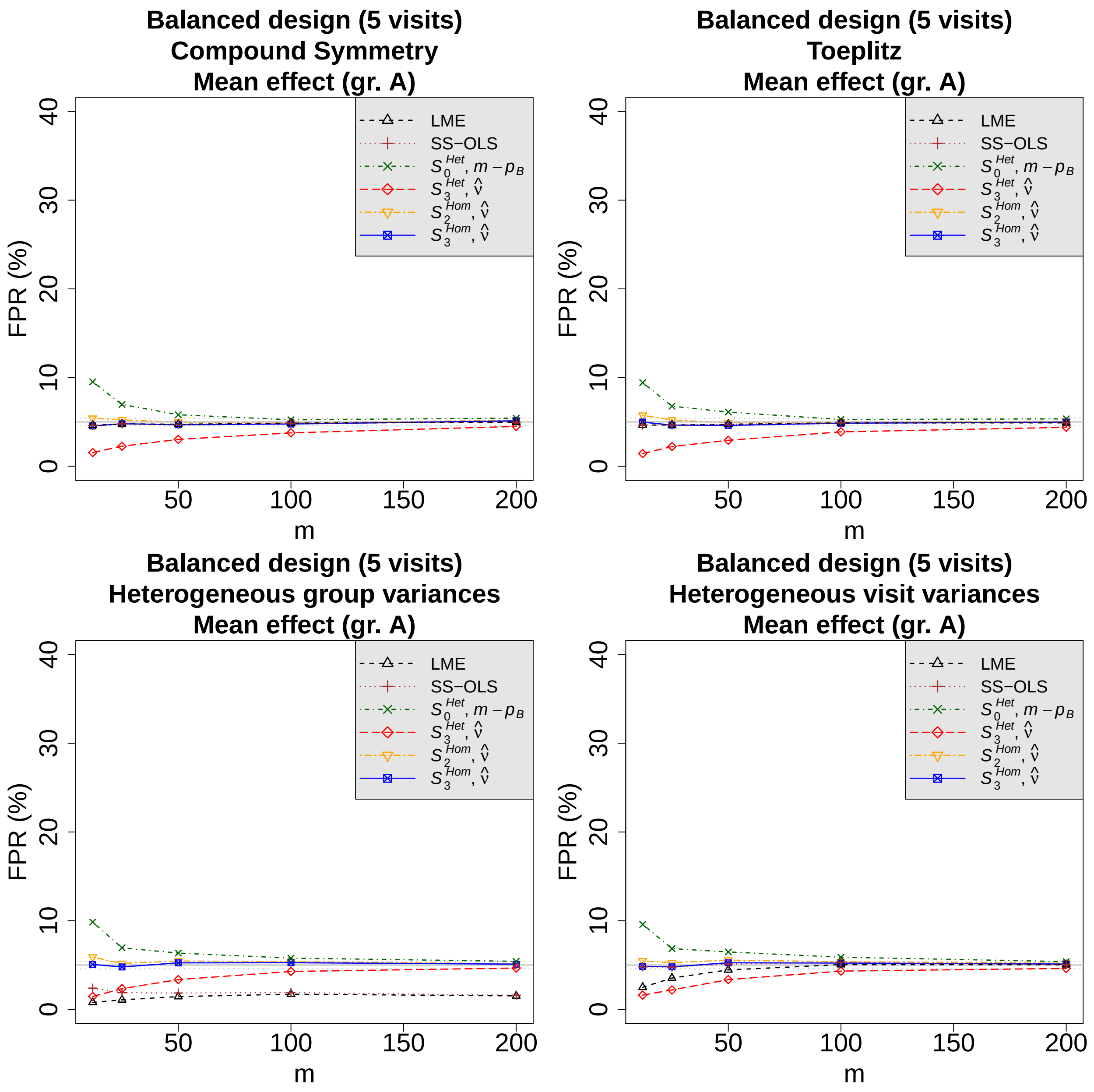

Supplementary Figure 2: FPR comparison on the mean effect of group A with Compound Symmetry (top left, $\rho = 0.95$), Toeplitz (top right, $\psi = 0.1$ per visit), heterogeneous group variances (bottom left, $\alpha_A = 1$ and $\alpha_B = 2$) and heterogeneous visit variances (bottom right, $\gamma = 1$ per visit) for the balanced design with 5 visits per subject; all results are based on an F-test at nominal level 5%; see Supplementary Figure 1 for a description of the SwE versions.

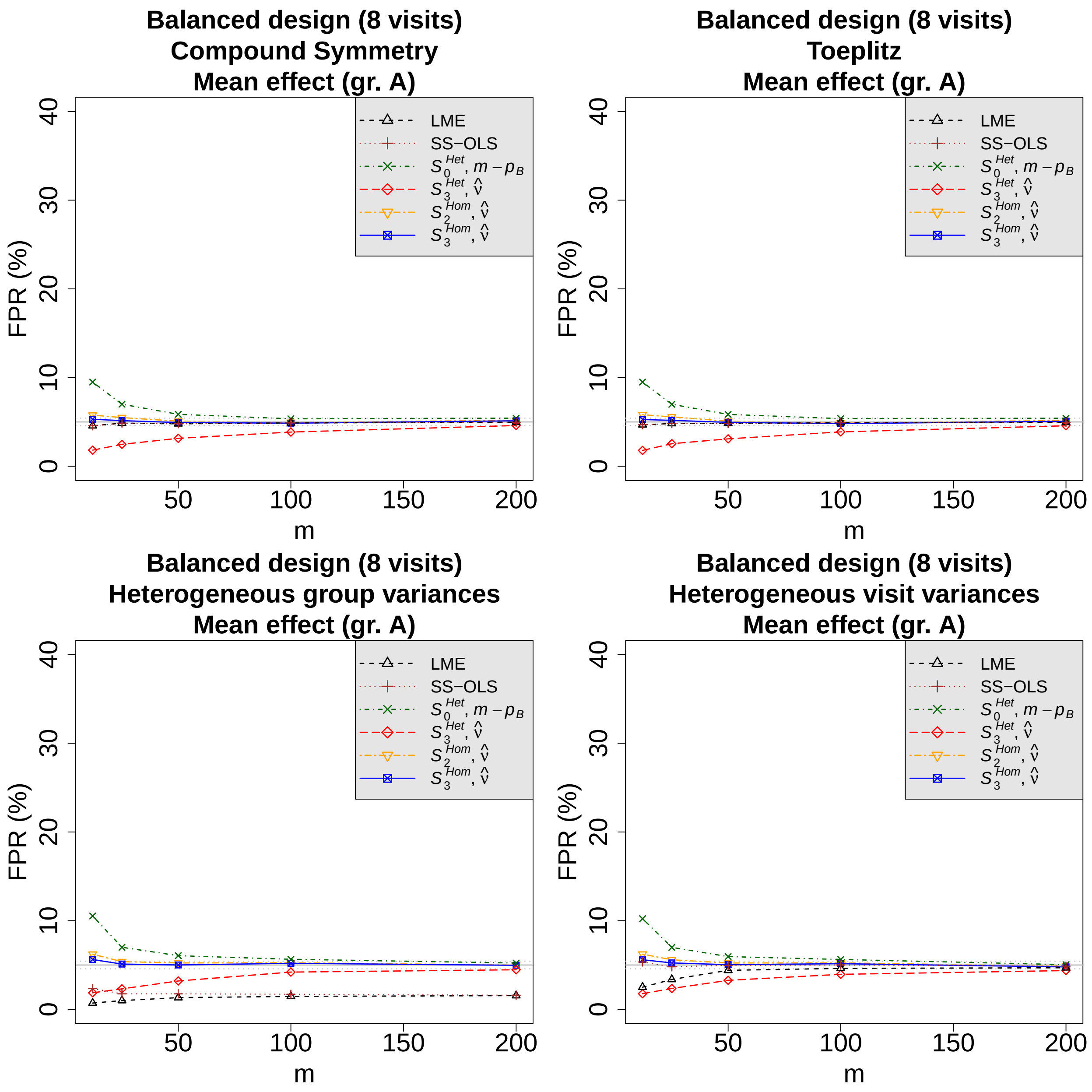

Supplementary Figure 3: FPR comparison on the mean effect of group A with Compound Symmetry (top left, $\rho = 0.95$), Toeplitz (top right, $\psi = 0.1$ per visit), heterogeneous group variances (bottom left, $\alpha_A = 1$ and $\alpha_B = 2$) and heterogeneous visit variances (bottom right, $\gamma = 1$ per visit) for the balanced design with 8 visits per subject; all results are based on an F-test at nominal level 5%; see Supplementary Figure 1 for a description of the SwE versions.

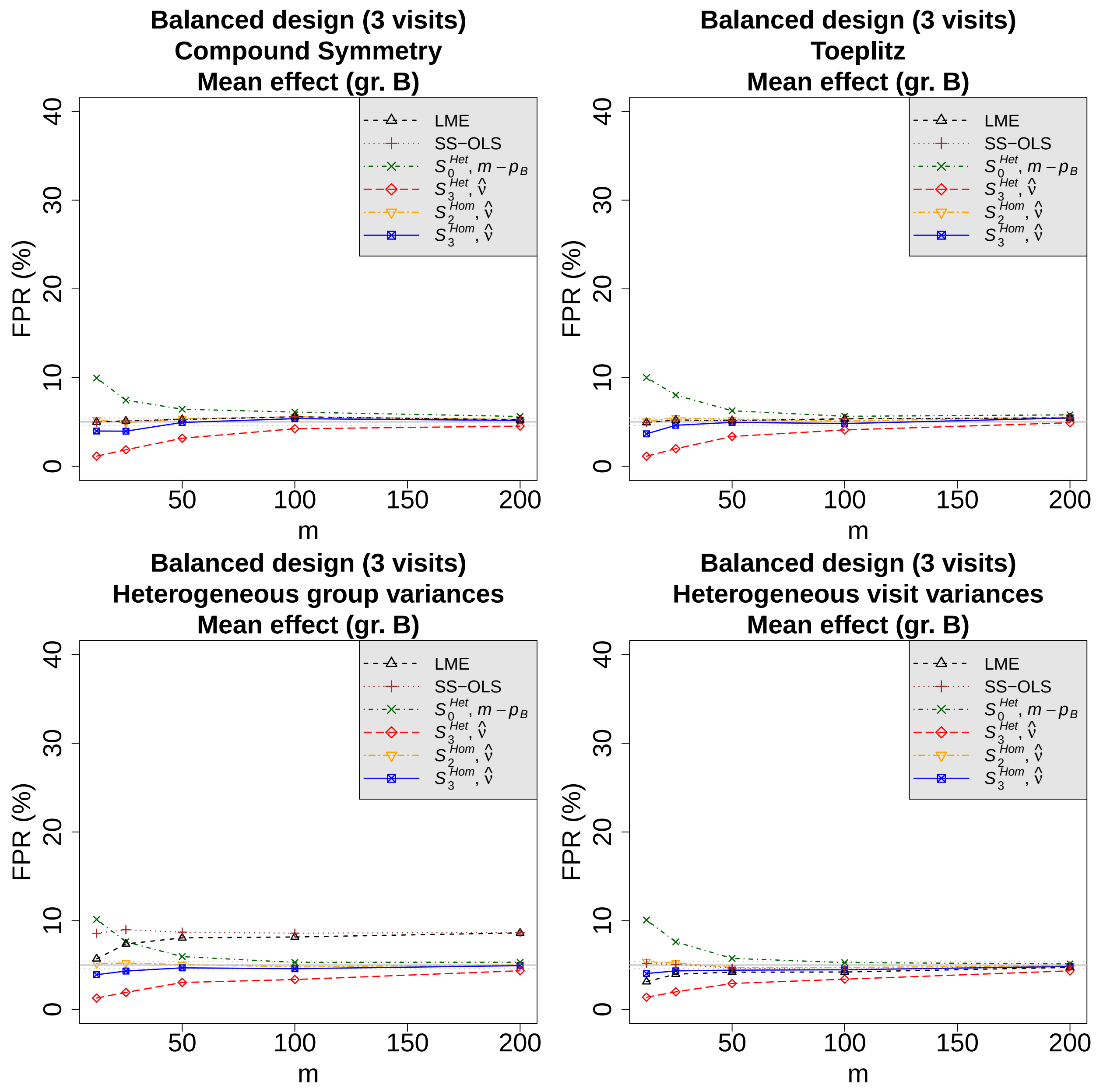

Supplementary Figure 4: FPR comparison on the mean effect of group B with Compound Symmetry (top left, $\rho = 0.95$), Toeplitz (top right, $\psi = 0.1$ per visit), heterogeneous group variances (bottom left, $\alpha_{\mathrm{A}} = 1$ and $\alpha_{\mathrm{B}} = 2$) and heterogeneous visit variances (bottom right, $\gamma = 1$ per visit) for the balanced design with 3 visits per subject; all results are based on an F-test at nominal level 5%; see Supplementary Figure 1 for a description of the SwE versions.

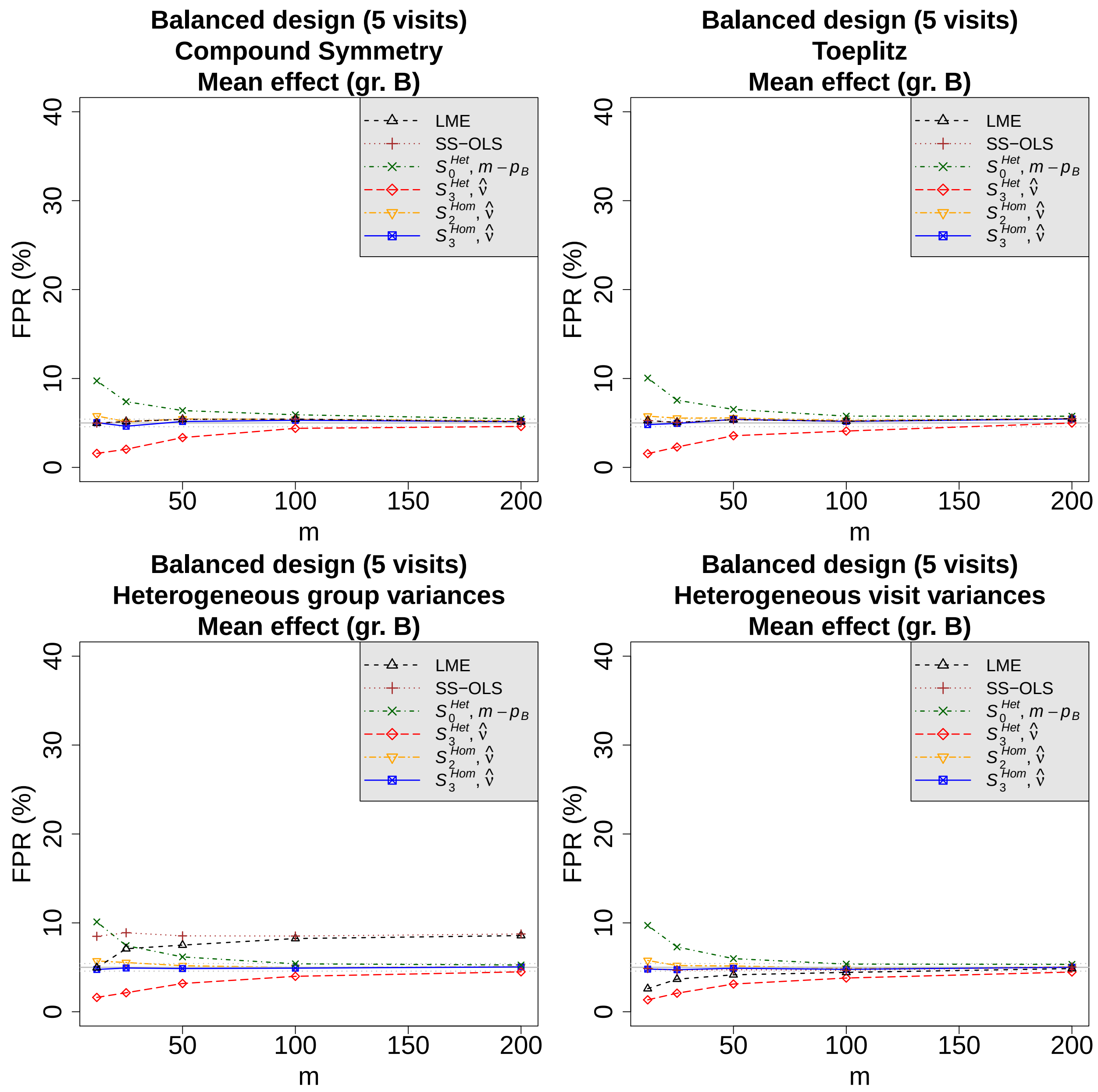

Supplementary Figure 5: FPR comparison on the mean effect of group B with Compound Symmetry (top left, $\rho = 0.95$), Toeplitz (top right, $\psi = 0.1$ per visit), heterogeneous group variances (bottom left, $\alpha_{\mathrm{A}} = 1$ and $\alpha_{\mathrm{B}} = 2$) and heterogeneous visit variances (bottom right, $\gamma = 1$ per visit) for the balanced design with 5 visits per subject; all results are based on an F-test at nominal level 5%; see Supplementary Figure 1 for a description of the SwE versions.

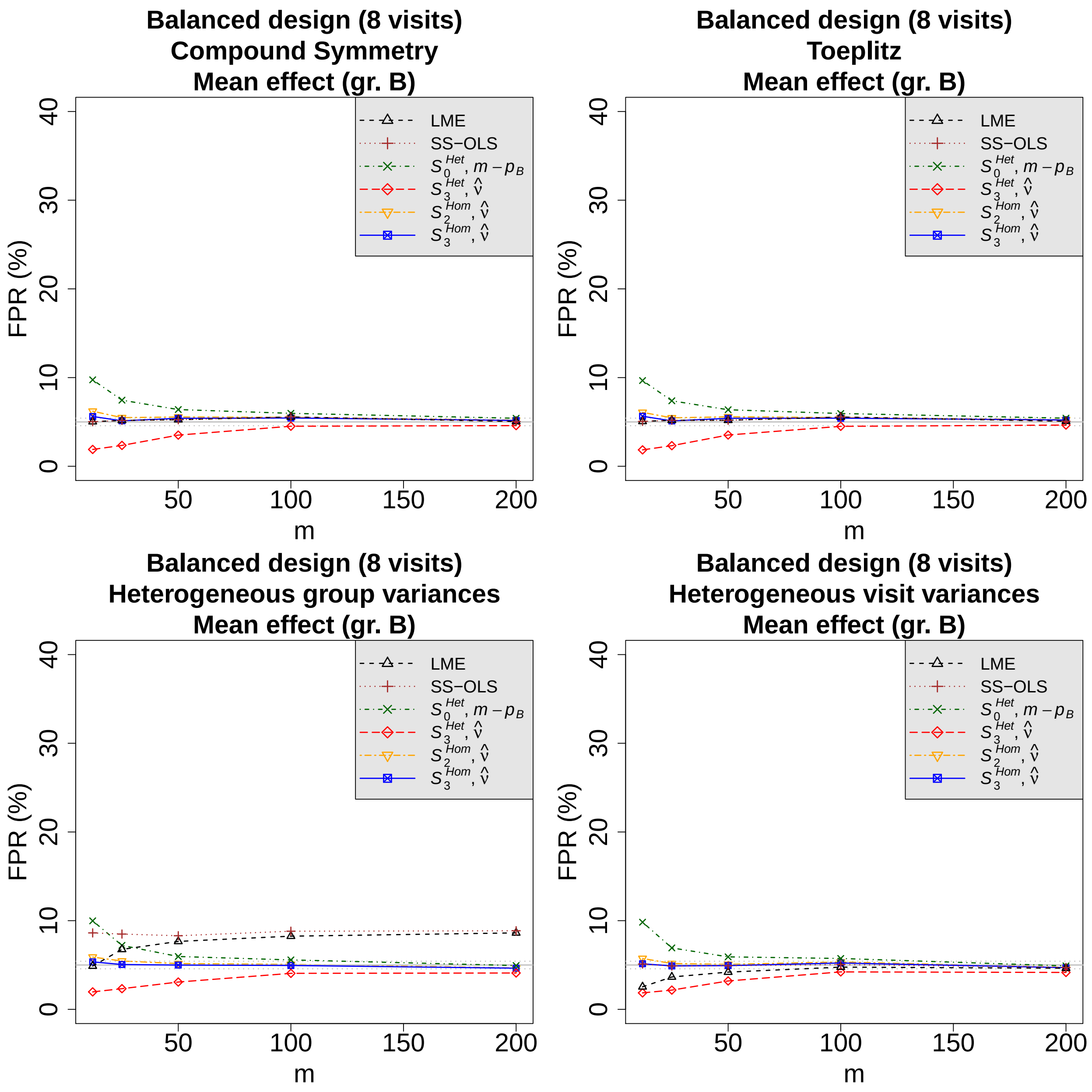

Supplementary Figure 6: FPR comparison on the mean effect of group B with Compound Symmetry (top left, $\rho = 0.95$), Toeplitz (top right, $\psi = 0.1$ per visit), heterogeneous group variances (bottom left, $\alpha_A = 1$ and $\alpha_B = 2$) and heterogeneous visit variances (bottom right, $\gamma = 1$ per visit) for the balanced design with 8 visits per subject; all results are based on an F-test at nominal level 5%; see Supplementary Figure 1 for a description of the SwE versions.

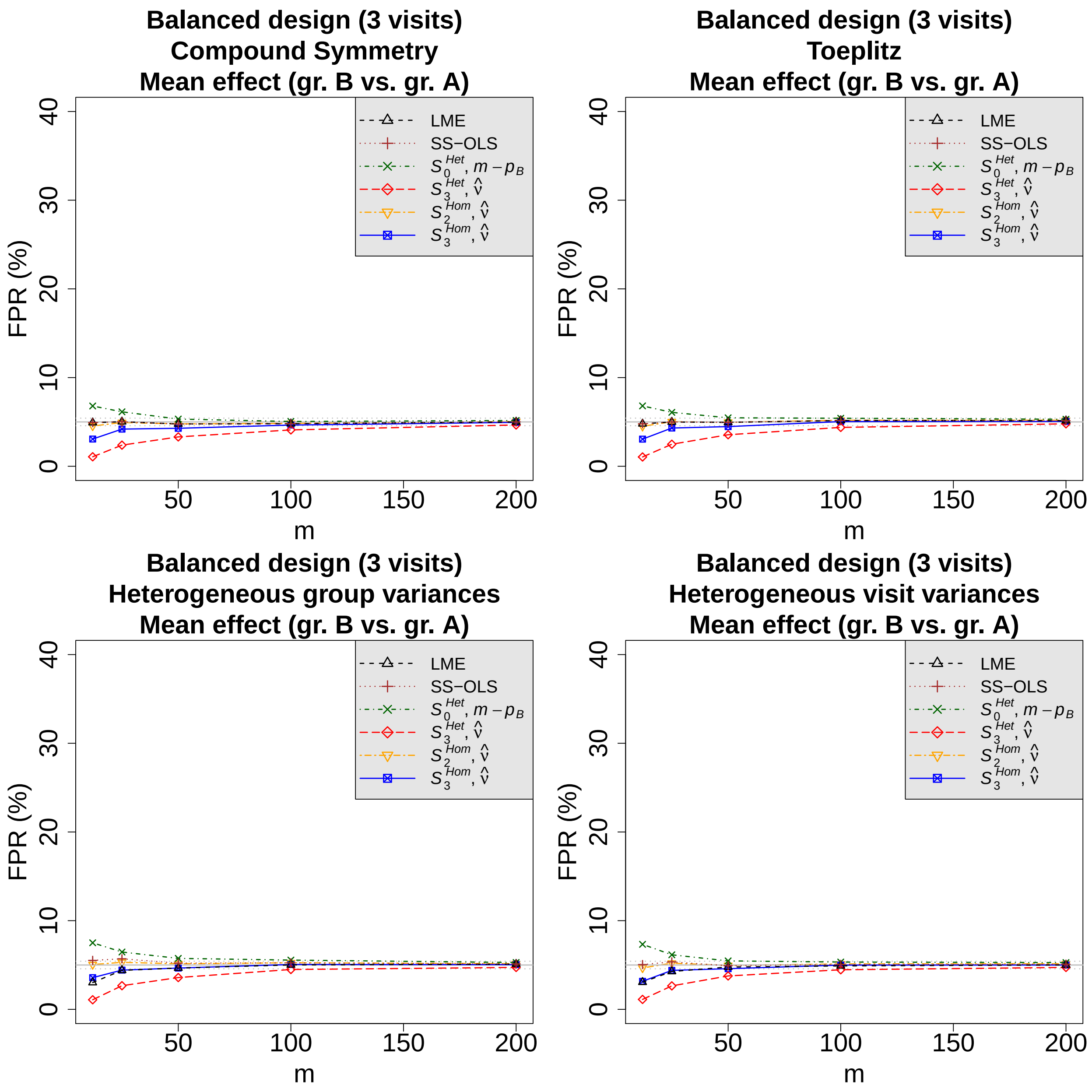

Supplementary Figure 7: FPR comparison on the mean effect difference between group B and group A with Compound Symmetry (top left, $\rho = 0.95$), Toeplitz (top right, $\psi = 0.1$ per visit), heterogeneous group variances (bottom left, $\alpha_A = 1$ and $\alpha_B = 2$) and heterogeneous visit variances (bottom right, $\gamma = 1$ per visit) for the balanced design with 3 visits per subject; all results are based on an F-test at nominal level 5%; see Supplementary Figure 1 for a description of the SwE versions.

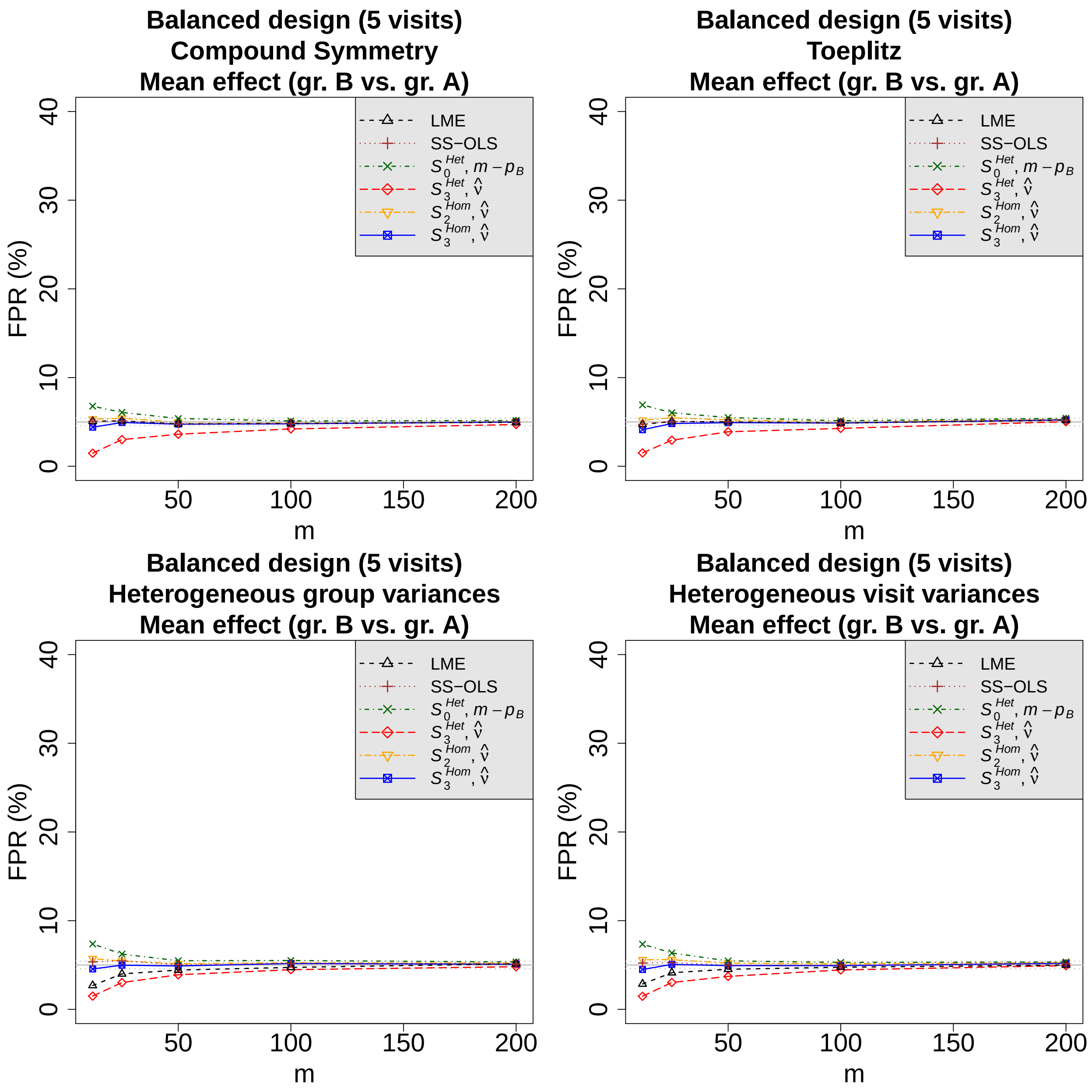

Supplementary Figure 8: FPR comparison on the mean effect difference between group B and group A with Compound Symmetry (top left, $\rho = 0.95$), Toeplitz (top right, $\psi = 0.1$ per visit), heterogeneous group variances (bottom left, $\alpha_A = 1$ and $\alpha_B = 2$) and heterogeneous visit variances (bottom right, $\gamma = 1$ per visit) for the balanced design with 5 visits per subject; all results are based on an F-test at nominal level 5%; see Supplementary Figure 1 for a description of the SwE versions.

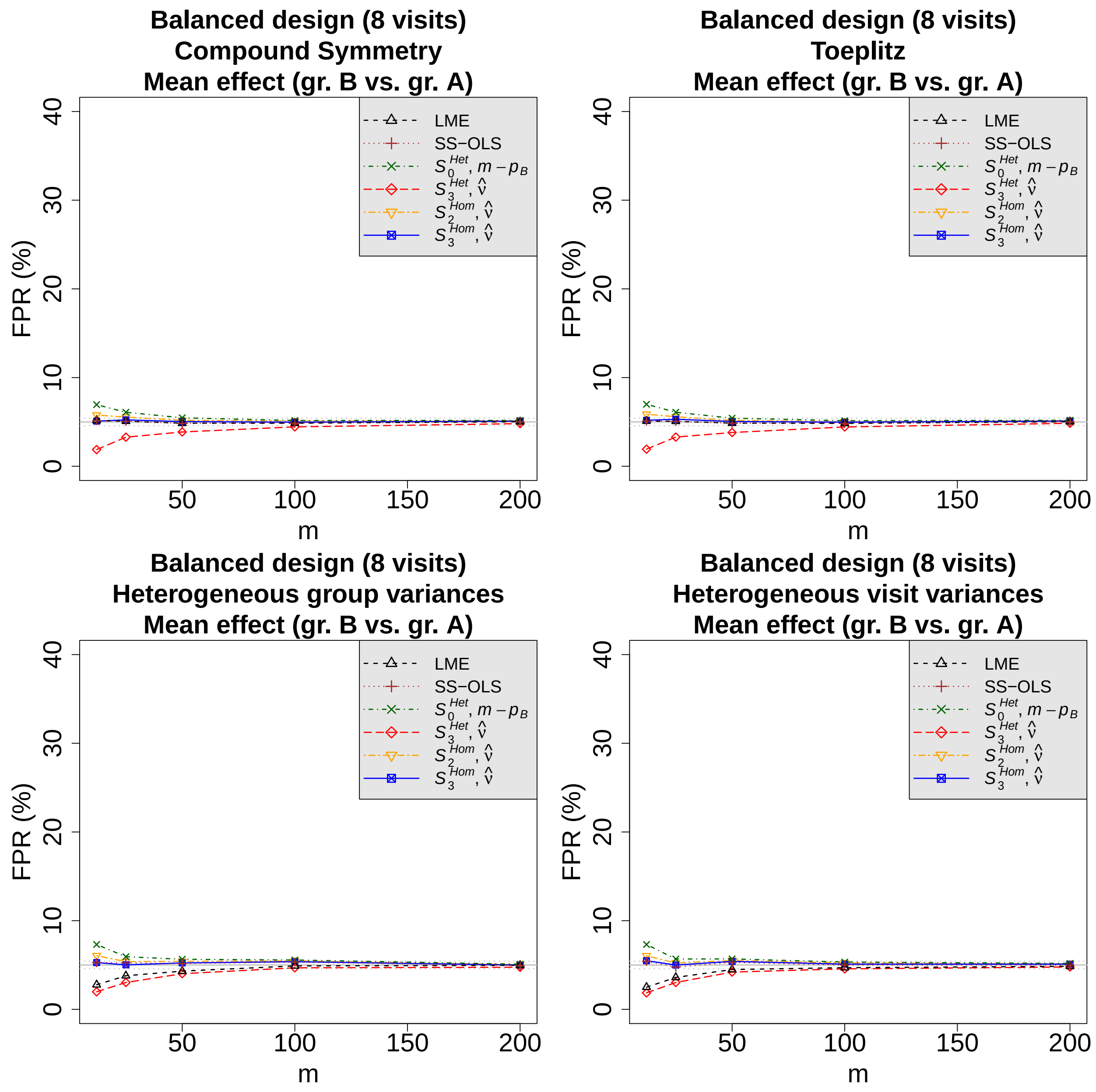

Supplementary Figure 9: FPR comparison on the mean effect difference between group B and group A with Compound Symmetry (top left, $\rho = 0.95$), Toeplitz (top right, $\psi = 0.1$ per visit), heterogeneous group variances (bottom left, $\alpha_{\mathrm{A}} = 1$ and $\alpha_{\mathrm{B}} = 2$) and heterogeneous visit variances (bottom right, $\gamma = 1$ per visit) for the balanced design with 8 visits per subject; all results are based on an F-test at nominal level 5%; see Supplementary Figure 1 for a description of the SwE versions.

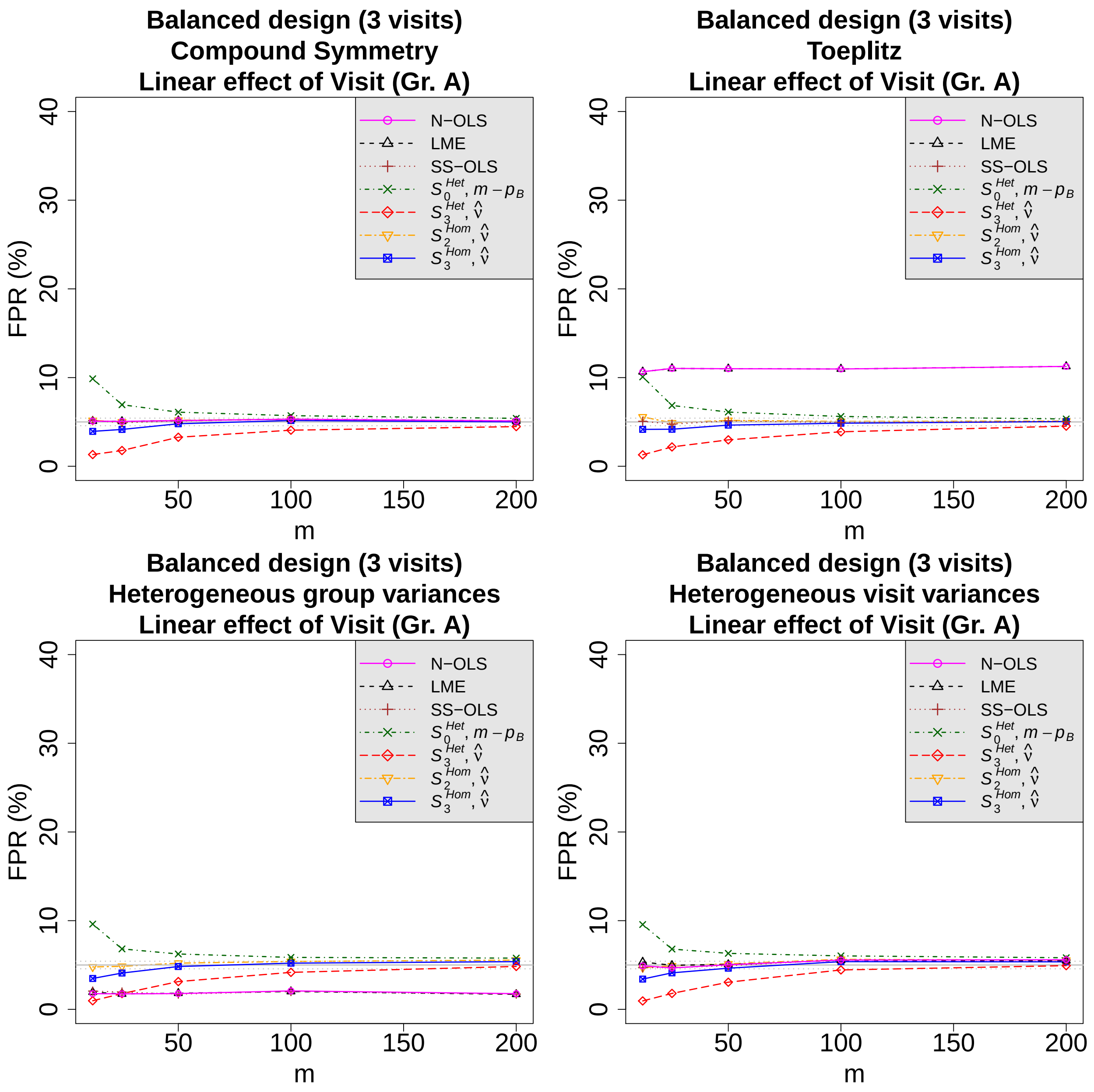

Supplementary Figure 10: FPR comparison on the linear effect of visit of group A with Compound Symmetry (top left, $\rho = 0.95$), Toeplitz (top right, $\psi = 0.1$ per visit), heterogeneous group variances (bottom left, $\alpha_{\mathrm{A}} = 1$ and $\alpha_{\mathrm{B}} = 2$) and heterogeneous visit variances (bottom right, $\gamma = 1$ per visit) for the balanced design with 3 visits per subject; all results are based on an F-test at nominal level 5%; see Supplementary Figure 1 for a description of the SwE versions.

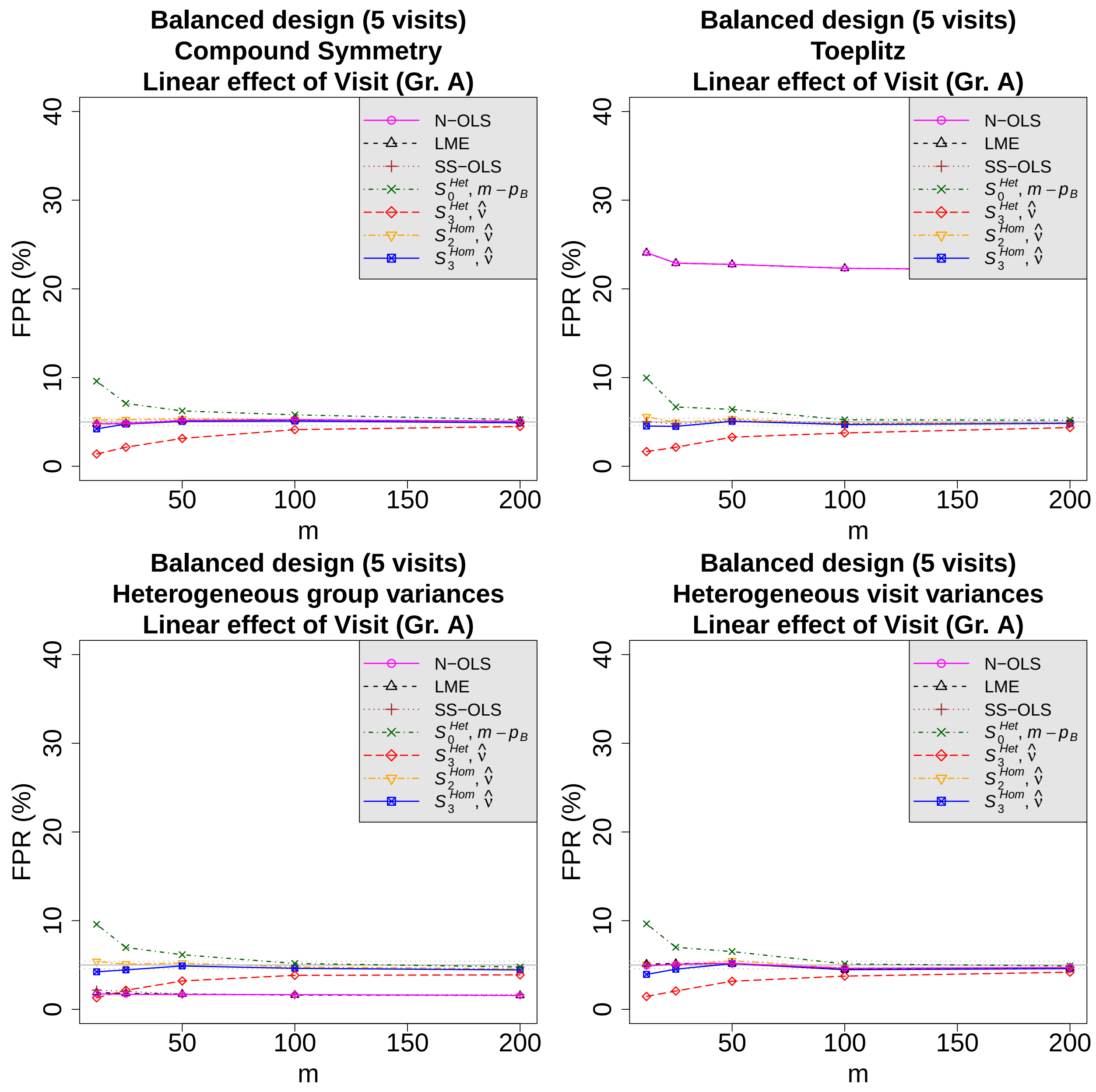

Supplementary Figure 11: FPR comparison on the linear effect of visit of group A with Compound Symmetry (top left, $\rho = 0.95$), Toeplitz (top right, $\psi = 0.1$ per visit), heterogeneous group variances (bottom left, $\alpha_A = 1$ and $\alpha_B = 2$) and heterogeneous visit variances (bottom right, $\gamma = 1$ per visit) for the balanced design with 5 visits per subject; all results are based on an F-test at nominal level 5%; see Supplementary Figure 1 for a description of the SwE versions.

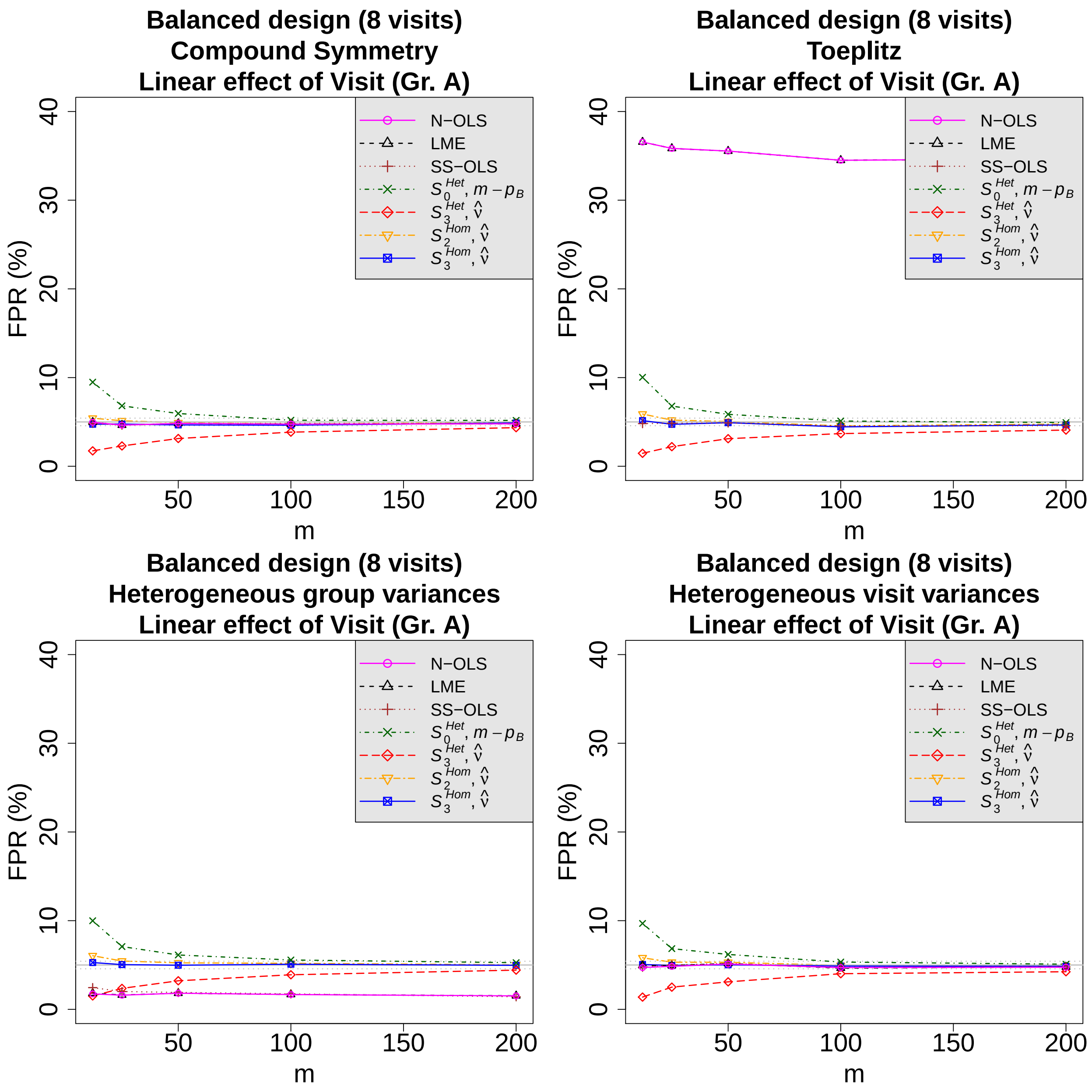

Supplementary Figure 12: FPR comparison on the linear effect of visit of group A with Compound Symmetry (top left, $\rho = 0.95$), Toeplitz (top right, $\psi = 0.1$ per visit), heterogeneous group variances (bottom left, $\alpha_A = 1$ and $\alpha_B = 2$) and heterogeneous visit variances (bottom right, $\gamma = 1$ per visit) for the balanced design with 8 visits per subject; all results are based on an F-test at nominal level 5%; see Supplementary Figure 1 for a description of the SwE versions.

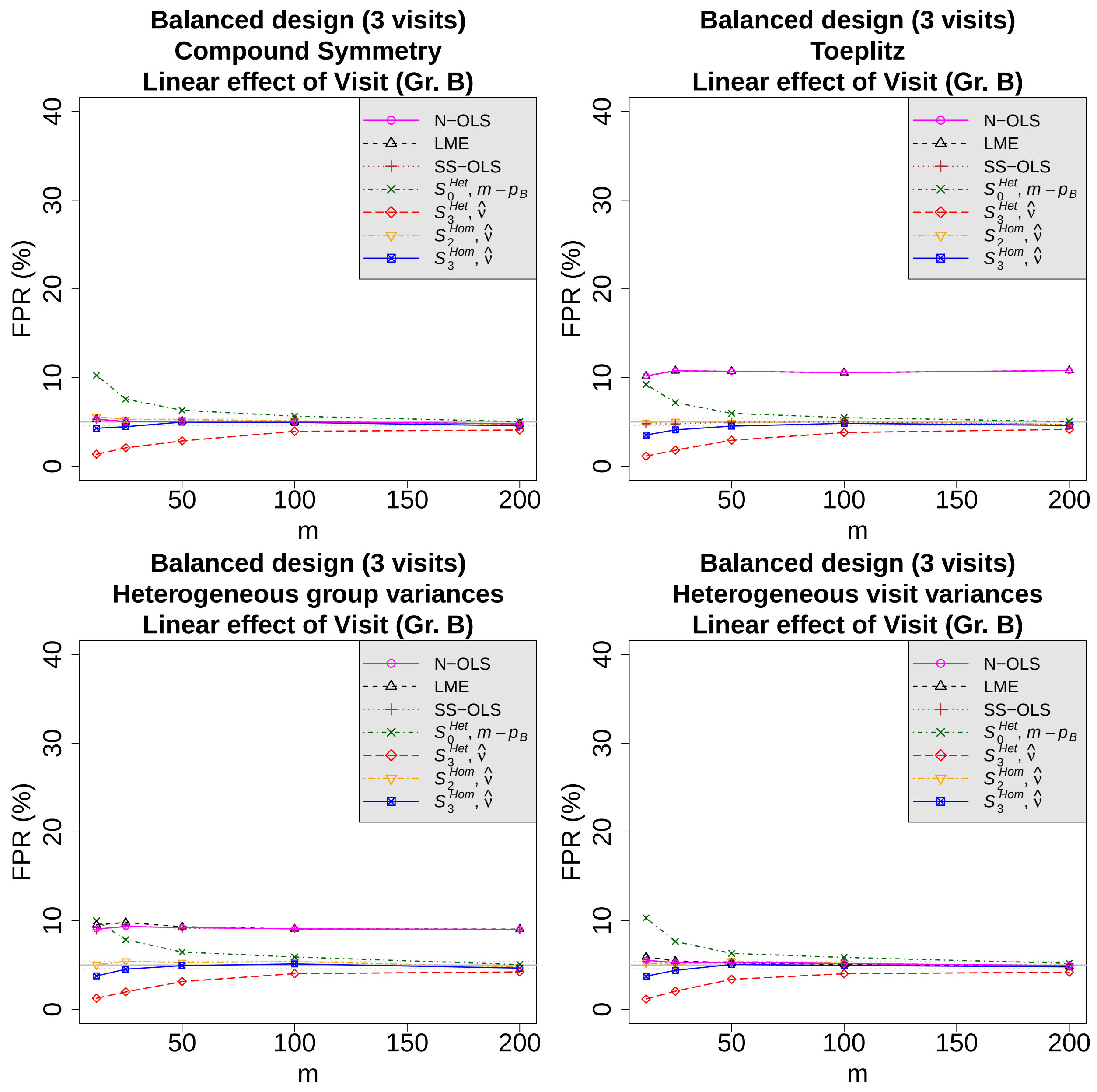

Supplementary Figure 13: FPR comparison on the linear effect of visit of group B with Compound Symmetry (top left, $\rho = 0.95$), Toeplitz (top right, $\psi = 0.1$ per visit), heterogeneous group variances (bottom left, $\alpha_A = 1$ and $\alpha_B = 2$) and heterogeneous visit variances (bottom right, $\gamma = 1$ per visit) for the balanced design with 3 visits per subject; all results are based on an F-test at nominal level 5%; see Supplementary Figure 1 for a description of the SwE versions.

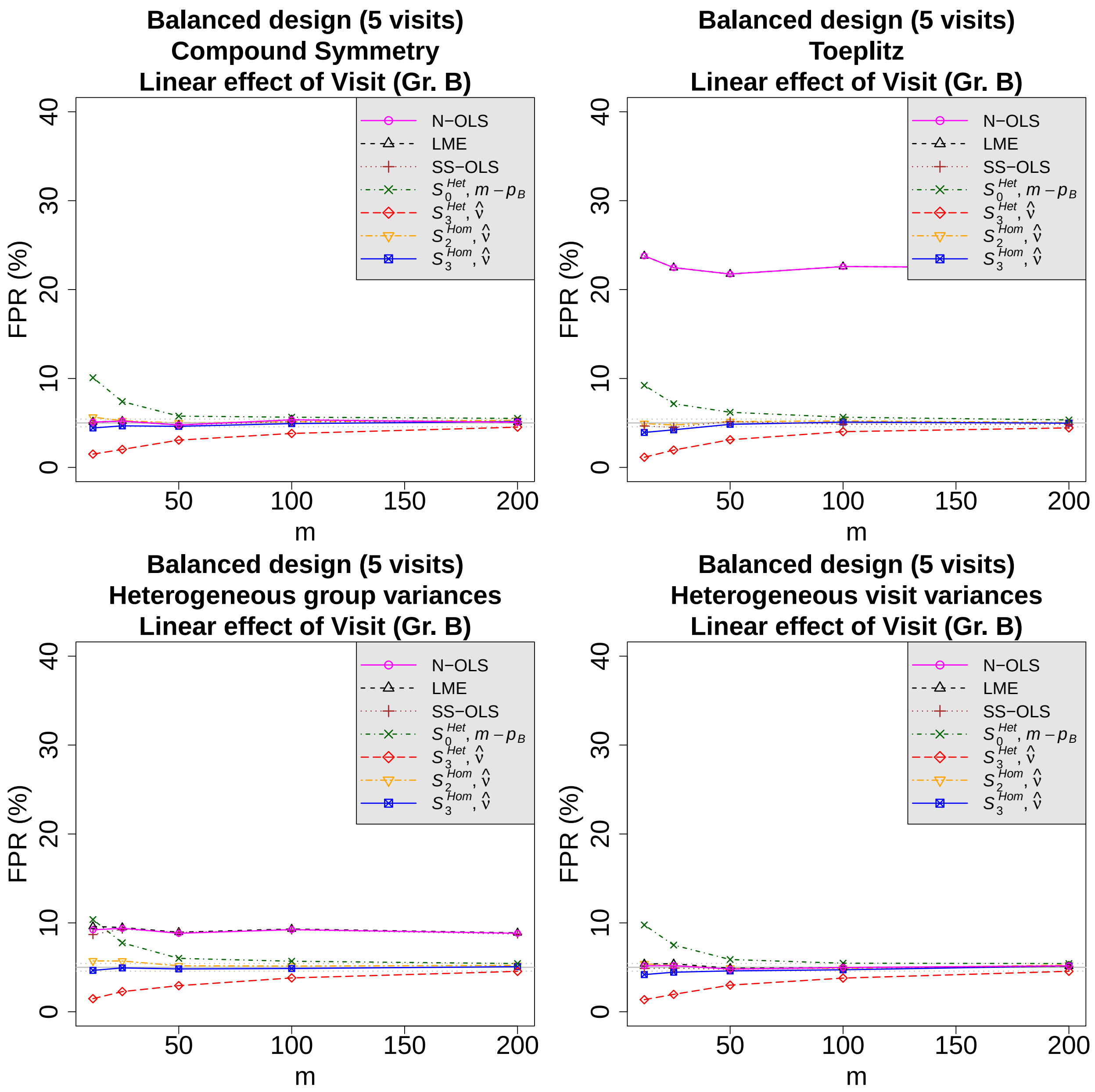

Supplementary Figure 14: FPR comparison on the linear effect of visit of group B with Compound Symmetry (top left, $\rho = 0.95$), Toeplitz (top right, $\psi = 0.1$ per visit), heterogeneous group variances (bottom left, $\alpha_{\mathrm{A}} = 1$ and $\alpha_{\mathrm{B}} = 2$) and heterogeneous visit variances (bottom right, $\gamma = 1$ per visit) for the balanced design with 5 visits per subject; all results are based on an F-test at nominal level 5%; see Supplementary Figure 1 for a description of the SwE versions.

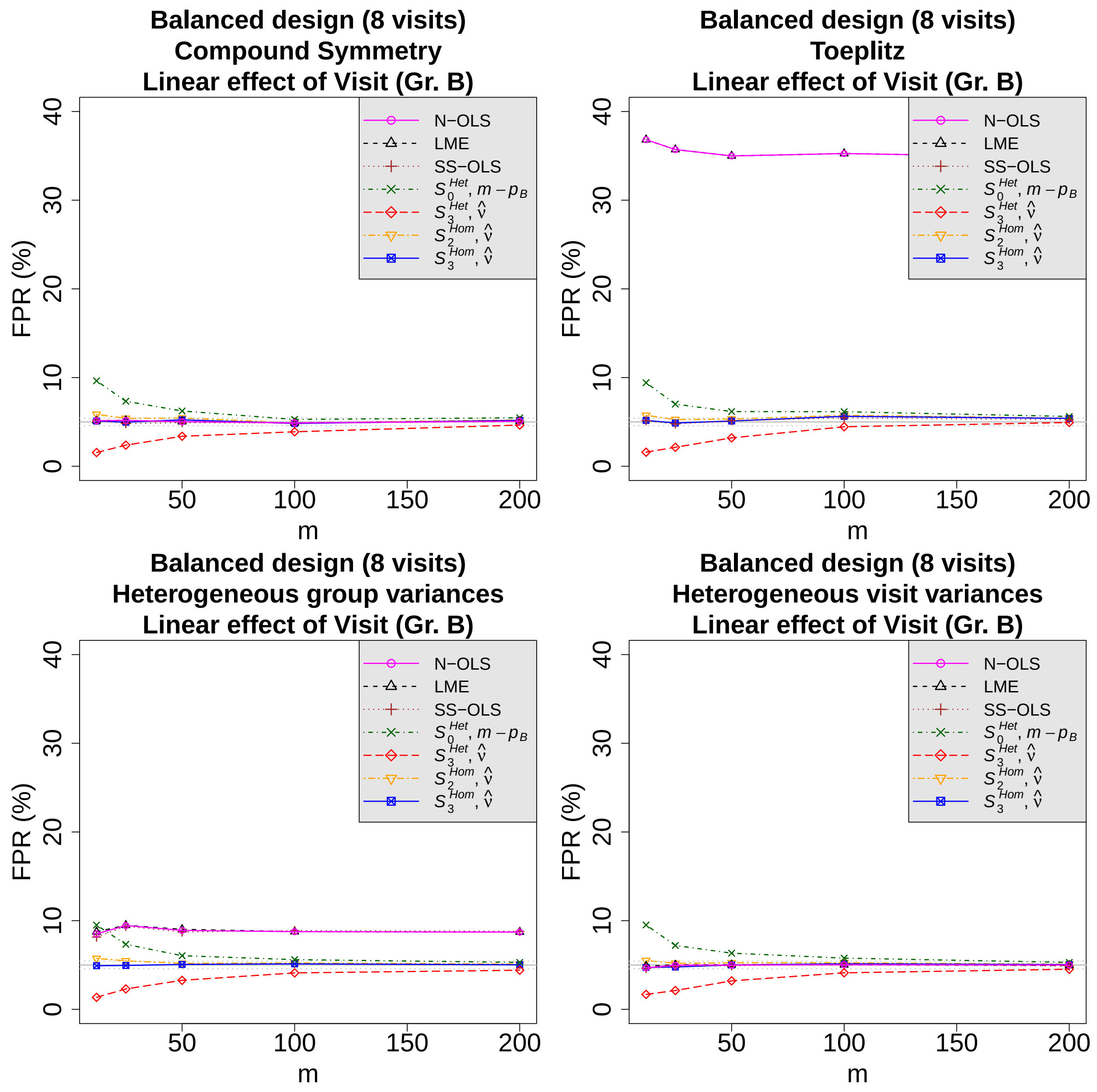

Supplementary Figure 15: FPR comparison on the linear effect of visit of group B with Compound Symmetry (top left, $\rho = 0.95$), Toeplitz (top right, $\psi = 0.1$ per visit), heterogeneous group variances (bottom left, $\alpha_{\mathrm{A}} = 1$ and $\alpha_{\mathrm{B}} = 2$) and heterogeneous visit variances (bottom right, $\gamma = 1$ per visit) for the balanced design with 8 visits per subject; all results are based on an F-test at nominal level 5%; see Supplementary Figure 1 for a description of the SwE versions.

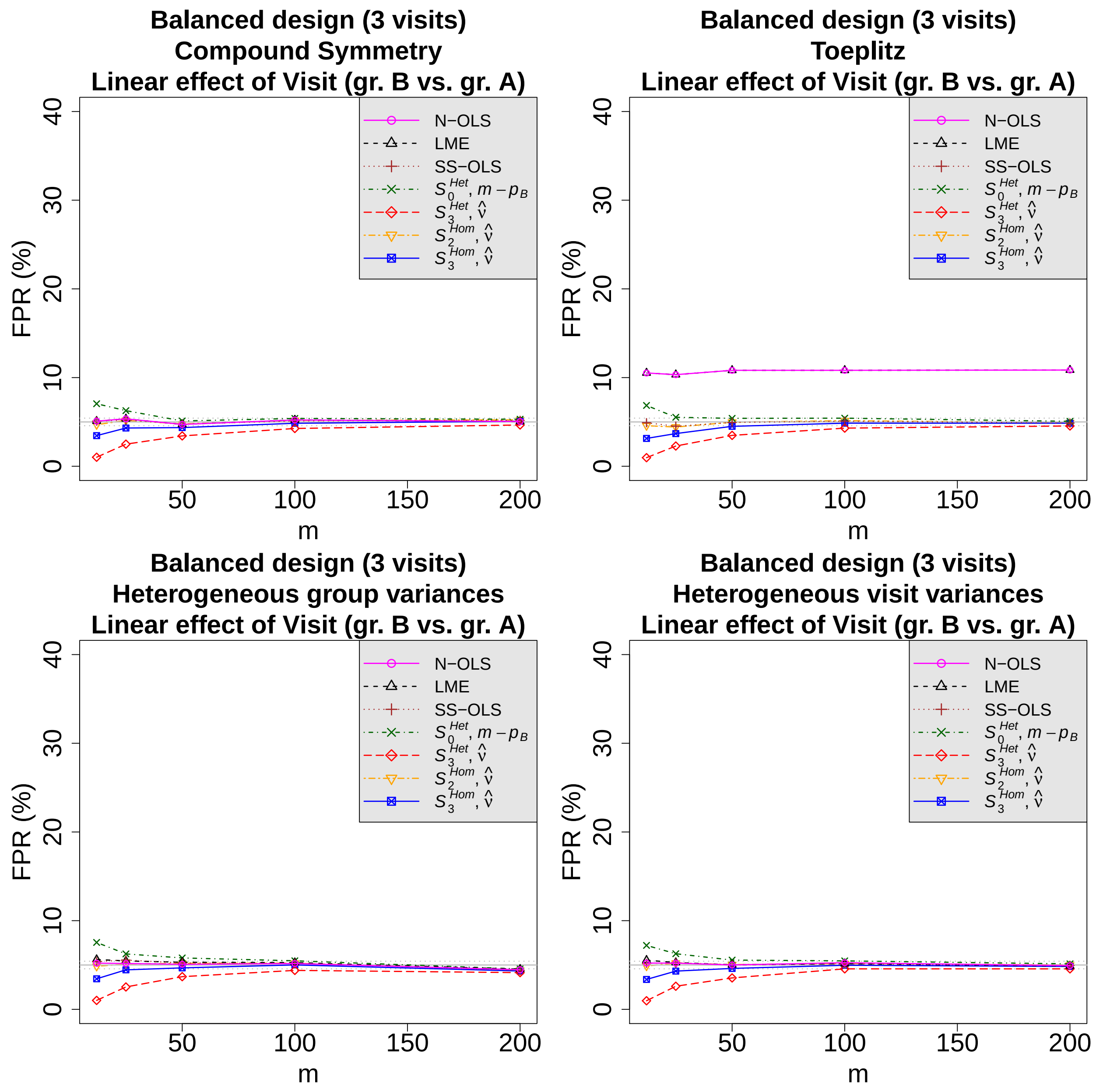

Supplementary Figure 16: FPR comparison on the linear effect of visit difference between group B and group A with Compound Symmetry (top left, $\rho = 0.95$), Toeplitz (top right, $\psi = 0.1$ per visit), heterogeneous group variances (bottom left, $\alpha_A = 1$ and $\alpha_B = 2$) and heterogeneous visit variances (bottom right, $\gamma = 1$ per visit) for the balanced design with 3 visits per subject; all results are based on an F-test at nominal level 5%; see Supplementary Figure 1 for a description of the SwE versions.

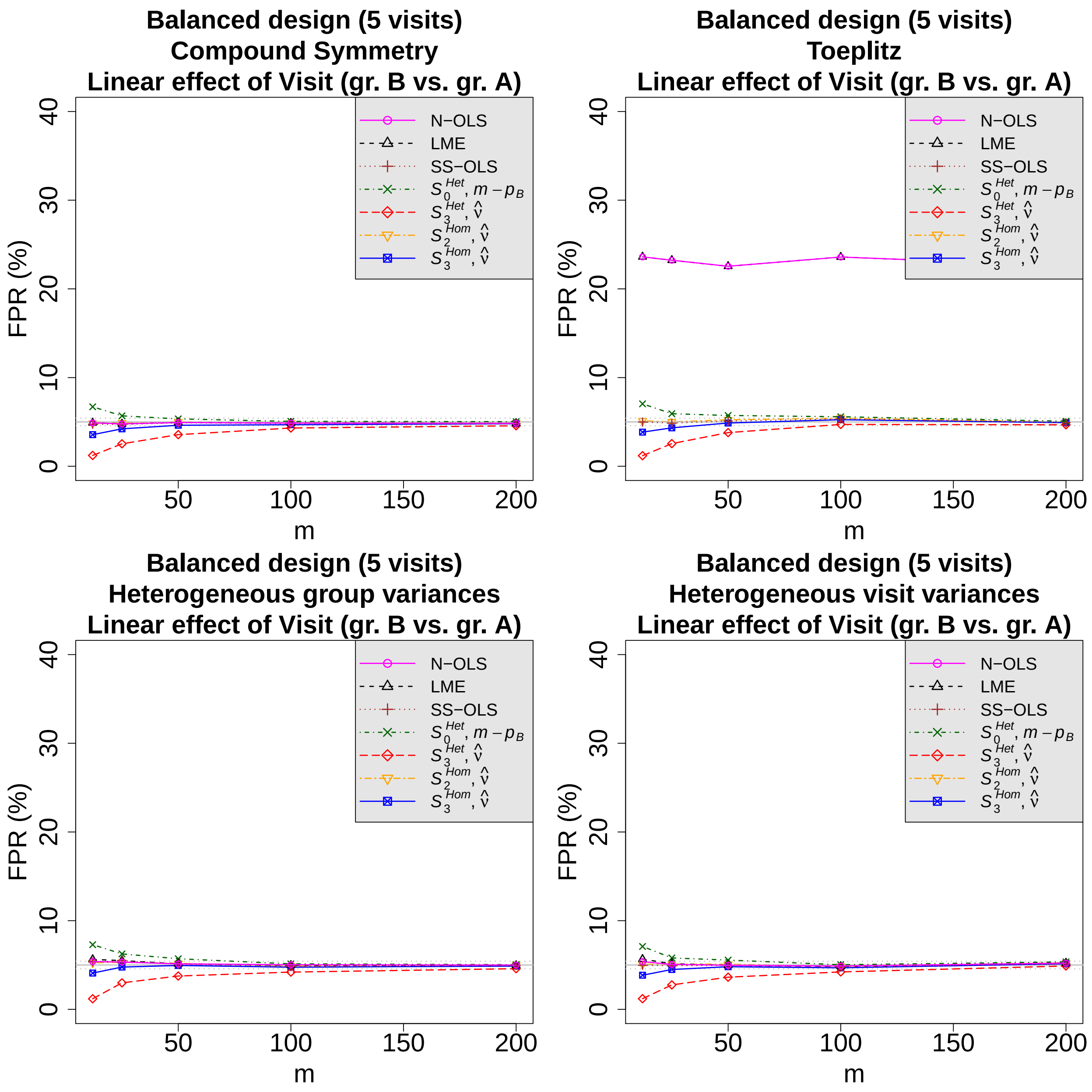

Supplementary Figure 17: FPR comparison on the linear effect of visit difference between group B and group A with Compound Symmetry (top left, $\rho = 0.95$), Toeplitz (top right, $\psi = 0.1$ per visit), heterogeneous group variances (bottom left, $\alpha_{\mathrm{A}} = 1$ and $\alpha_{\mathrm{B}} = 2$) and heterogeneous visit variances (bottom right, $\gamma = 1$ per visit) for the balanced design with 5 visits per subject; all results are based on an F-test at nominal level 5%; see Supplementary Figure 1 for a description of the SwE versions.

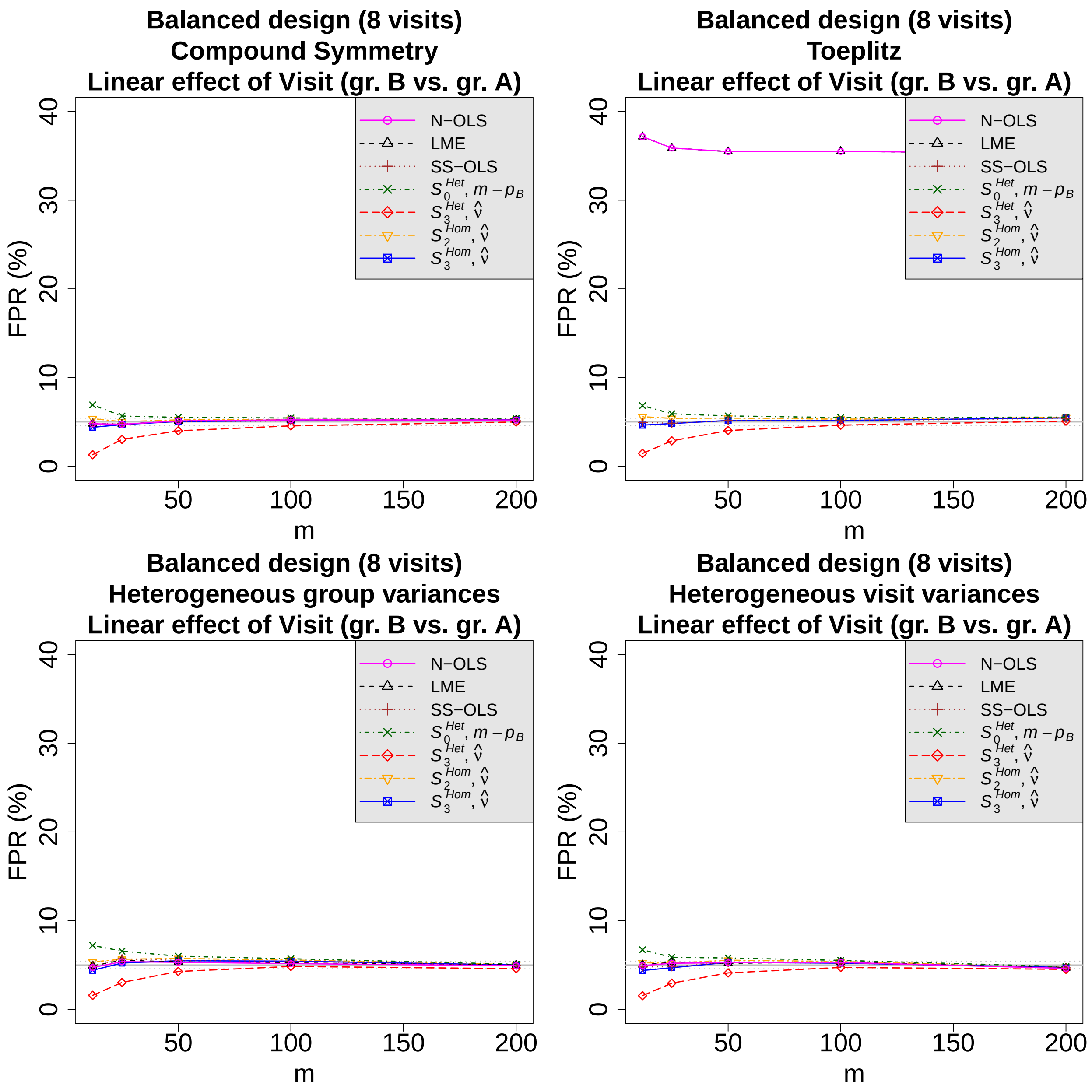

Supplementary Figure 18: FPR comparison on the linear effect of visit difference between group B and group A with Compound Symmetry (top left, $\rho = 0.95$), Toeplitz (top right, $\psi = 0.1$ per visit), heterogeneous group variances (bottom left, $\alpha_A = 1$ and $\alpha_B = 2$) and heterogeneous visit variances (bottom right, $\gamma = 1$ per visit) for the balanced design with 8 visits per subject; all results are based on an F-test at nominal level 5%; see Supplementary Figure 1 for a description of the SwE versions.

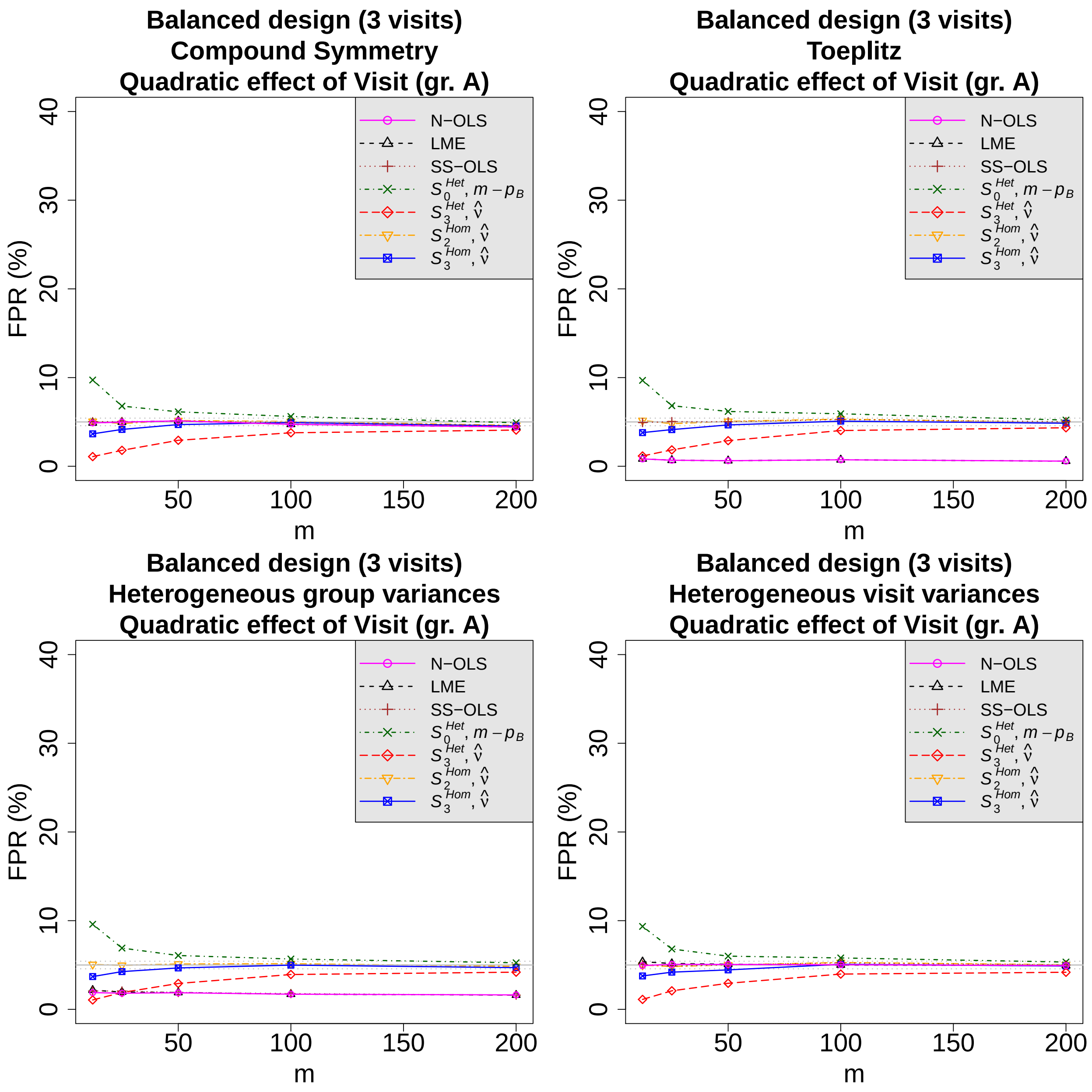

Supplementary Figure 19: FPR comparison on the quadratic effect of visit of group A with Compound Symmetry (top left, $\rho = 0.95$), Toeplitz (top right, $\psi = 0.1$ per visit), heterogeneous group variances (bottom left, $\alpha_{\mathrm{A}} = 1$ and $\alpha_{\mathrm{B}} = 2$) and heterogeneous visit variances (bottom right, $\gamma = 1$ per visit) for the balanced design with 3 visits per subject; all results are based on an F-test at nominal level 5%; see Supplementary Figure 1 for a description of the SwE versions.

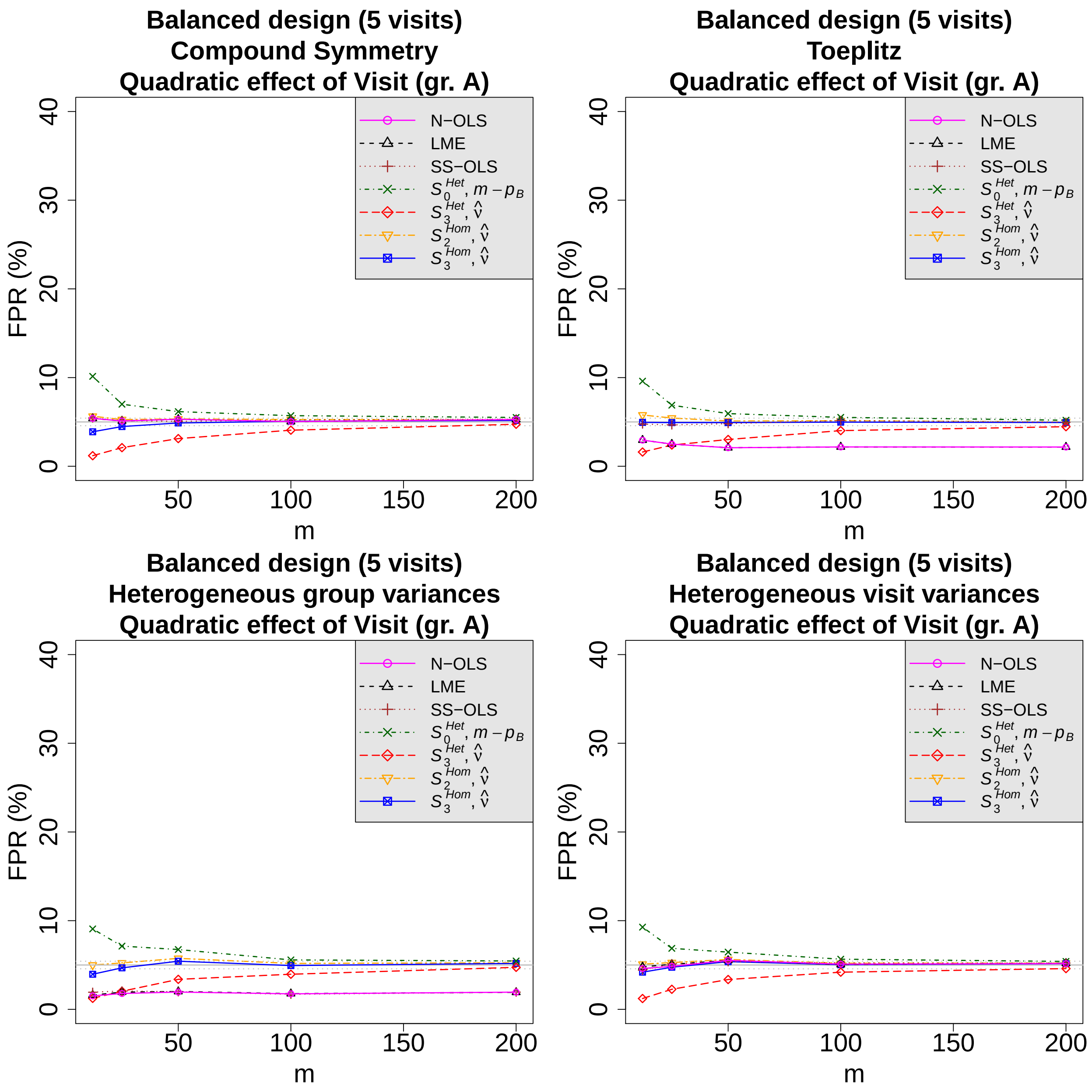

Supplementary Figure 20: FPR comparison on the quadratic effect of visit of group A with Compound Symmetry (top left, $\rho = 0.95$), Toeplitz (top right, $\psi = 0.1$ per visit), heterogeneous group variances (bottom left, $\alpha_A = 1$ and $\alpha_B = 2$) and heterogeneous visit variances (bottom right, $\gamma = 1$ per visit) for the balanced design with 5 visits per subject; all results are based on an F-test at nominal level 5%; see Supplementary Figure 1 for a description of the SwE versions.

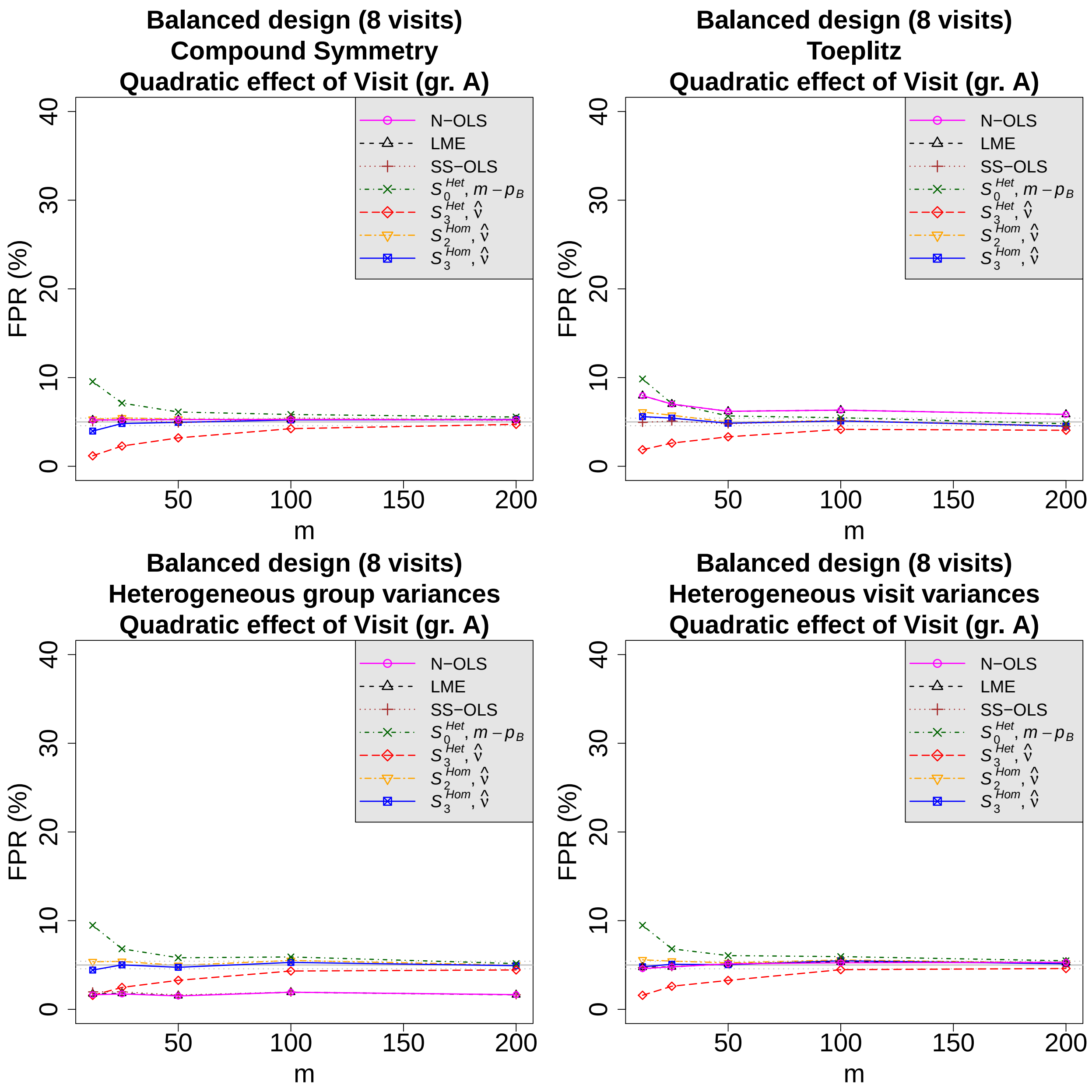

Supplementary Figure 21: FPR comparison on the quadratic effect of visit of group A with Compound Symmetry (top left, $\rho = 0.95$), Toeplitz (top right, $\psi = 0.1$ per visit), heterogeneous group variances (bottom left, $\alpha_{\mathrm{A}} = 1$ and $\alpha_{\mathrm{B}} = 2$) and heterogeneous visit variances (bottom right, $\gamma = 1$ per visit) for the balanced design with 8 visits per subject; all results are based on an F-test at nominal level 5%; see Supplementary Figure 1 for a description of the SwE versions.

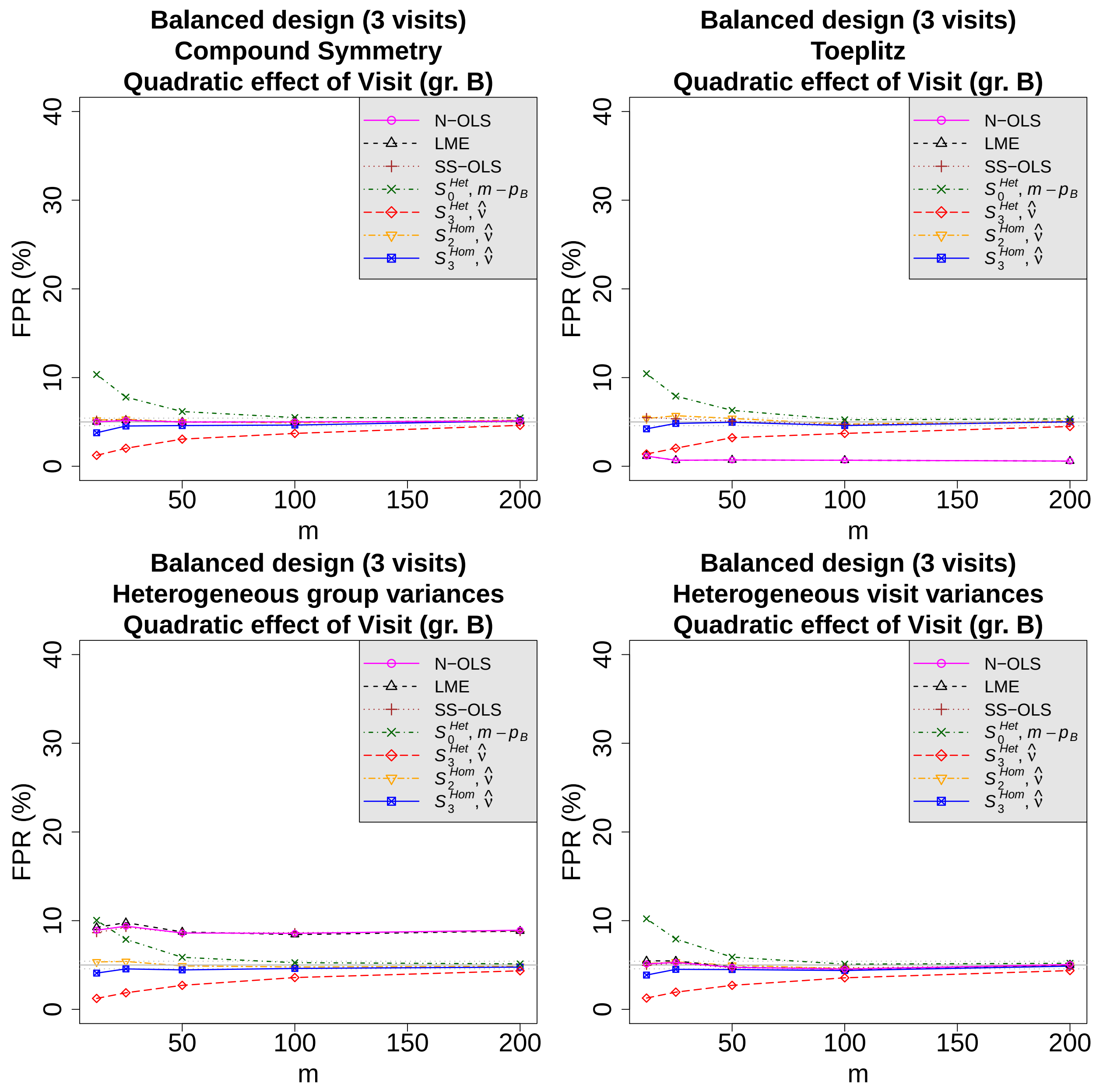

Supplementary Figure 22: FPR comparison on the quadratic effect of visit of group B with Compound Symmetry (top left, $\rho = 0.95$), Toeplitz (top right, $\psi = 0.1$ per visit), heterogeneous group variances (bottom left, $\alpha_{\mathrm{A}} = 1$ and $\alpha_{\mathrm{B}} = 2$) and heterogeneous visit variances (bottom right, $\gamma = 1$ per visit) for the balanced design with 3 visits per subject; all results are based on an F-test at nominal level 5%; see Supplementary Figure 1 for a description of the SwE versions.

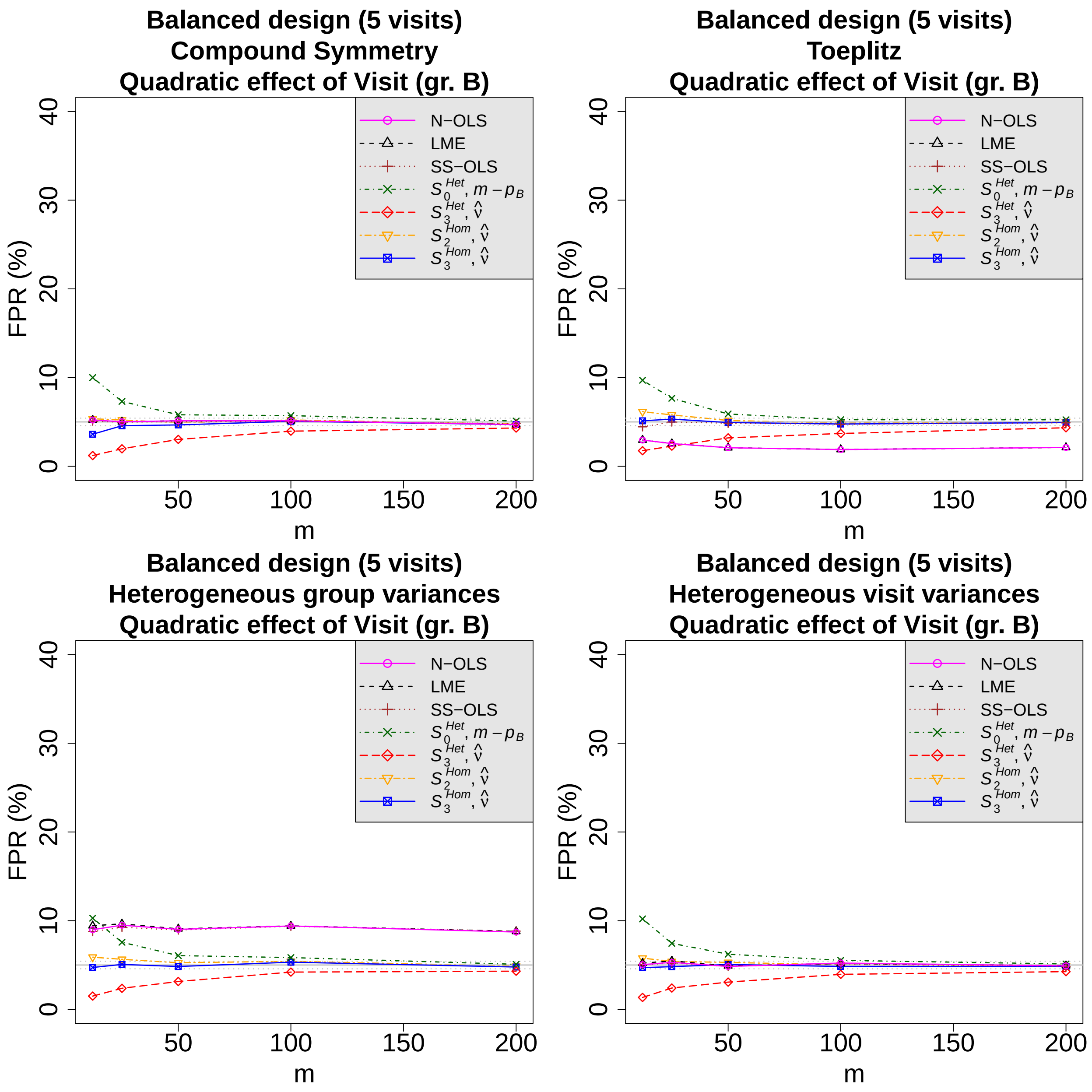

Supplementary Figure 23: FPR comparison on the quadratic effect of visit of group B with Compound Symmetry (top left, $\rho = 0.95$), Toeplitz (top right, $\psi = 0.1$ per visit), heterogeneous group variances (bottom left, $\alpha_{\mathrm{A}} = 1$ and $\alpha_{\mathrm{B}} = 2$) and heterogeneous visit variances (bottom right, $\gamma = 1$ per visit) for the balanced design with 5 visits per subject; all results are based on an F-test at nominal level 5%; see Supplementary Figure 1 for a description of the SwE versions.

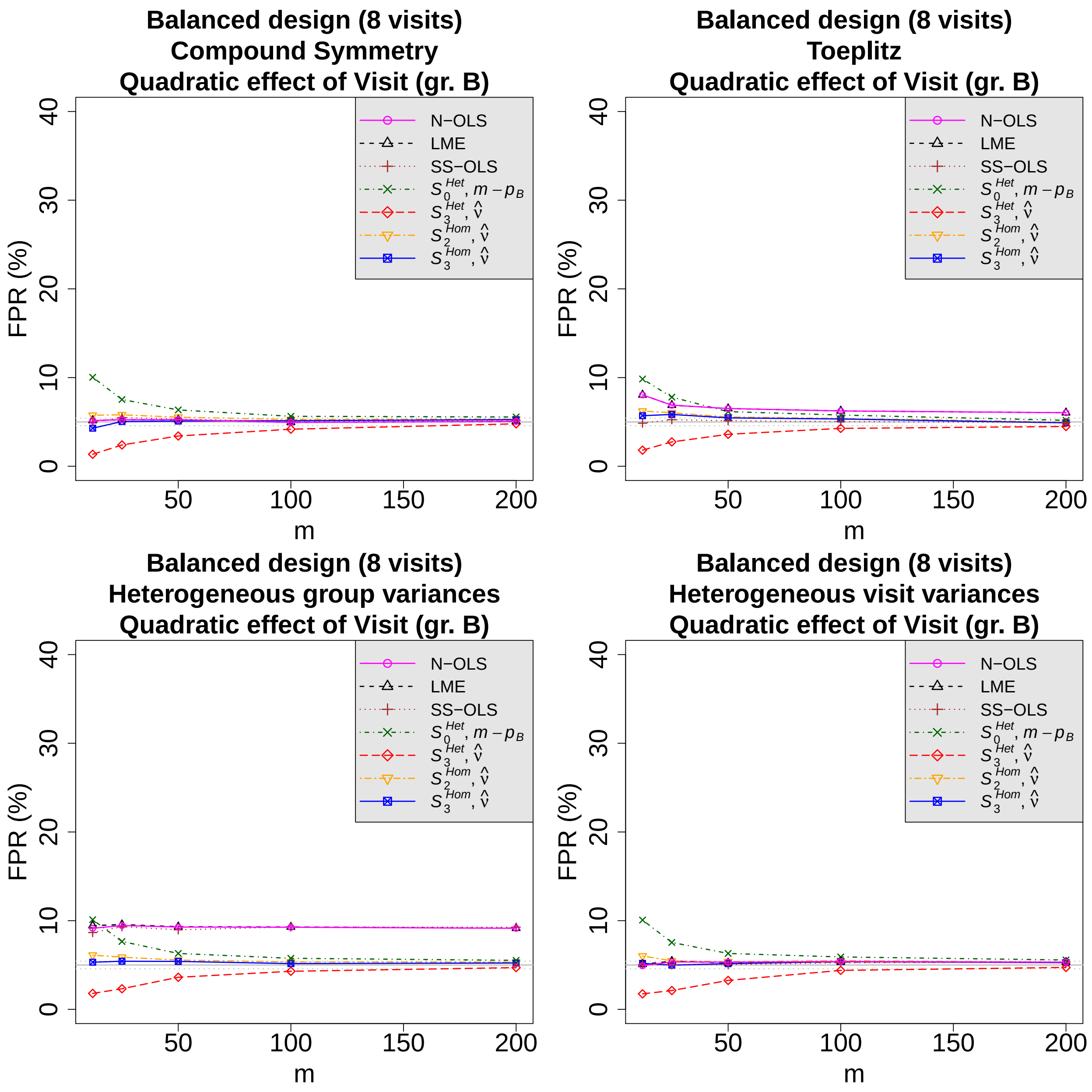

Supplementary Figure 24: FPR comparison on the quadratic effect of visit of group B with Compound Symmetry (top left, $\rho = 0.95$), Toeplitz (top right, $\psi = 0.1$ per visit), heterogeneous group variances (bottom left, $\alpha_{\mathrm{A}} = 1$ and $\alpha_{\mathrm{B}} = 2$) and heterogeneous visit variances (bottom right, $\gamma = 1$ per visit) for the balanced design with 8 visits per subject; all results are based on an F-test at nominal level 5%; see Supplementary Figure 1 for a description of the SwE versions.

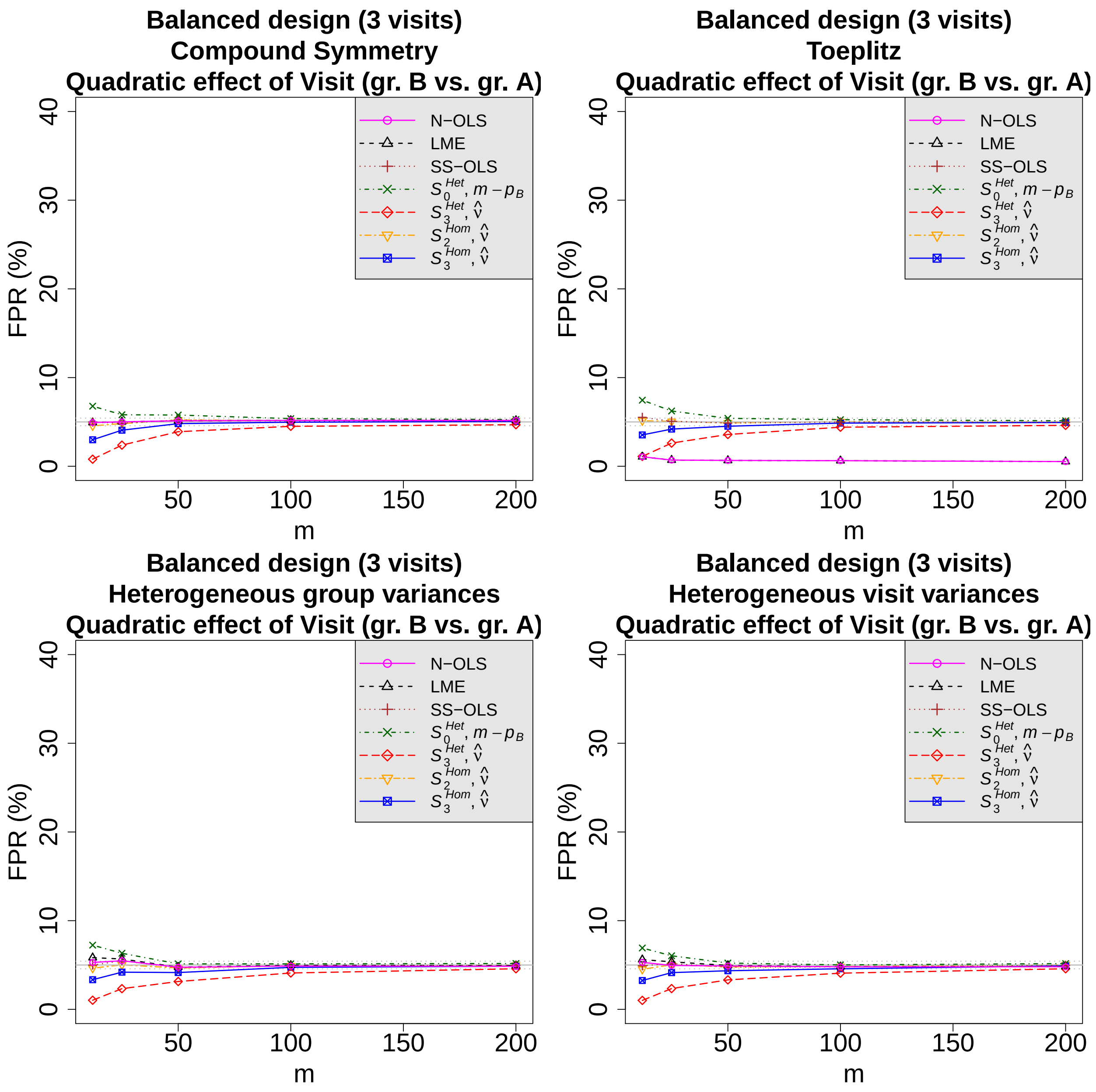

Supplementary Figure 25: FPR comparison on the quadratic effect of visit difference between group B and group A with Compound Symmetry (top left, $\rho = 0.95$), Toeplitz (top right, $\psi = 0.1$ per visit), heterogeneous group variances (bottom left, $\alpha_A = 1$ and $\alpha_B = 2$) and heterogeneous visit variances (bottom right, $\gamma = 1$ per visit) for the balanced design with 3 visits per subject; all results are based on an F-test at nominal level 5%; see Supplementary Figure 1 for a description of the SwE versions.

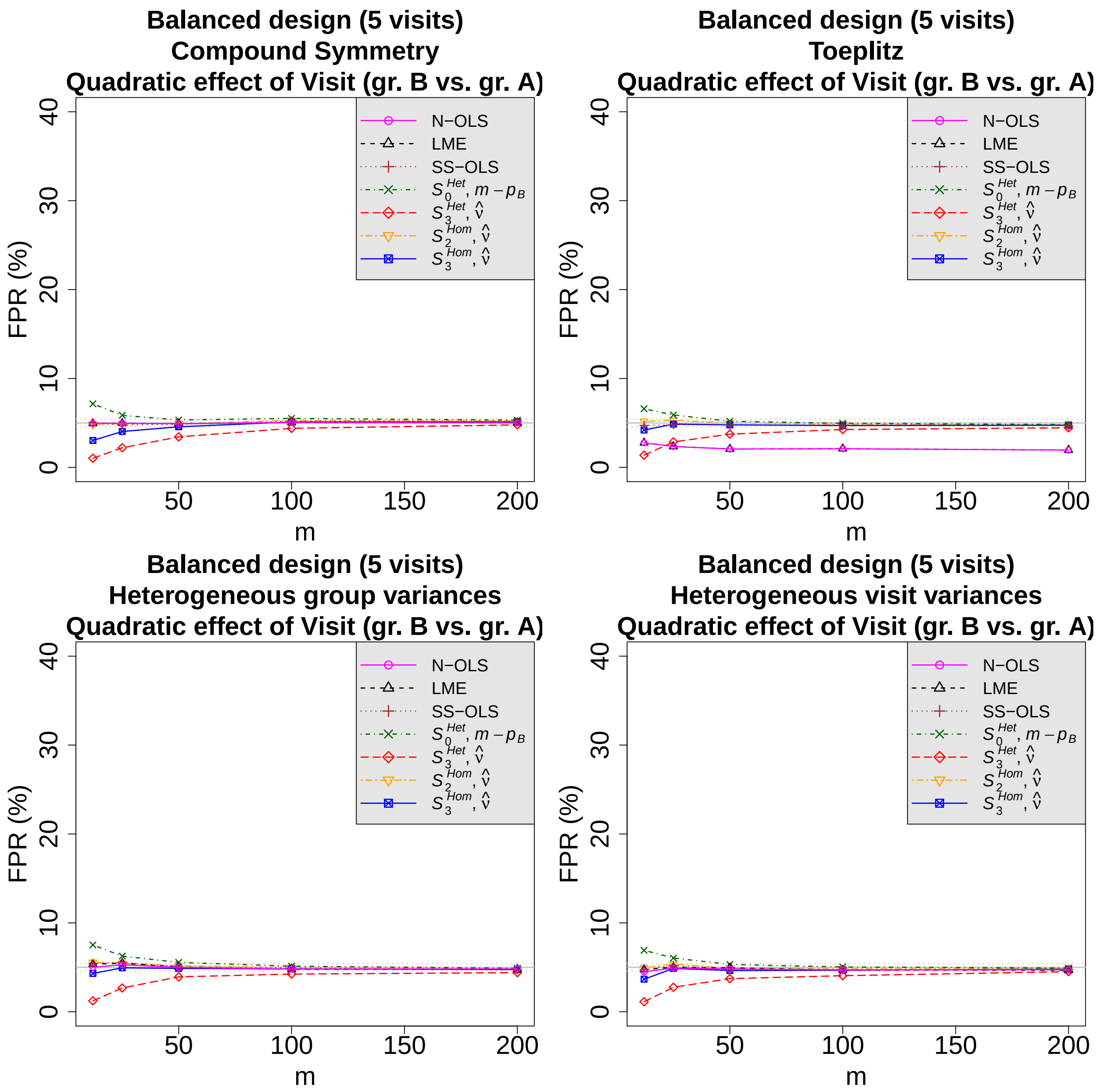

Supplementary Figure 26: FPR comparison on the quadratic effect of visit difference between group B and group A with Compound Symmetry (top left, $\rho = 0.95$), Toeplitz (top right, $\psi = 0.1$ per visit), heterogeneous group variances (bottom left, $\alpha_{\mathrm{A}} = 1$ and $\alpha_{\mathrm{B}} = 2$) and heterogeneous visit variances (bottom right, $\gamma = 1$ per visit) for the balanced design with 5 visits per subject; all results are based on an F-test at nominal level 5%; see Supplementary Figure 1 for a description of the SwE versions.

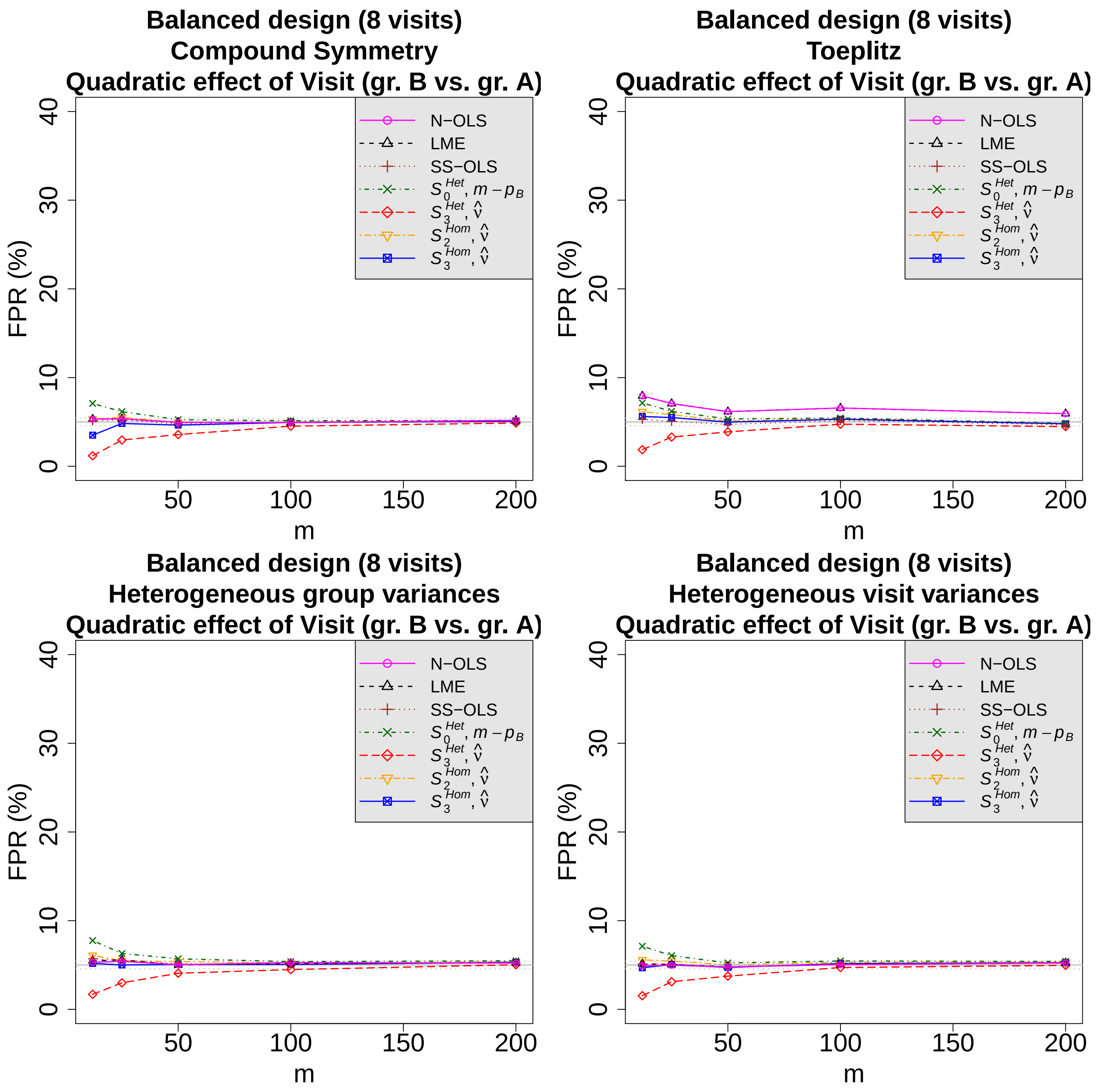

Supplementary Figure 27: FPR comparison on the quadratic effect of visit difference between group B and group A with Compound Symmetry (top left, $\rho = 0.95$), Toeplitz (top right, $\psi = 0.1$ per visit), heterogeneous group variances (bottom left, $\alpha_A = 1$ and $\alpha_B = 2$) and heterogeneous visit variances (bottom right, $\gamma = 1$ per visit) for the balanced design with 8 visits per subject; all results are based on an F-test at nominal level 5%; see Supplementary Figure 1 for a description of the SwE versions.

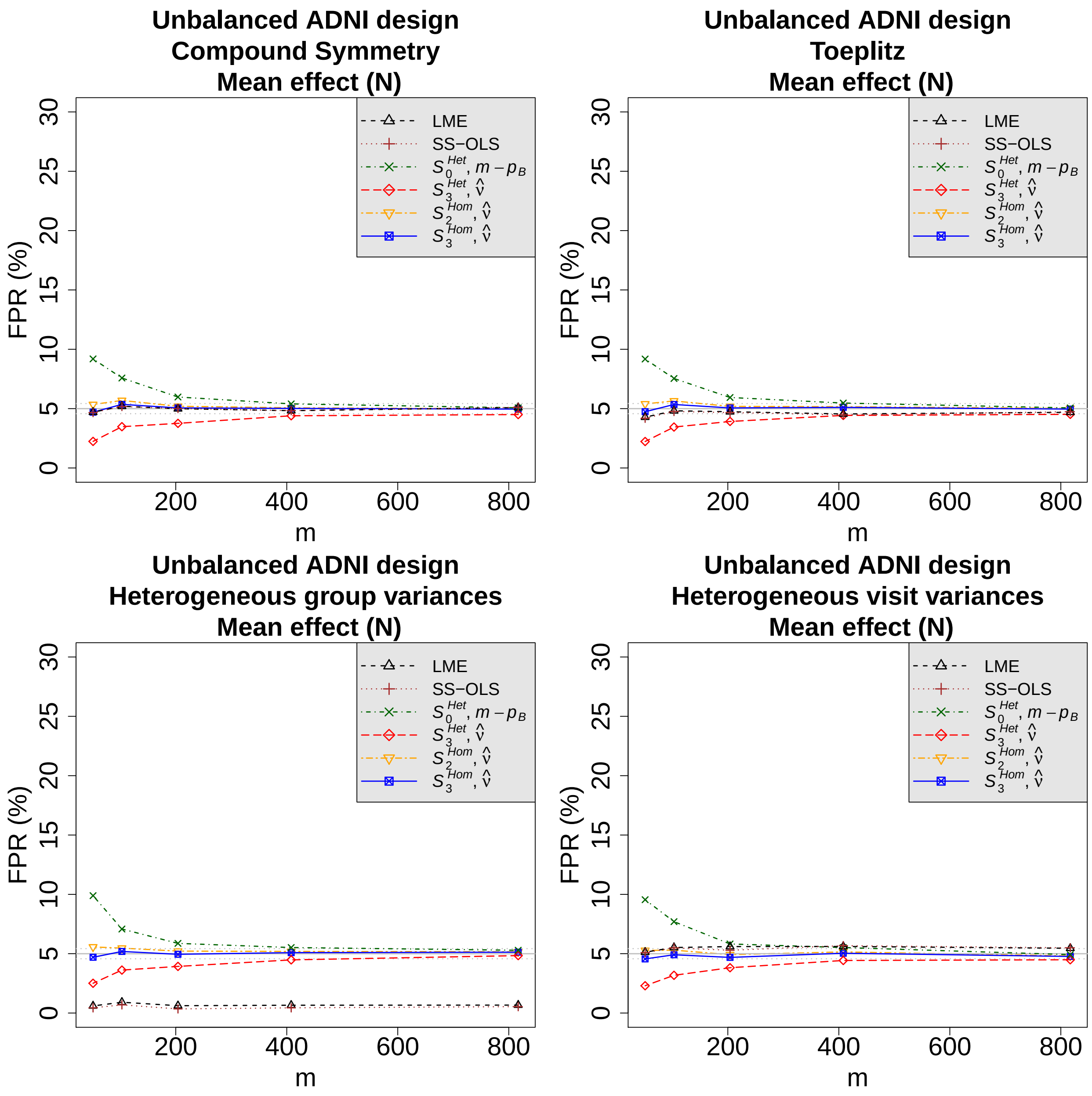

Supplementary Figure 28: FPR comparison on the mean effect of the Normal subjects with Compound Symmetry (top left, $\rho = 0.95$), Toeplitz (top right, $\psi = 0.2$ per year), heterogeneous group variances (bottom left, $\alpha_{\mathrm{N}} = 1$, $\alpha_{\mathrm{MCI}} = 2$ and $\alpha_{\mathrm{AD}} = 3$) and heterogeneous visit variances (bottom right, $\gamma = 2$ per year) for the unbalanced ADNI design; all results are based on an F-test at nominal level 5%; see Supplementary Figure 1 for a description of the SwE versions.

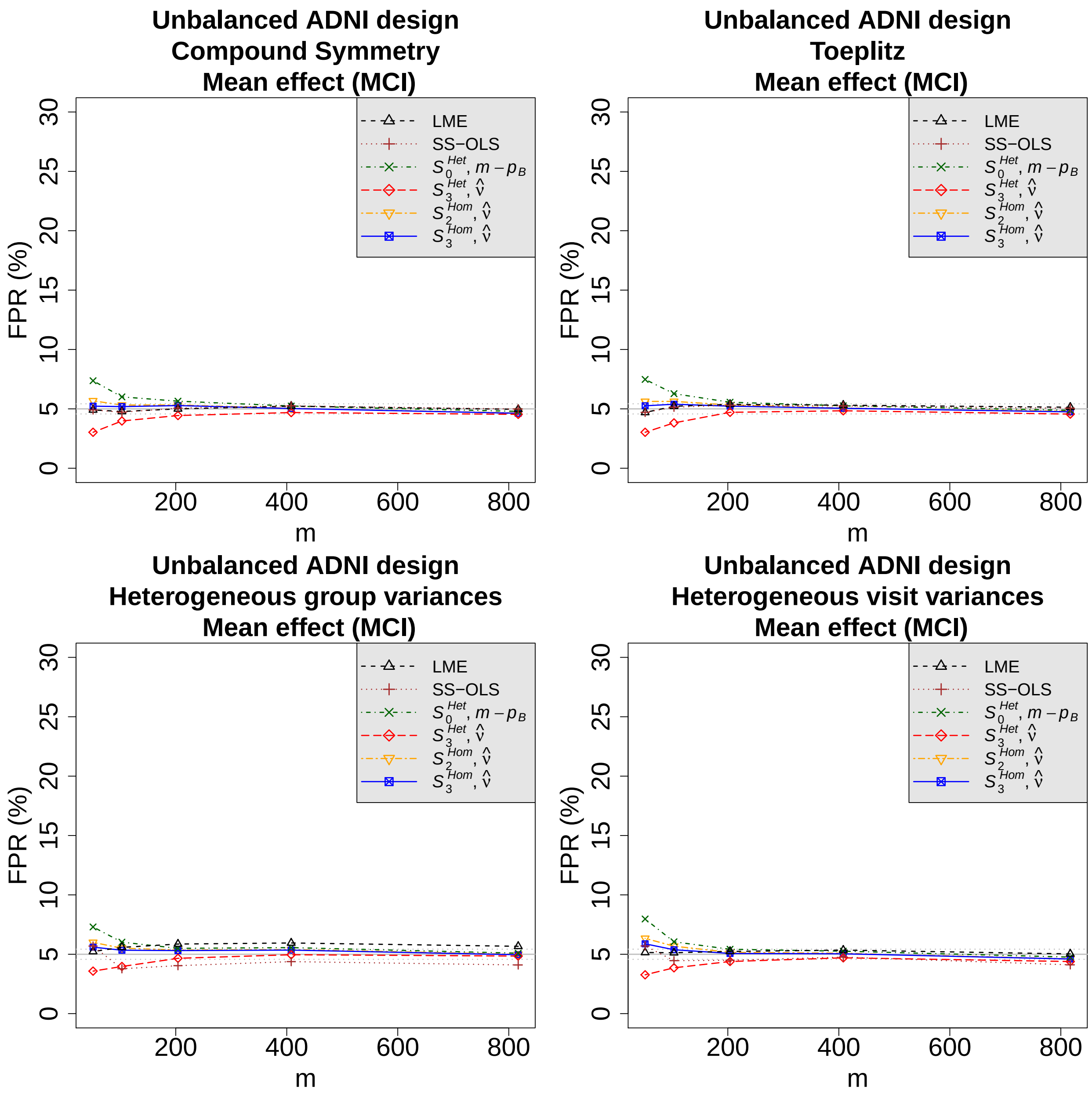

Supplementary Figure 29: FPR comparison on the mean effect of the MCI subjects with Compound Symmetry (top left, $\rho = 0.95$), Toeplitz (top right, $\psi = 0.2$ per year), heterogeneous group variances (bottom left, $\alpha_{\mathrm{N}} = 1$, $\alpha_{\mathrm{MCI}} = 2$ and $\alpha_{\mathrm{AD}} = 3$) and heterogeneous visit variances (bottom right, $\gamma = 2$ per year) for the unbalanced ADNI design; all results are based on an F-test at nominal level 5%; see Supplementary Figure 1 for a description of the SwE versions.

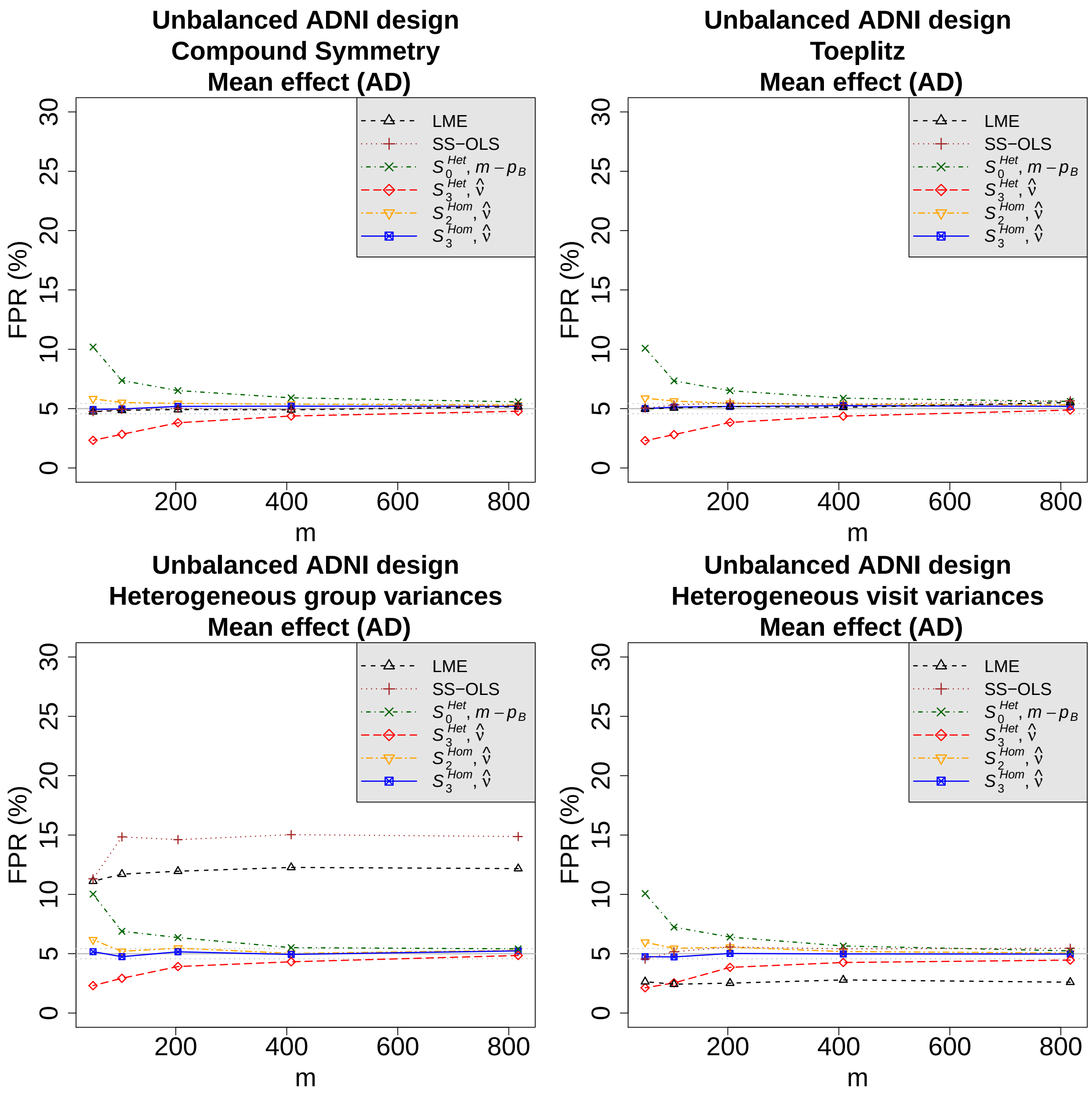

Supplementary Figure 30: FPR comparison on the mean effect of the AD subjects with Compound Symmetry (top left, $\rho = 0.95$), Toeplitz (top right, $\psi = 0.2$ per year), heterogeneous group variances (bottom left, $\alpha_{\mathrm{N}} = 1$, $\alpha_{\mathrm{MCI}} = 2$ and $\alpha_{\mathrm{AD}} = 3$) and heterogeneous visit variances (bottom right, $\gamma = 2$ per year) for the unbalanced ADNI design; all results are based on an F-test at nominal level 5%; see Supplementary Figure 1 for a description of the SwE versions.

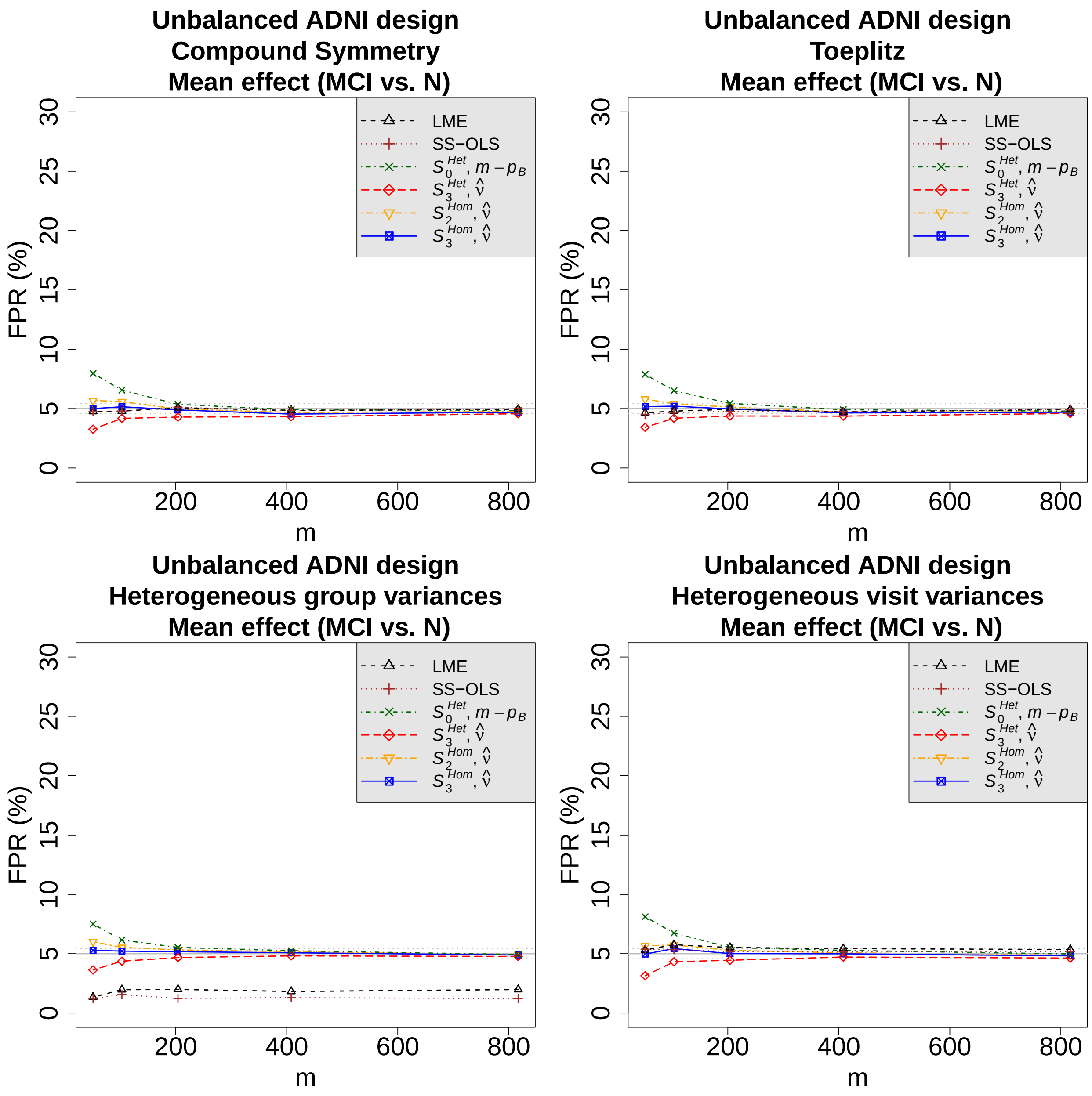

Supplementary Figure 31: FPR comparison on the mean effect difference between the MCI and Normal subjects with Compound Symmetry (top left, $\rho = 0.95$), Toeplitz (top right, $\psi = 0.2$ per year), heterogeneous group variances (bottom left, $\alpha_{\mathrm{N}} = 1$, $\alpha_{\mathrm{MCI}} = 2$ and $\alpha_{\mathrm{AD}} = 3$) and heterogeneous visit variances (bottom right, $\gamma = 2$ per year) for the unbalanced ADNI design; all results are based on an F-test at nominal level 5%; see Supplementary Figure 1 for a description of the SwE versions.

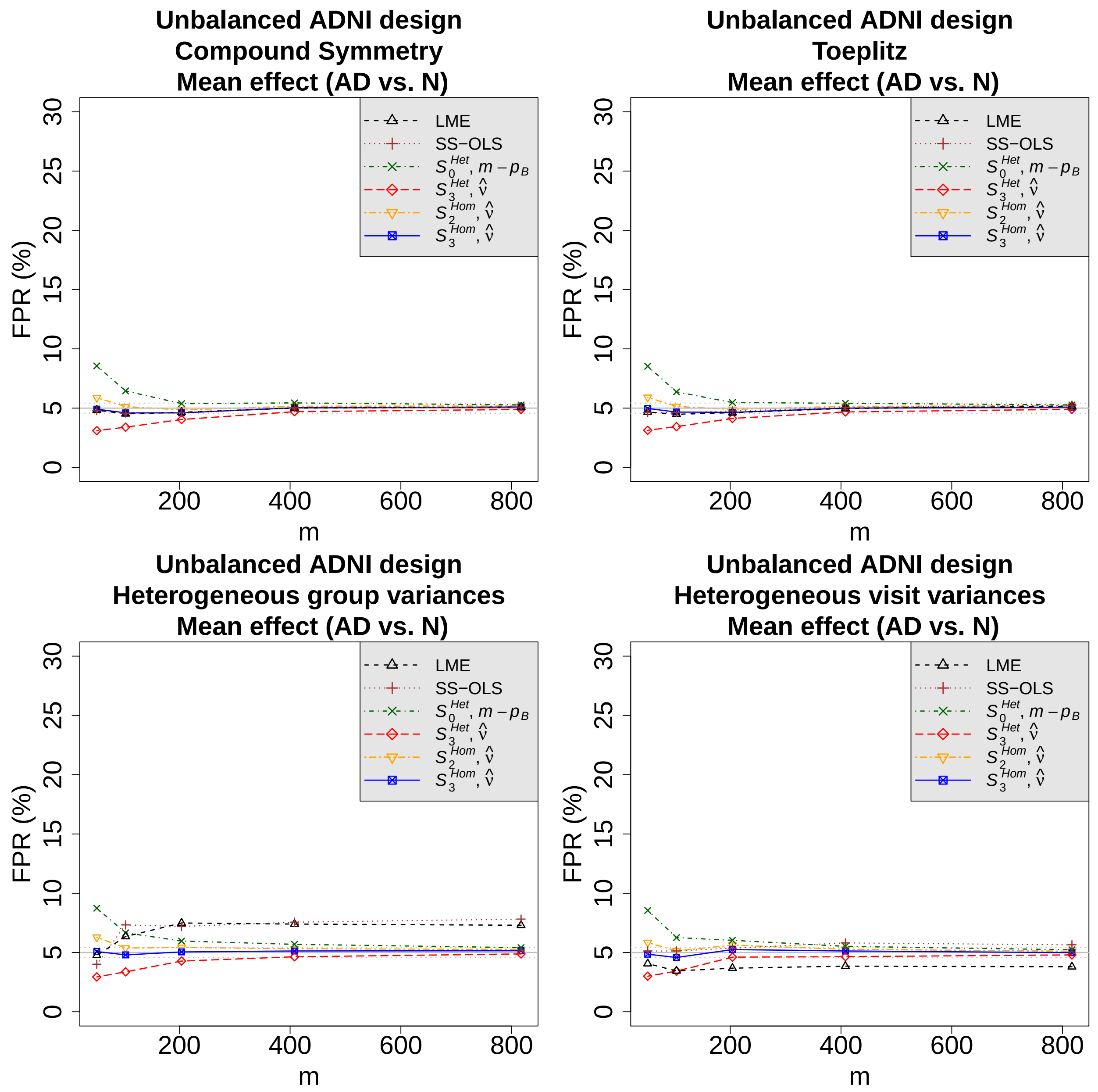

Supplementary Figure 32: FPR comparison on the mean effect difference between the AD and Normal subjects with Compound Symmetry (top left, $\rho = 0.95$), Toeplitz (top right, $\psi = 0.2$ per year), heterogeneous group variances (bottom left, $\alpha_{\mathrm{N}} = 1$, $\alpha_{\mathrm{MCI}} = 2$ and $\alpha_{\mathrm{AD}} = 3$) and heterogeneous visit variances (bottom right, $\gamma = 2$ per year) for the unbalanced ADNI design; all results are based on an F-test at nominal level 5%; see Supplementary Figure 1 for a description of the SwE versions.

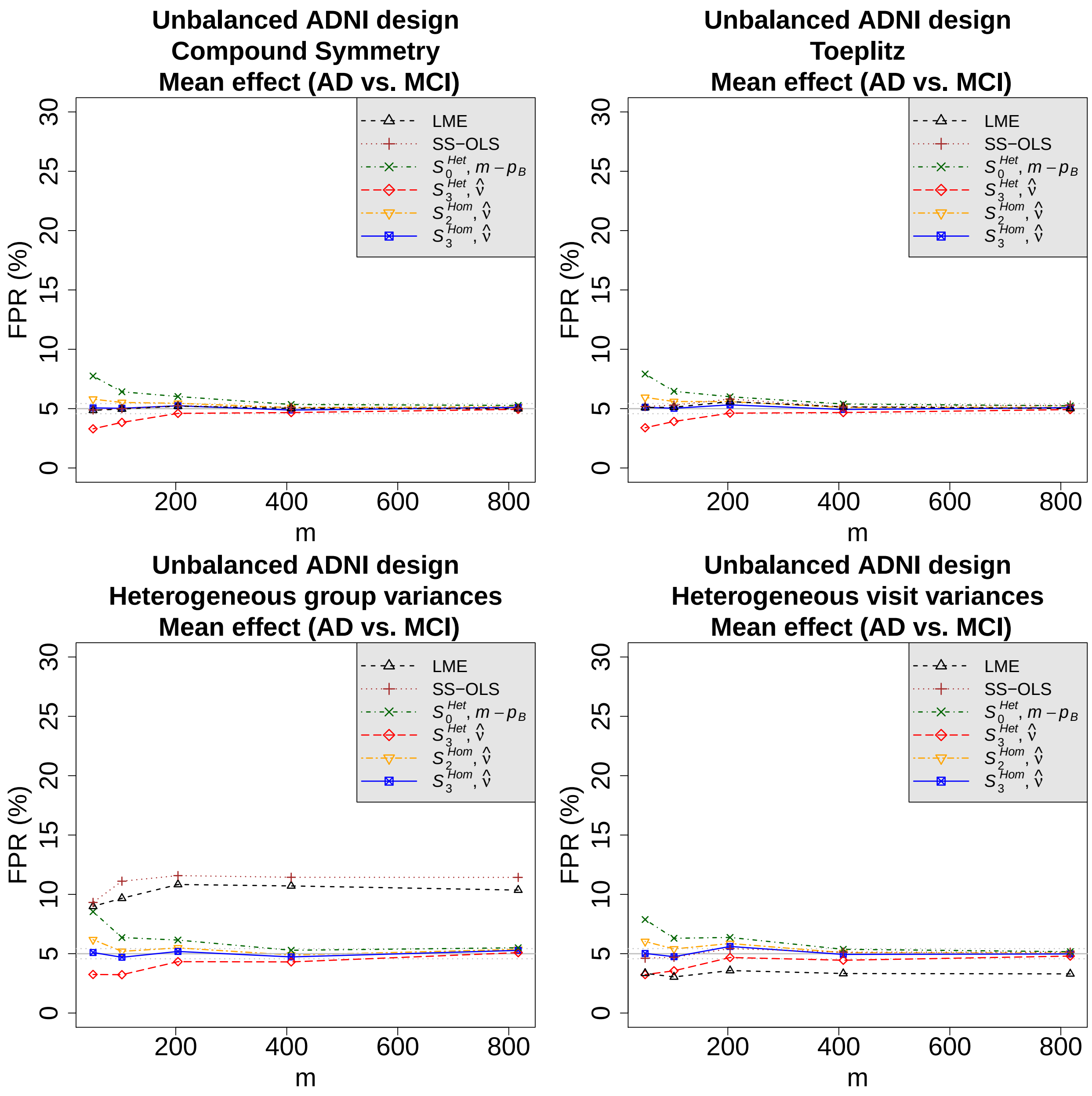

Supplementary Figure 33: FPR comparison on the mean effect difference between the AD and MCI subjects with Compound Symmetry (top left, $\rho = 0.95$), Toeplitz (top right, $\psi = 0.2$ per year), heterogeneous group variances (bottom left, $\alpha_{\mathrm{N}} = 1$, $\alpha_{\mathrm{MCI}} = 2$ and $\alpha_{\mathrm{AD}} = 3$) and heterogeneous visit variances (bottom right, $\gamma = 2$ per year) for the unbalanced ADNI design; all results are based on an F-test at nominal level 5%; see Supplementary Figure 1 for a description of the SwE versions.

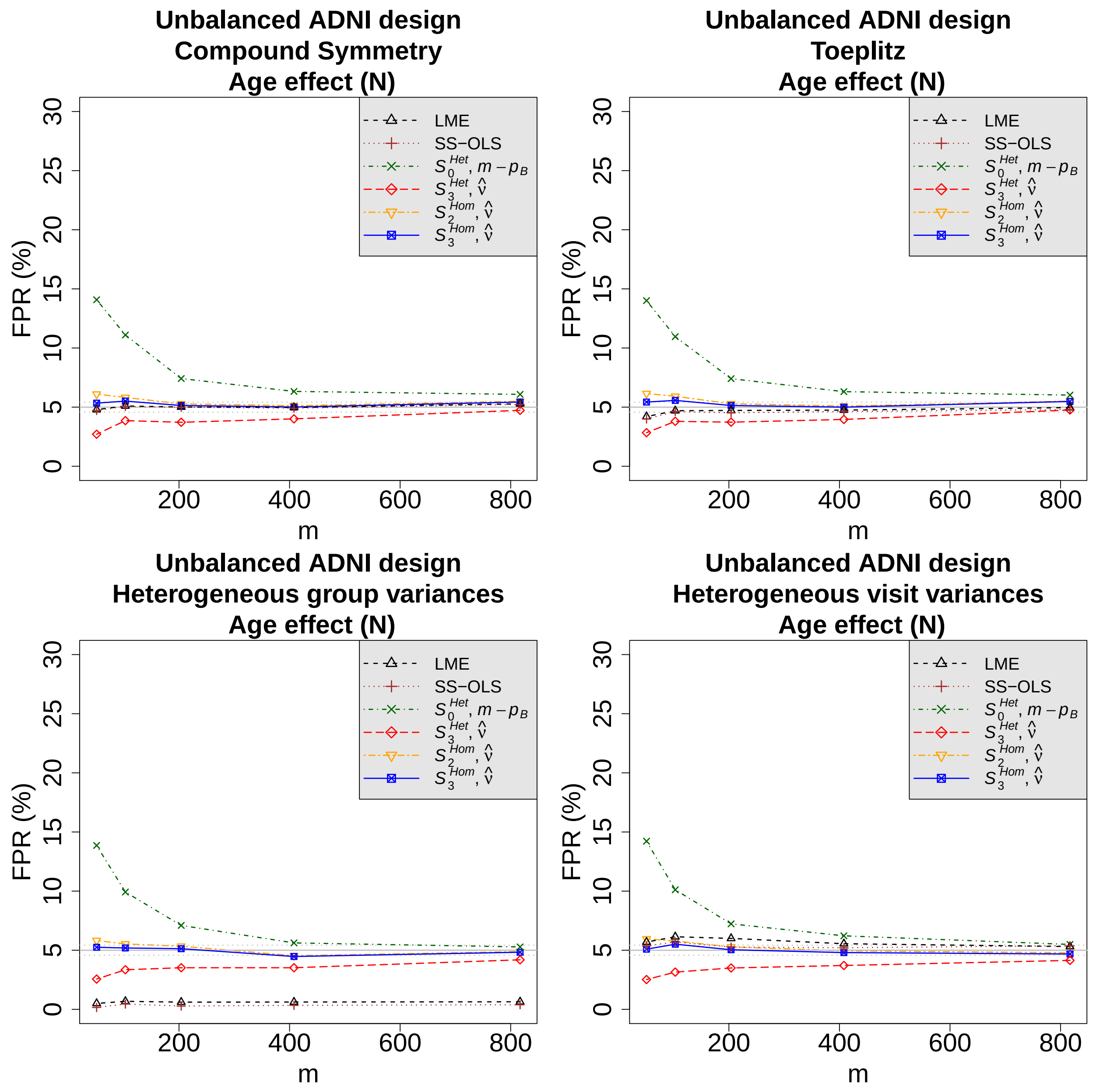

Supplementary Figure 34: FPR comparison on the age effect of the Normal subjects with Compound Symmetry (top left, $\rho = 0.95$), Toeplitz (top right, $\psi = 0.2$ per year), heterogeneous group variances (bottom left, $\alpha_{\mathrm{N}} = 1$, $\alpha_{\mathrm{MCI}} = 2$ and $\alpha_{\mathrm{AD}} = 3$) and heterogeneous visit variances (bottom right, $\gamma = 2$ per year) for the unbalanced ADNI design; all results are based on an F-test at nominal level 5%; see Supplementary Figure 1 for a description of the SwE versions.

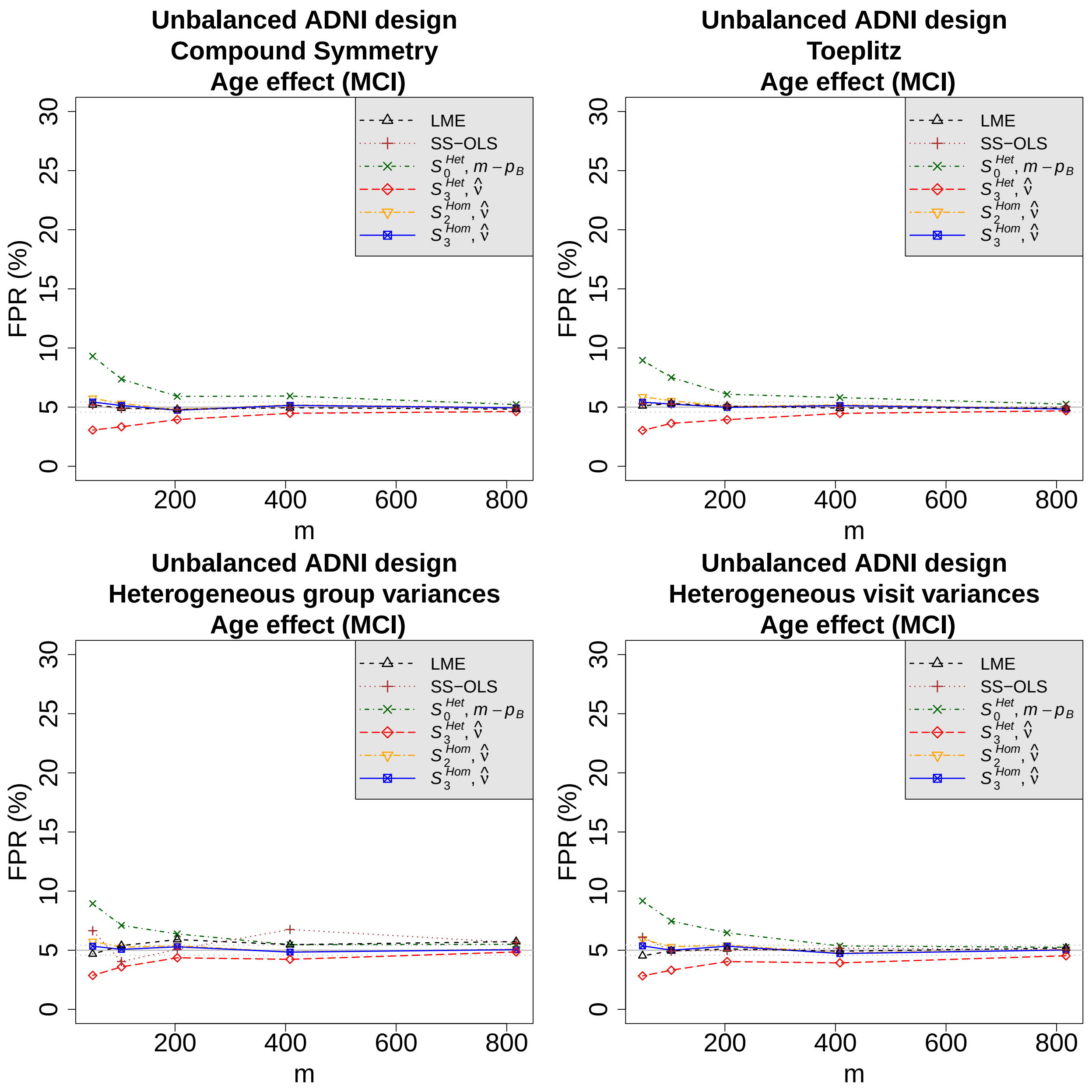

Supplementary Figure 35: FPR comparison on the age effect of the MCI subjects with Compound Symmetry (top left, $\rho = 0.95$), Toeplitz (top right, $\psi = 0.2$ per year), heterogeneous group variances (bottom left, $\alpha_{\text{N}} = 1$, $\alpha_{\text{MCI}} = 2$ and $\alpha_{\text{AD}} = 3$) and heterogeneous visit variances (bottom right, $\gamma = 2$ per year) for the unbalanced ADNI design; all results are based on an F-test at nominal level 5%; see Supplementary Figure 1 for a description of the SwE versions.

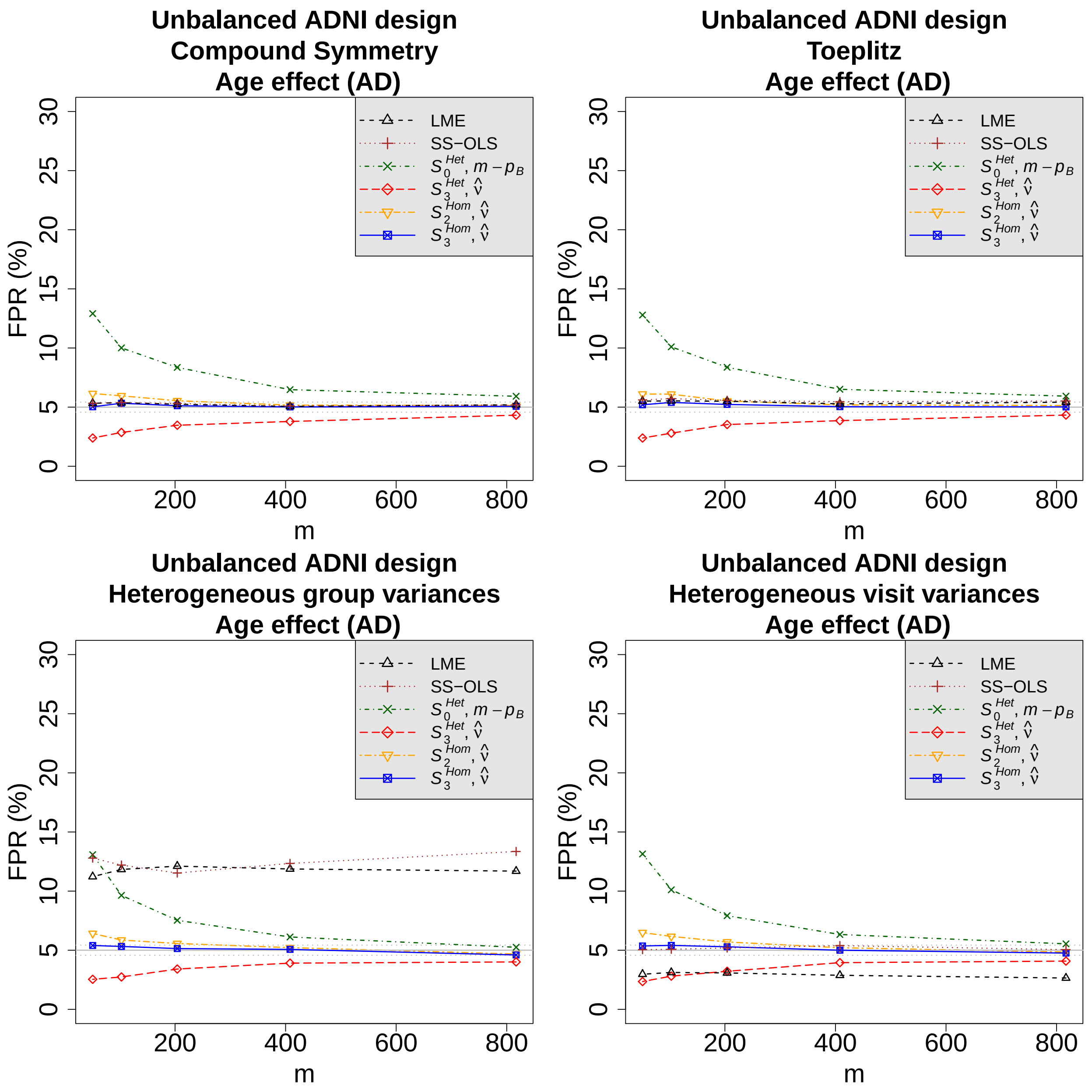

Supplementary Figure 36: FPR comparison on the age effect of the AD subjects with Compound Symmetry (top left, $\rho = 0.95$), Toeplitz (top right, $\psi = 0.2$ per year), heterogeneous group variances (bottom left, $\alpha_{\mathrm{N}} = 1$, $\alpha_{\mathrm{MCI}} = 2$ and $\alpha_{\mathrm{AD}} = 3$) and heterogeneous visit variances (bottom right, $\gamma = 2$ per year) for the unbalanced ADNI design; all results are based on an F-test at nominal level 5%; see Supplementary Figure 1 for a description of the SwE versions.

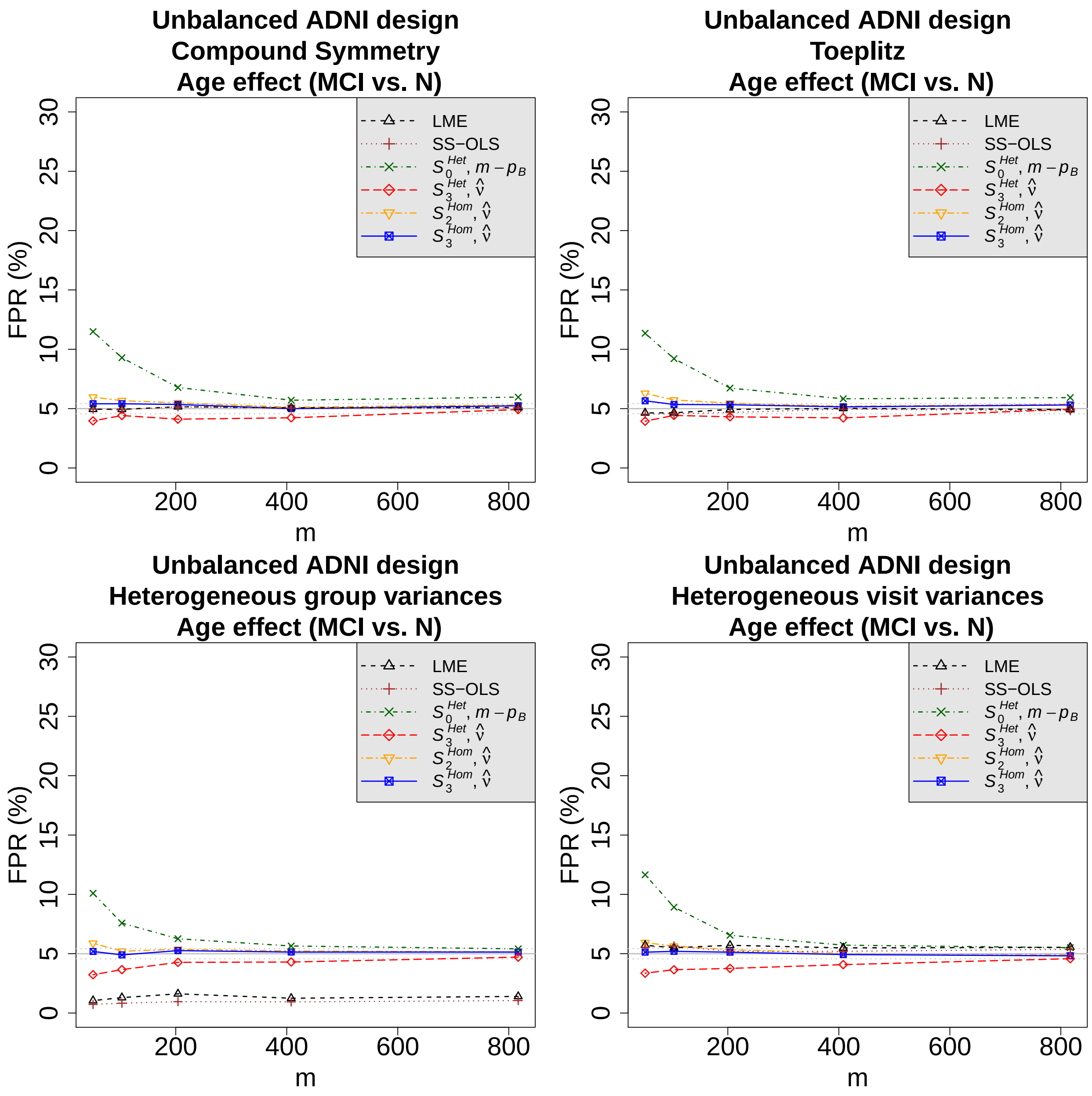

Supplementary Figure 37: FPR comparison on the age effect difference between the MCI and Normal subjects with Compound Symmetry (top left, $\rho = 0.95$), Toeplitz (top right, $\psi = 0.2$ per year), heterogeneous group variances (bottom left, $\alpha_{\mathrm{N}} = 1$, $\alpha_{\mathrm{MCI}} = 2$ and $\alpha_{\mathrm{AD}} = 3$) and heterogeneous visit variances (bottom right, $\gamma = 2$ per year) for the unbalanced ADNI design; all results are based on an F-test at nominal level 5%; see Supplementary Figure 1 for a description of the SwE versions.

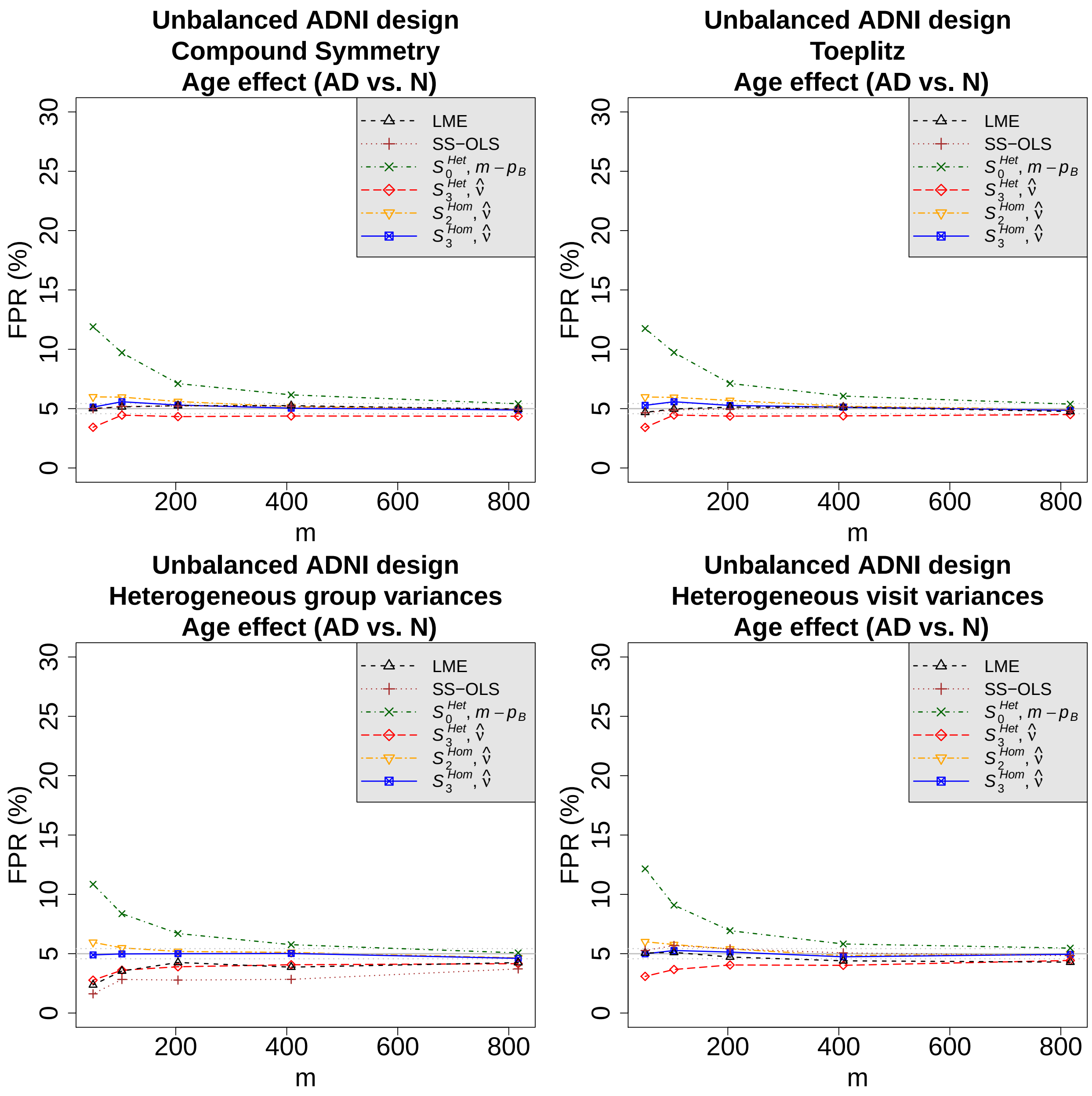

Supplementary Figure 38: FPR comparison on the age effect difference between the AD and Normal subjects with Compound Symmetry (top left, $\rho = 0.95$), Toeplitz (top right, $\psi = 0.2$ per year), heterogeneous group variances (bottom left, $\alpha_{\mathrm{N}} = 1$, $\alpha_{\mathrm{MCI}} = 2$ and $\alpha_{\mathrm{AD}} = 3$) and heterogeneous visit variances (bottom right, $\gamma = 2$ per year) for the unbalanced ADNI design; all results are based on an F-test at nominal level 5%; see Supplementary Figure 1 for a description of the SwE versions.

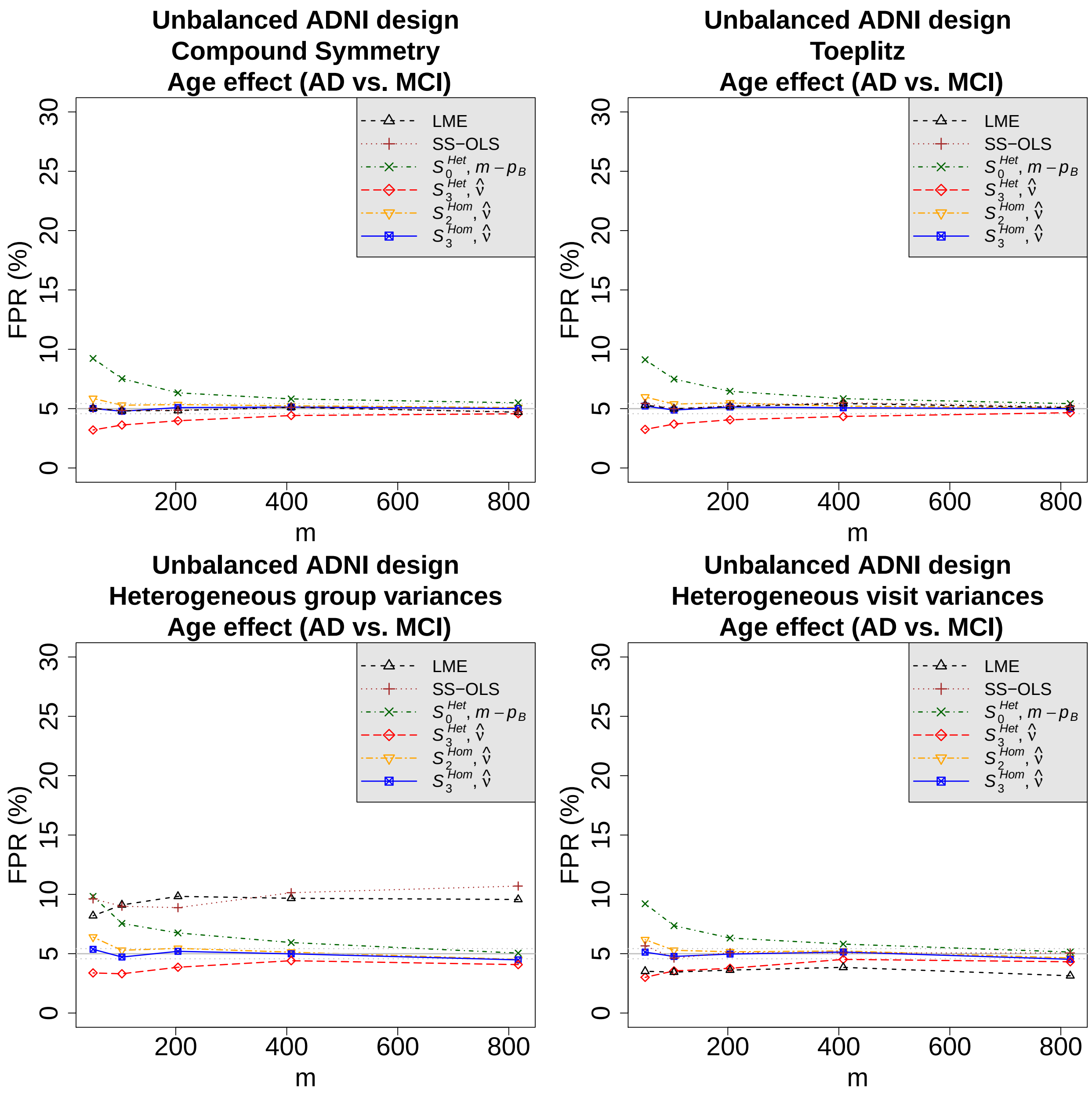

Supplementary Figure 39: FPR comparison on the age effect difference between the AD and MCI subjects with Compound Symmetry (top left, $\rho = 0.95$), Toeplitz (top right, $\psi = 0.2$ per year), heterogeneous group variances (bottom left, $\alpha_{\mathrm{N}} = 1$, $\alpha_{\mathrm{MCI}} = 2$ and $\alpha_{\mathrm{AD}} = 3$) and heterogeneous visit variances (bottom right, $\gamma = 2$ per year) for the unbalanced ADNI design; all results are based on an F-test at nominal level 5%; see Supplementary Figure 1 for a description of the SwE versions.

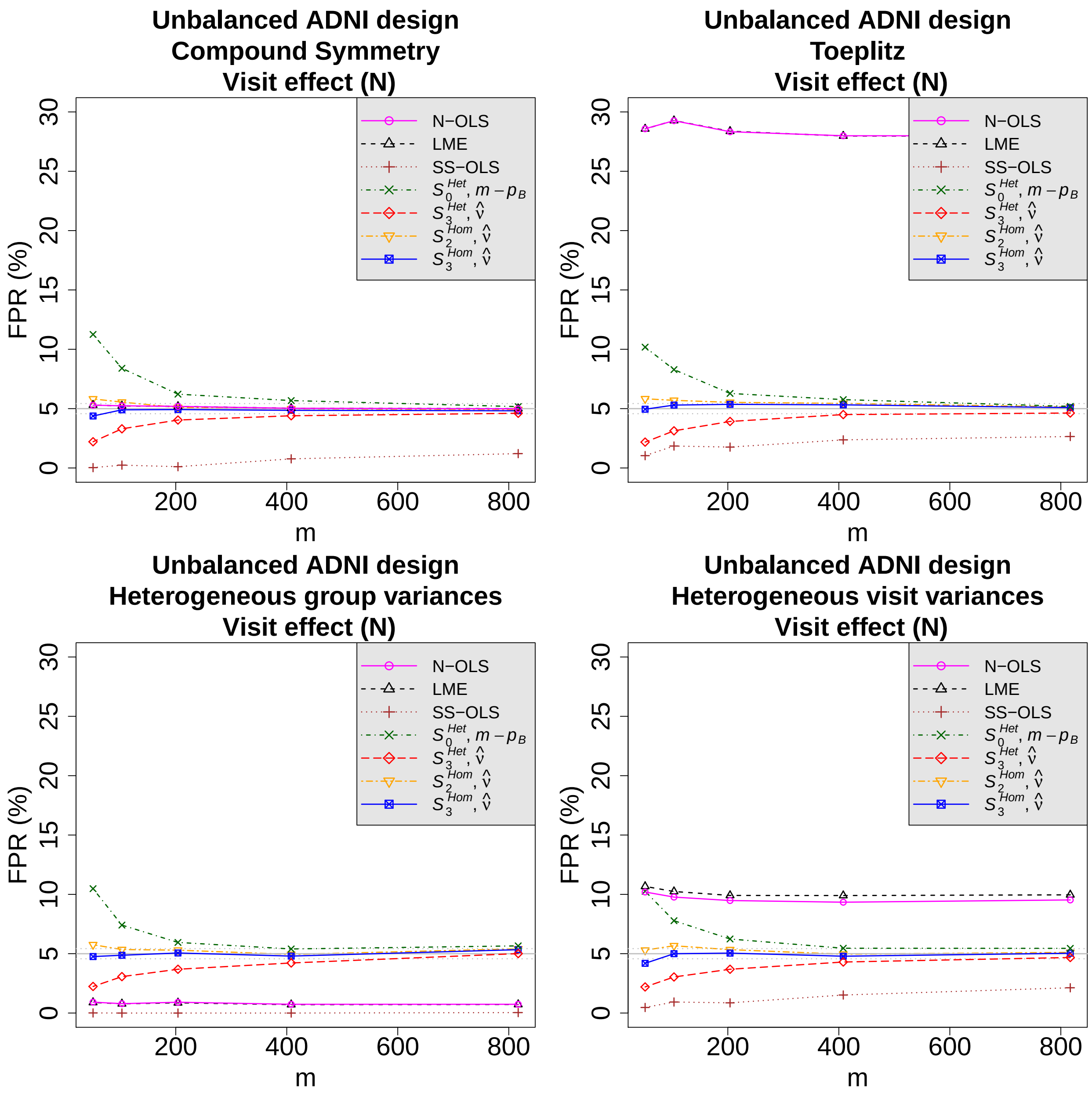

Supplementary Figure 40: FPR comparison on the visit effect of the Normal subjects with Compound Symmetry (top left, $\rho = 0.95$), Toeplitz (top right, $\psi = 0.2$ per year), heterogeneous group variances (bottom left, $\alpha_{\text{N}} = 1$, $\alpha_{\text{MCI}} = 2$ and $\alpha_{\text{AD}} = 3$) and heterogeneous visit variances (bottom right, $\gamma = 2$ per year) for the unbalanced ADNI design; all results are based on an F-test at nominal level 5%; see Supplementary Figure 1 for a description of the SwE versions.

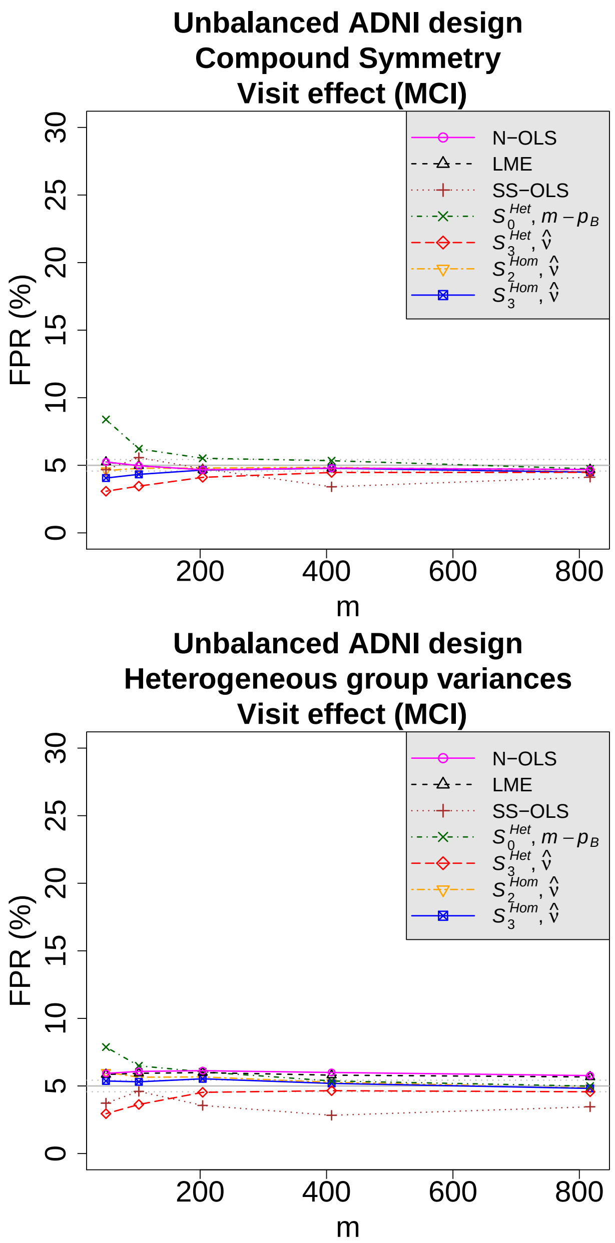

Supplementary Figure 41: FPR comparison on the visit effect of the MCI subjects with Compound Symmetry (top left, $\rho = 0.95$), Toeplitz (top right, $\psi = 0.2$ per year), heterogeneous group variances (bottom left, $\alpha_{\mathrm{N}} = 1$, $\alpha_{\mathrm{MCI}} = 2$ and $\alpha_{\mathrm{AD}} = 3$) and heterogeneous visit variances (bottom right, $\gamma = 2$ per year) for the unbalanced ADNI design; all results are based on an F-test at nominal level 5%; see Supplementary Figure 1 for a description of the SwE versions.

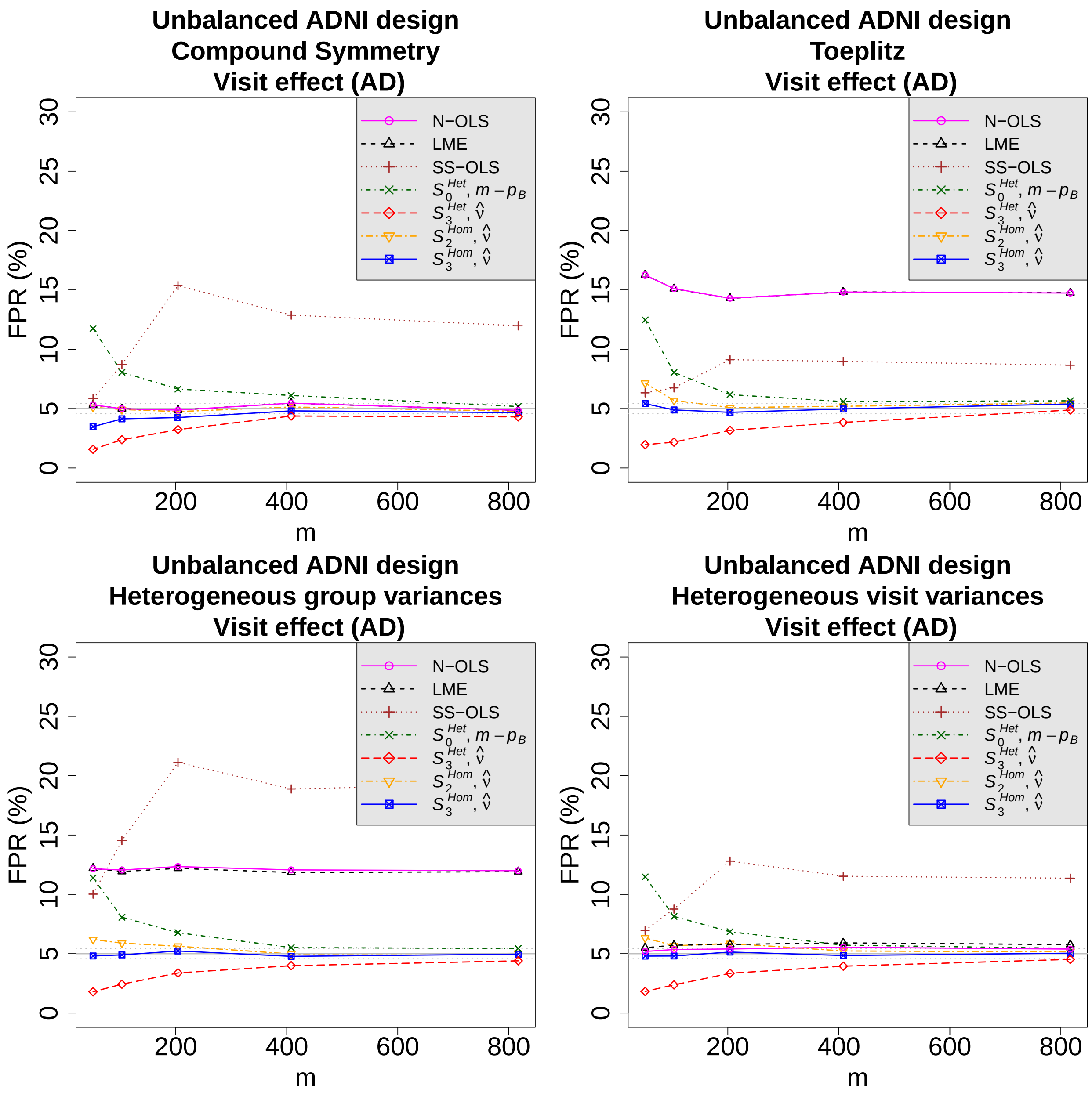

Supplementary Figure 42: FPR comparison on the visit effect of the AD subjects with Compound Symmetry (top left, $\rho = 0.95$), Toeplitz (top right, $\psi = 0.2$ per year), heterogeneous group variances (bottom left, $\alpha_{\mathrm{N}} = 1$, $\alpha_{\mathrm{MCI}} = 2$ and $\alpha_{\mathrm{AD}} = 3$) and heterogeneous visit variances (bottom right, $\gamma = 2$ per year) for the unbalanced ADNI design; all results are based on an F-test at nominal level 5%; see Supplementary Figure 1 for a description of the SwE versions.

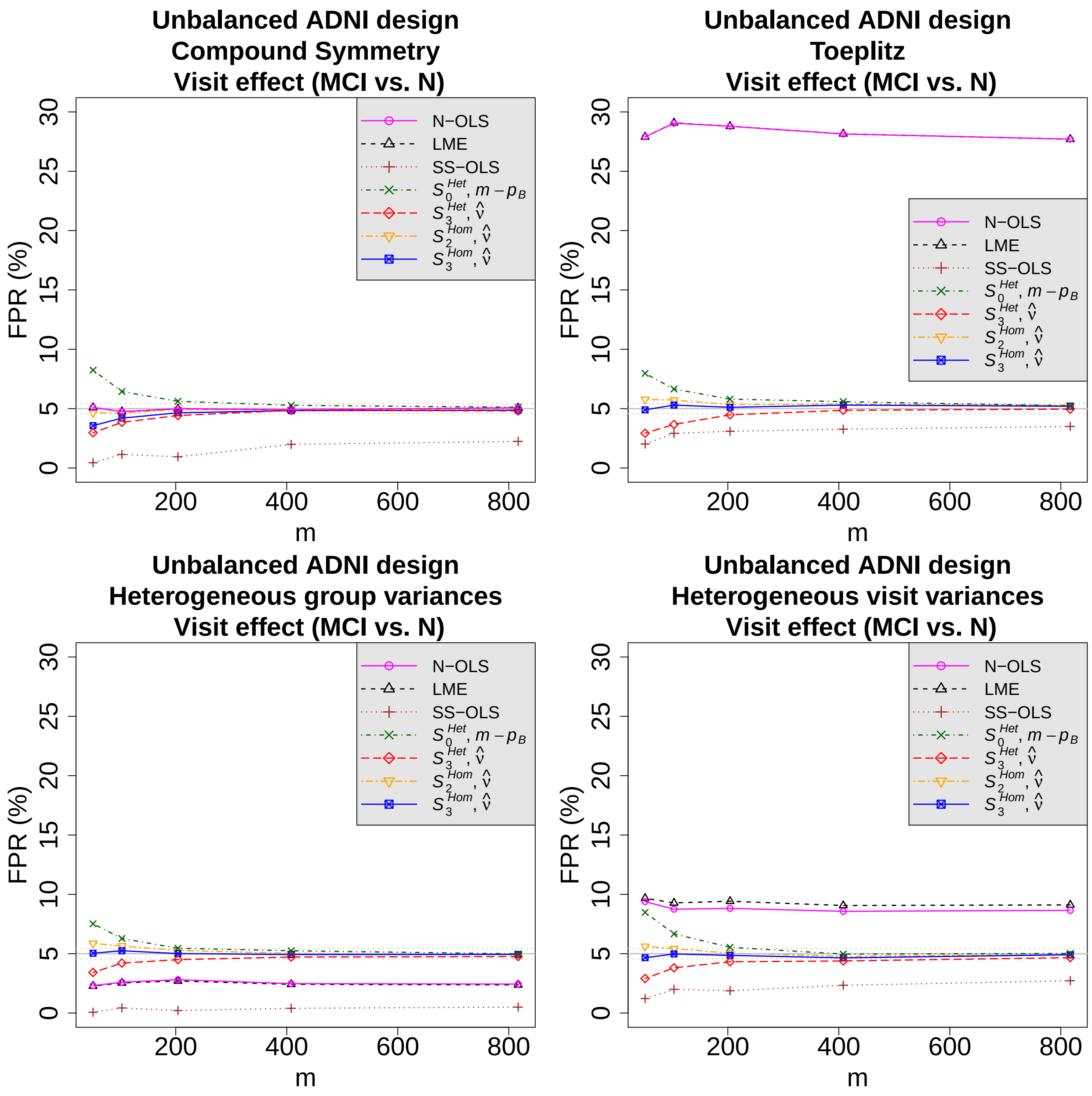

Supplementary Figure 43: FPR comparison on the visit effect difference between the MCI and Normal subjects with Compound Symmetry (top left, $\rho = 0.95$), Toeplitz (top right, $\psi = 0.2$ per year), heterogeneous group variances (bottom left, $\alpha_{\mathrm{N}} = 1$, $\alpha_{\mathrm{MCI}} = 2$ and $\alpha_{\mathrm{AD}} = 3$) and heterogeneous visit variances (bottom right, $\gamma = 2$ per year) for the unbalanced ADNI design; all results are based on an F-test at nominal level 5%; see Supplementary Figure 1 for a description of the SwE versions.

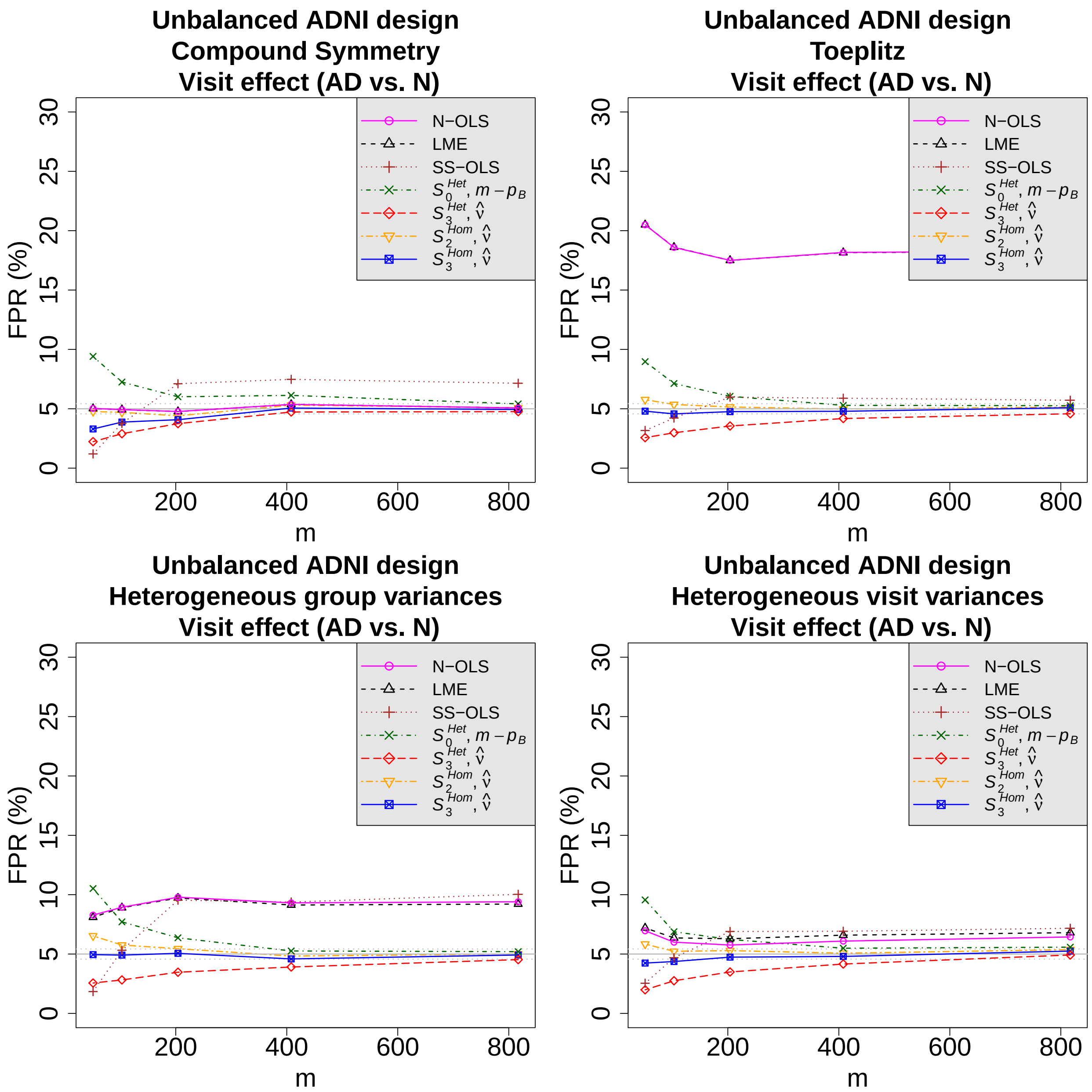

Supplementary Figure 44: FPR comparison on the visit effect difference between the AD and Normal subjects with Compound Symmetry (top left, $\rho = 0.95$), Toeplitz (top right, $\psi = 0.2$ per year), heterogeneous group variances (bottom left, $\alpha_{\mathrm{N}} = 1$, $\alpha_{\mathrm{MCI}} = 2$ and $\alpha_{\mathrm{AD}} = 3$) and heterogeneous visit variances (bottom right, $\gamma = 2$ per year) for the unbalanced ADNI design; all results are based on an F-test at nominal level 5%; see Supplementary Figure 1 for a description of the SwE versions.

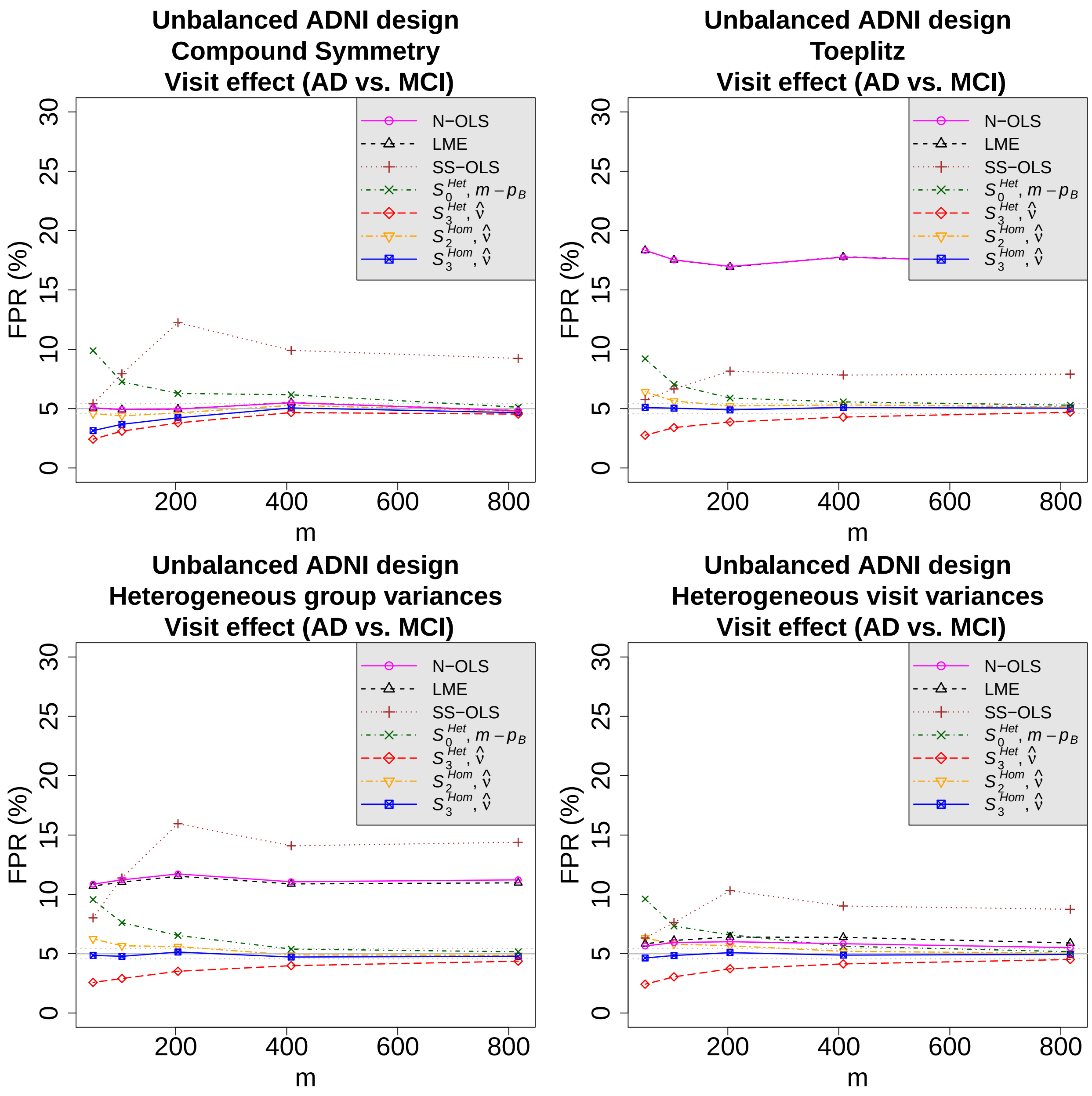

Supplementary Figure 45: FPR comparison on the visit effect difference between the AD and MCI subjects with Compound Symmetry (top left, $\rho = 0.95$), Toeplitz (top right, $\psi = 0.2$ per year), heterogeneous group variances (bottom left, $\alpha_{\mathrm{N}} = 1$, $\alpha_{\mathrm{MCI}} = 2$ and $\alpha_{\mathrm{AD}} = 3$) and heterogeneous visit variances (bottom right, $\gamma = 2$ per year) for the unbalanced ADNI design; all results are based on an F-test at nominal level 5%; see Supplementary Figure 1 for a description of the SwE versions.

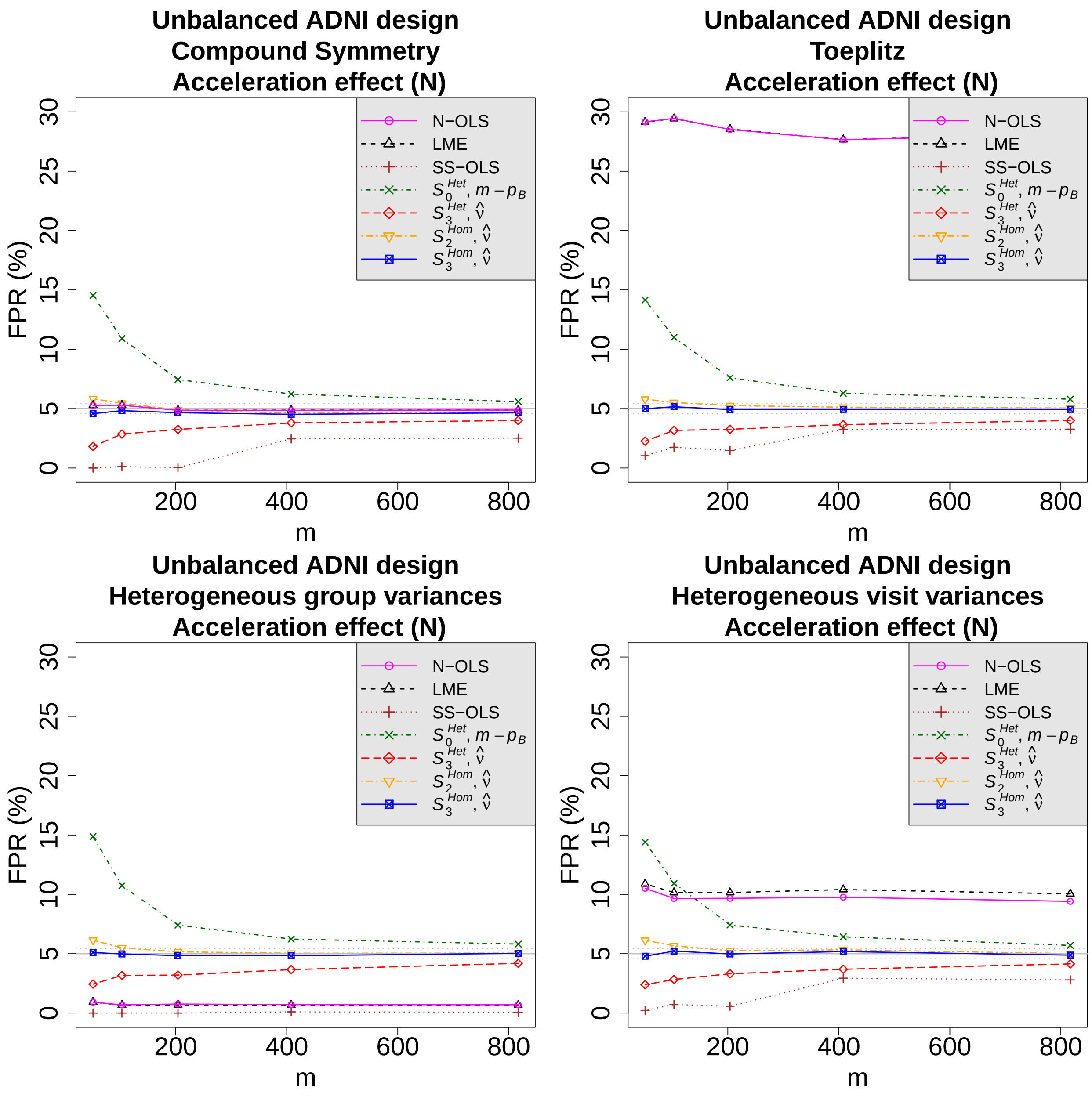

Supplementary Figure 46: FPR comparison on the acceleration effect of the Normal subjects with Compound Symmetry (top left, $\rho = 0.95$), Toeplitz (top right, $\psi = 0.2$ per year), heterogeneous group variances (bottom left, $\alpha_{\mathrm{N}} = 1$, $\alpha_{\mathrm{MCI}} = 2$ and $\alpha_{\mathrm{AD}} = 3$) and heterogeneous visit variances (bottom right, $\gamma = 2$ per year) for the unbalanced ADNI design; all results are based on an F-test at nominal level 5%; see Supplementary Figure 1 for a description of the SwE versions.

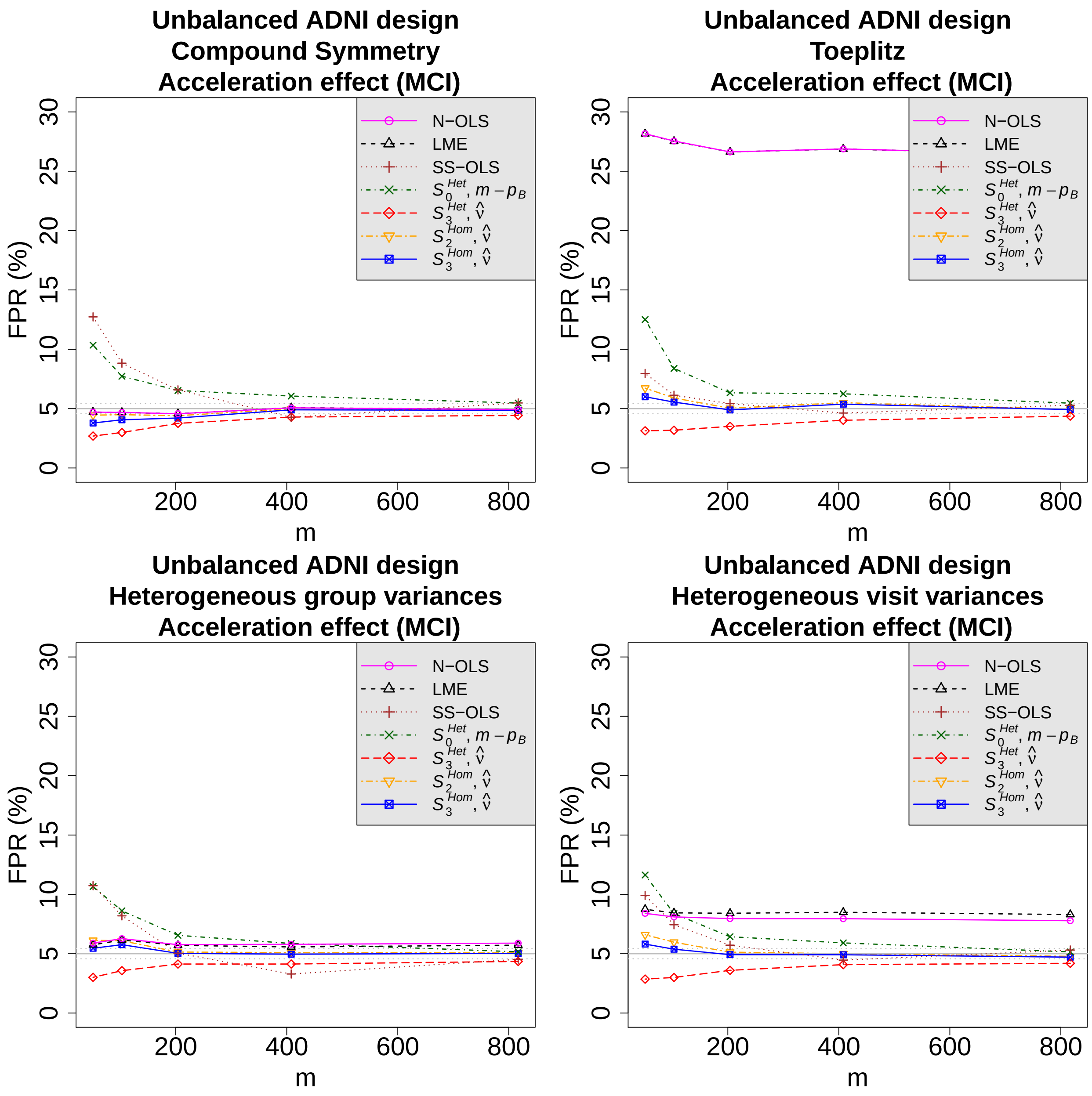

Supplementary Figure 47: FPR comparison on the acceleration effect of the MCI subjects with Compound Symmetry (top left, $\rho = 0.95$), Toeplitz (top right, $\psi = 0.2$ per year), heterogeneous group variances (bottom left, $\alpha_{\mathrm{N}} = 1$, $\alpha_{\mathrm{MCI}} = 2$ and $\alpha_{\mathrm{AD}} = 3$) and heterogeneous visit variances (bottom right, $\gamma = 2$ per year) for the unbalanced ADNI design; all results are based on an F-test at nominal level 5%; see Supplementary Figure 1 for a description of the SwE versions.

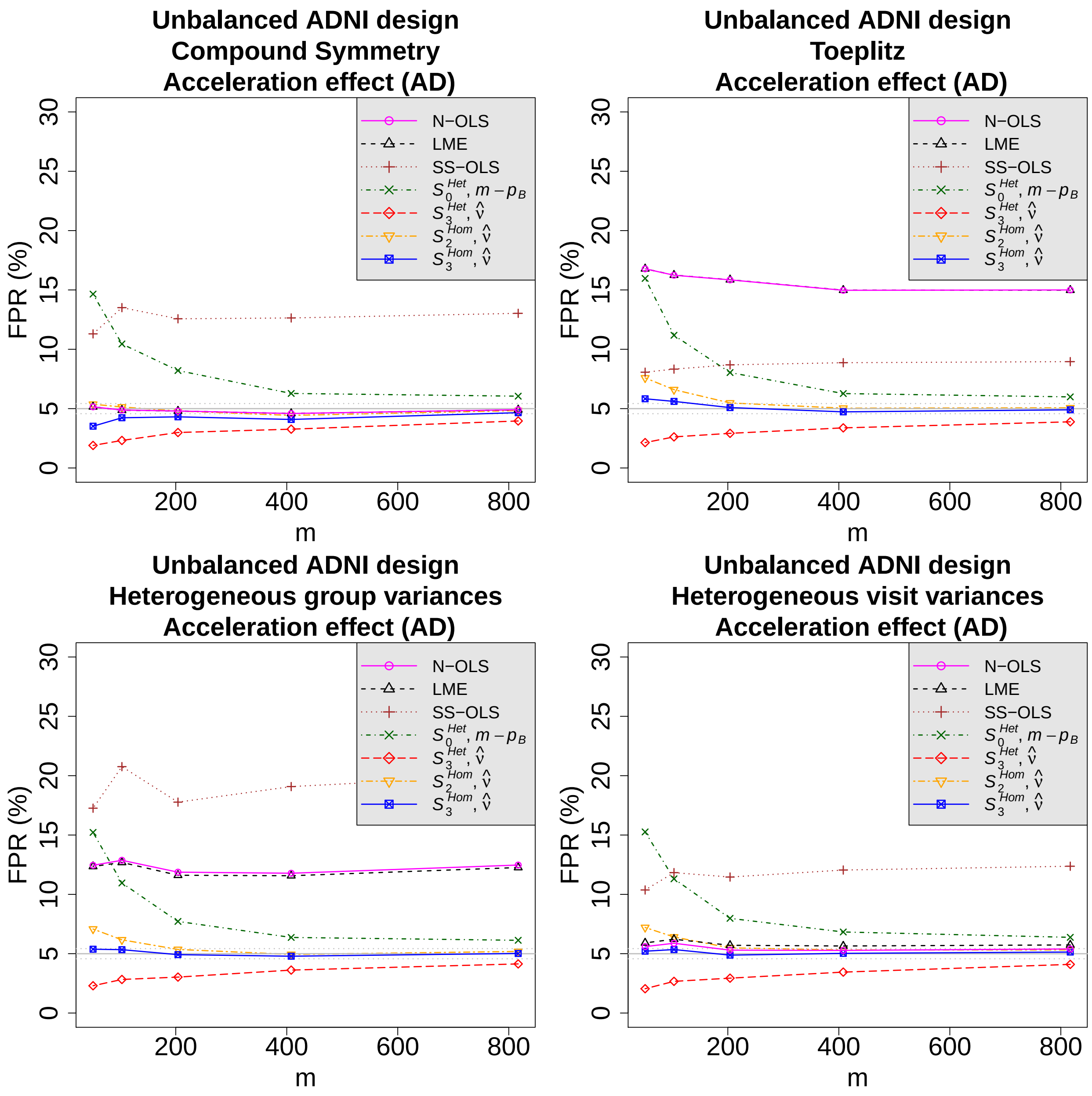

Supplementary Figure 48: FPR comparison on the acceleration effect of the AD subjects with Compound Symmetry (top left, $\rho = 0.95$), Toeplitz (top right, $\psi = 0.2$ per year), heterogeneous group variances (bottom left, $\alpha_{\mathrm{N}} = 1$, $\alpha_{\mathrm{MCI}} = 2$ and $\alpha_{\mathrm{AD}} = 3$) and heterogeneous visit variances (bottom right, $\gamma = 2$ per year) for the unbalanced ADNI design; all results are based on an F-test at nominal level 5%; see Supplementary Figure 1 for a description of the SwE versions.

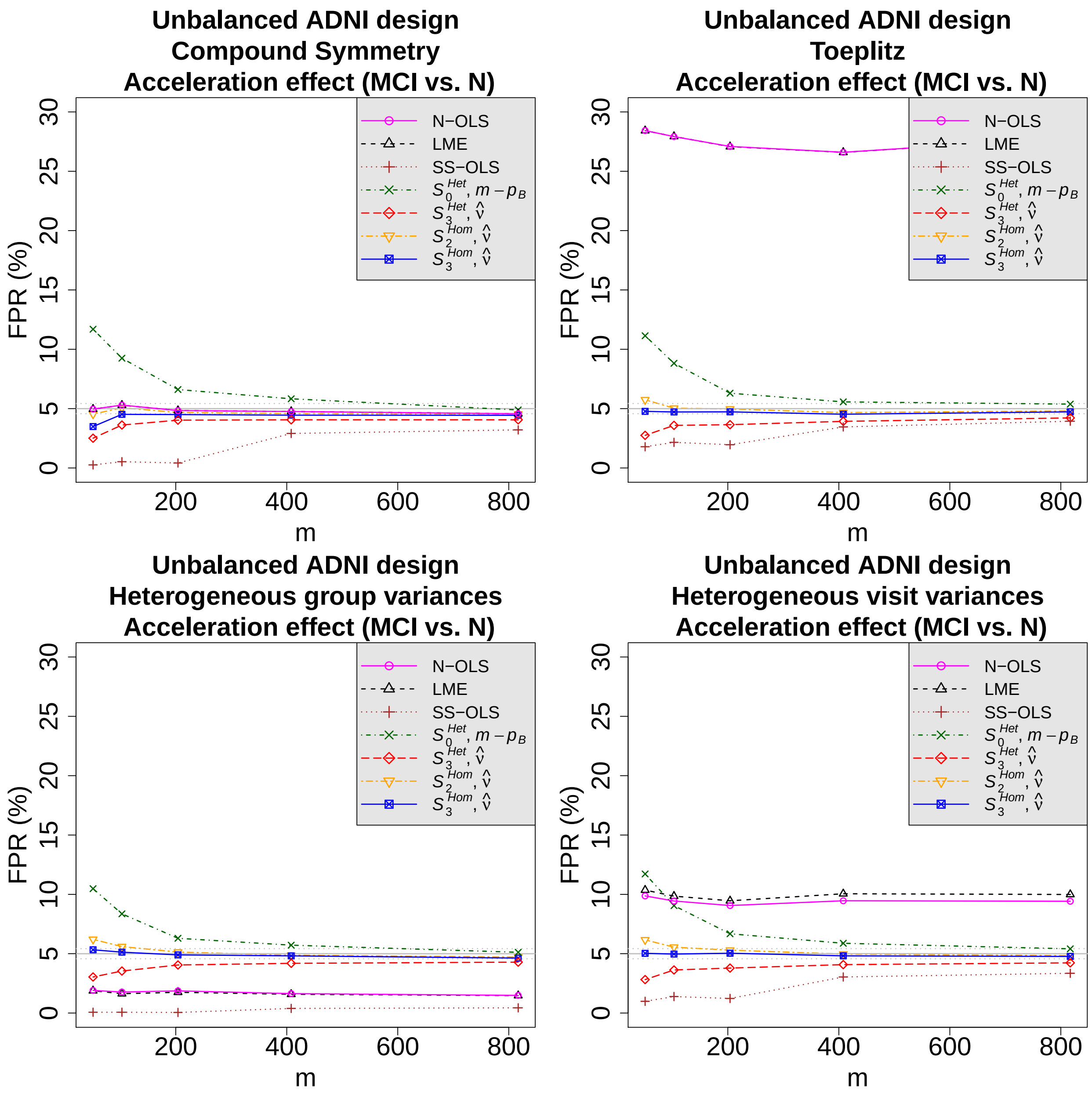

Supplementary Figure 49: FPR comparison on the acceleration effect difference between the MCI and Normal subjects with Compound Symmetry (top left, $\rho = 0.95$), Toeplitz (top right, $\psi = 0.2$ per year), heterogeneous group variances (bottom left, $\alpha_{\mathrm{N}} = 1$, $\alpha_{\mathrm{MCI}} = 2$ and $\alpha_{\mathrm{AD}} = 3$) and heterogeneous visit variances (bottom right, $\gamma = 2$ per year) for the unbalanced ADNI design; all results are based on an F-test at nominal level 5%; see Supplementary Figure 1 for a description of the SwE versions.

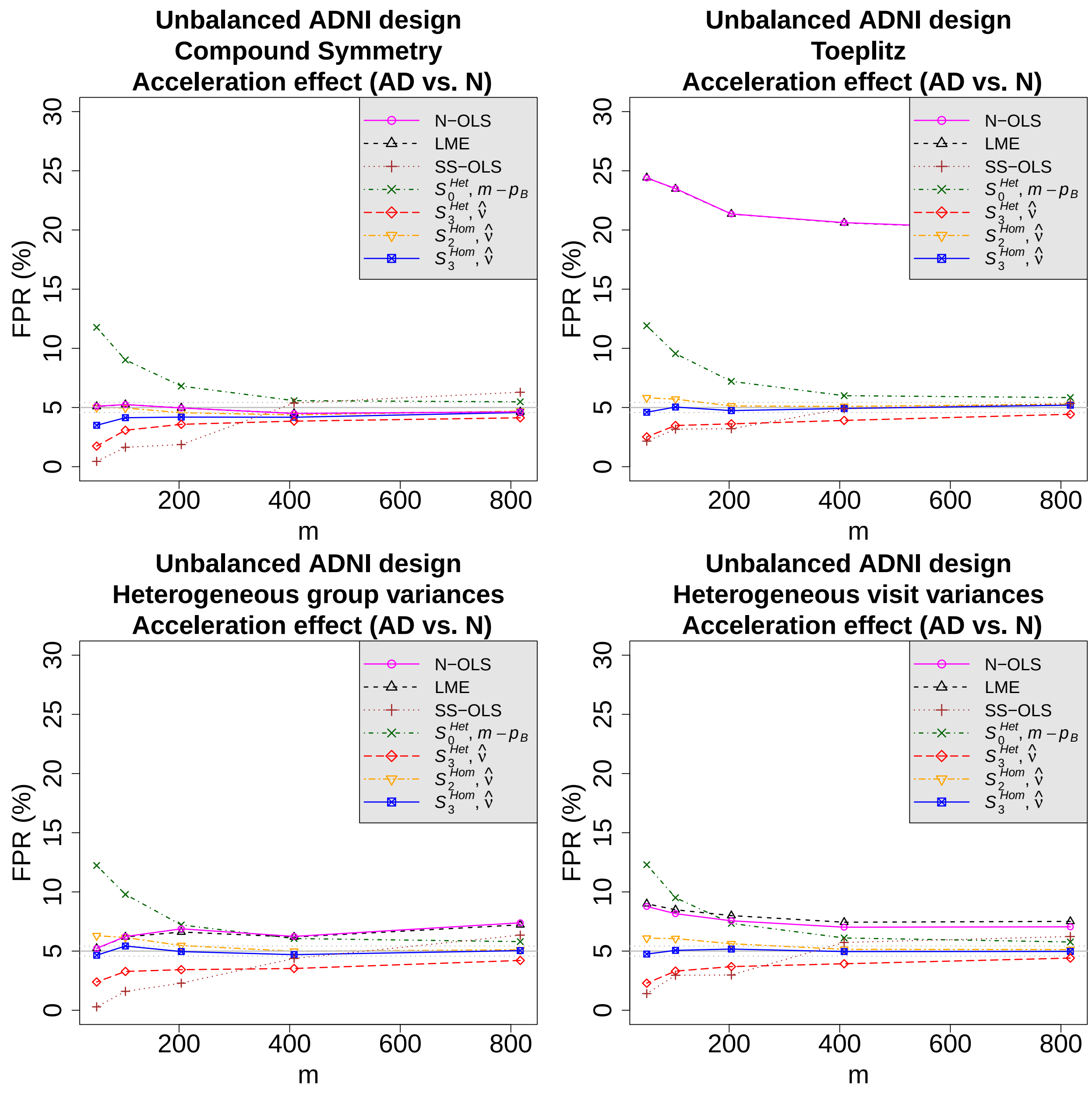

Supplementary Figure 50: FPR comparison on the acceleration effect difference between the AD and Normal subjects with Compound Symmetry (top left, $\rho = 0.95$), Toeplitz (top right, $\psi = 0.2$ per year), heterogeneous group variances (bottom left, $\alpha_{\mathrm{N}} = 1$, $\alpha_{\mathrm{MCI}} = 2$ and $\alpha_{\mathrm{AD}} = 3$) and heterogeneous visit variances (bottom right, $\gamma = 2$ per year) for the unbalanced ADNI design; all results are based on an F-test at nominal level 5%; see Supplementary Figure 1 for a description of the SwE versions.

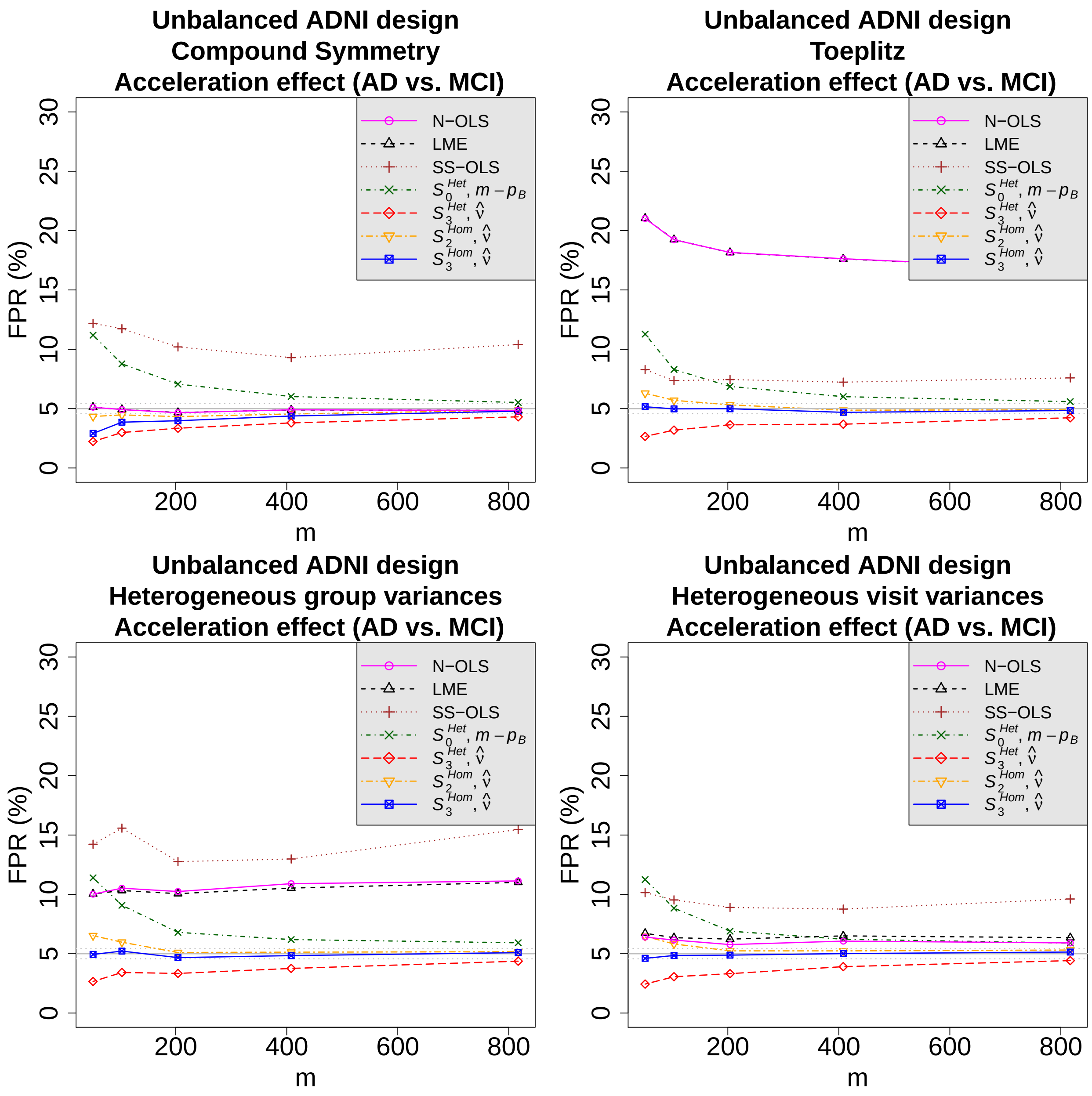

Supplementary Figure 51: FPR comparison on the acceleration effect difference between the AD and MCI subjects with Compound Symmetry (top left, $\rho = 0.95$), Toeplitz (top right, $\psi = 0.2$ per year), heterogeneous group variances (bottom left, $\alpha_{\mathrm{N}} = 1$, $\alpha_{\mathrm{MCI}} = 2$ and $\alpha_{\mathrm{AD}} = 3$) and heterogeneous visit variances (bottom right, $\gamma = 2$ per year) for the unbalanced ADNI design; all results are based on an F-test at nominal level 5%; see Supplementary Figure 1 for a description of the SwE versions.

Supplementary Appendix B: Additional results for the power analysis

In this Supplementary Appendix, we report additional results for the power analysis corresponding to the third set of simulations described in Subsection 2.5.3 of the main paper. Supplementary Figure 52 shows the results for a greater visit effect in AD relative to MCI subjects under the assumption of a Toeplitz covariance structure in the unbalanced ADNI design. For this case, only the SwE (S_3^{Hom}) seemed to be able to control accurately the FPR, with the N-OLS and random-intercept LME methods appearing highly liberal and the SS-OLS and the two richer LME methods appearing slightly liberal, making them invalid. An interesting observation about these results is that the SS-OLS method seemed to be slightly more or equally as powerful than the SwE method, contradicting the observation made in the CS case (see Subsection 3.2.2 of the main paper). Comparing the Monte Carlo estimates of the true variances of each method showed that the SS-OLS method is 1.2 times larger than the SwE method, indicating that the SS-OLS method should be less powerful than the SwE method, like in the CS case. Nevertheless, in this setting, the SS-OLS method actually underestimates the variance and in turn inflates the test statistic to such a degree that the SS-OLS is slightly more powerful.

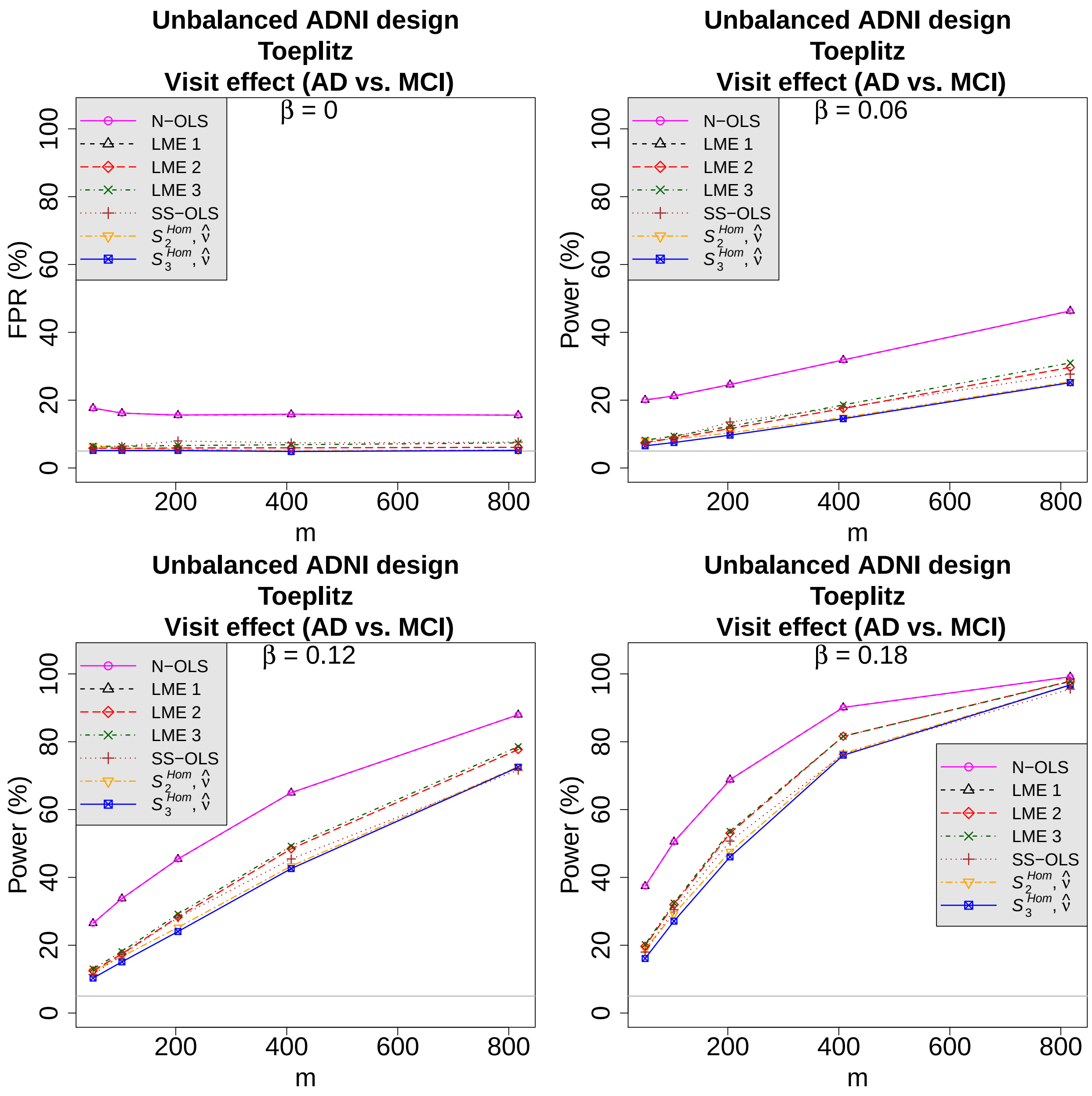

Supplementary Figure 52: Power with a Toeplitz covariance structure ($\psi = 0.2$ per year) for the unbalanced ADNI design, for varying effect sizes. The tested effect is the difference in the visit effect between AD and MCI groups. All results are based on an F-test at nominal level 5%. LME 1, LME 2 and LME 3 correspond to the LME model including a random intercept per subject, the LME model with a random intercept and a random effect of time per subject and the LME model with a random intercept, a random effect of time and a quadratic effect of time per subject, respectively. See Supplementary Figure 1 for a description of the SwE versions.

Supplementary Appendix C: LME convergence failure rates

In this Supplementary Appendix, we report the convergence failure rates obtained from the fourth set of simulations described in Subsection 2.5.4 of the main paper. Table 3, 4, 5 and 6 shows the failure rates obtained for the ADNI design, the balanced design with 3 visits, the balanced design with 5 visits and the balanced design with 8 visits, respectively.

ADNI design									
		LME 1		LME 2		LME 3		LME 4	
Cov. type	m	`lme4`	`nlme`	`lme4`	`nlme`	`lme4`	`nlme`	`lme4`	`nlme`
CS	51	0.00	0.00	0.00	35.00	0.00	75.10	n/a	0.00
	103	0.00	0.00	0.00	30.64	0.00	67.54	n/a	0.00
	204	0.00	0.00	0.00	29.05	0.00	61.93	n/a	0.00
	408	0.00	0.00	0.00	28.14	0.00	53.71	n/a	0.00
	817	0.00	0.00	0.00	27.78	0.00	47.14	n/a	0.00
Toeplitz	51	0.00	0.00	0.00	0.00	0.00	3.74	n/a	2.45
	103	0.00	0.00	0.00	0.00	0.00	0.22	n/a	0.19
	204	0.00	0.00	0.00	0.01	0.00	0.00	n/a	0.01
	408	0.00	0.00	0.00	0.00	0.00	0.00	n/a	0.00
	817	0.00	0.00	0.00	0.00	0.00	0.19	n/a	0.00
Het. groups	51	0.00	0.00	0.00	27.19	0.00	63.29	n/a	0.03
	103	0.00	0.00	0.00	19.49	0.00	52.82	n/a	0.05
	204	0.00	0.00	0.00	12.54	0.00	40.70	n/a	0.05
	408	0.00	0.00	0.00	6.79	0.00	23.79	n/a	0.02
	817	0.00	0.08	0.00	2.07	0.00	9.99	n/a	0.02
Het. visits	51	0.00	0.00	0.00	92.03	0.00	92.65	n/a	0.12
	103	0.00	0.00	0.00	96.91	0.00	93.63	n/a	0.05
	204	0.00	0.00	0.00	99.52	0.00	95.90	n/a	0.00
	408	0.00	0.00	0.00	99.95	0.00	97.57	n/a	0.00
	817	0.00	0.04	0.00	100.00	0.00	97.22	n/a	0.01

Table 3: Convergence failure rates in % for the ADNI design. LME 1, LME 2, LME 3 and LME 4 correspond to the LME with random intercept, the LME with random intercept and linear effect of time, the LME with random intercept, linear effect of time and quadratic effect of time, and the LME with random intercept and Toeplitz covariance structure for the error terms, respectively.

Balanced design with 3 visits									
		LME 1		LME 2		LME 3		LME 4	
Cov. type	m	`lme4`	`nlme`	`lme4`	`nlme`	`lme4`	`nlme`	`lme4`	`nlme`
CS	12	0.00	0.00	0.00	45.13	0.00	23.15	n/a	0.00
	25	0.00	0.00	0.00	41.68	0.00	23.18	n/a	0.00
	50	0.00	0.00	0.00	39.12	0.00	32.02	n/a	0.00
	100	0.00	0.00	0.00	36.78	0.00	39.69	n/a	0.00
	200	0.00	0.00	0.00	35.59	0.00	47.50	n/a	0.00
Toeplitz	12	0.00	0.00	0.00	6.84	0.00	11.39	n/a	0.11
	25	0.00	0.00	0.00	0.74	0.00	8.26	n/a	0.00
	50	0.00	0.00	0.00	0.01	0.00	6.49	n/a	0.00
	100	0.00	0.00	0.00	0.00	0.00	5.70	n/a	0.00
	200	0.00	0.00	0.00	0.00	0.00	4.75	n/a	0.01
Het. groups	12	0.00	0.00	0.00	51.05	0.00	34.09	n/a	1.87
	25	0.00	0.00	0.00	46.06	0.00	30.78	n/a	0.08
	50	0.00	0.00	0.00	41.85	0.00	32.77	n/a	0.06
	100	0.00	0.00	0.00	38.09	0.00	39.75	n/a	0.02
	200	0.00	0.08	0.00	33.77	0.00	43.32	n/a	0.01
Het. visits	12	0.00	0.00	0.00	68.02	0.00	32.51	n/a	1.43
	25	0.00	0.00	0.00	81.21	0.00	24.30	n/a	0.08
	50	0.00	0.00	0.00	92.98	0.00	17.02	n/a	0.08
	100	0.00	0.00	0.00	99.05	0.00	9.86	n/a	0.01
	200	0.00	0.04	0.00	99.97	0.00	6.10	n/a	0.02

Table 4: Convergence failure rates in % for the balanced design with 3 visits. See Table 3 for a description of the LME models.

Balanced design with 5 visits									
		LME 1		LME 2		LME 3		LME 4	
Cov. type	m	`lme4`	`nlme`	`lme4`	`nlme`	`lme4`	`nlme`	`lme4`	`nlme`
CS	12	0.00	0.00	0.00	45.41	0.00	83.89	n/a	0.03
	25	0.00	0.00	0.00	39.24	0.00	76.88	n/a	0.00
	50	0.00	0.00	0.00	33.84	0.00	69.60	n/a	0.00
	100	0.00	0.00	0.00	30.60	0.00	63.71	n/a	0.00
	200	0.00	0.00	0.00	28.04	0.00	56.18	n/a	0.00
Toeplitz	12	0.00	0.00	0.00	0.58	0.00	29.15	n/a	1.43
	25	0.00	0.00	0.00	0.01	0.00	9.98	n/a	0.12
	50	0.00	0.00	0.00	0.00	0.00	3.04	n/a	0.03
	100	0.00	0.00	0.00	0.00	0.00	1.06	n/a	0.02
	200	0.00	0.00	0.00	0.00	0.00	0.18	n/a	0.01
Het. groups	12	0.00	0.00	0.00	46.38	0.00	86.15	n/a	0.14
	25	0.00	0.00	0.00	41.72	0.00	81.27	n/a	0.03
	50	0.00	0.00	0.00	35.74	0.00	75.54	n/a	0.03
	100	0.00	0.00	0.00	29.70	0.00	65.31	n/a	0.00
	200	0.00	0.00	0.00	23.02	0.00	51.96	n/a	0.01
Het. visits	12	0.00	0.00	0.00	66.46	0.00	89.62	n/a	0.05
	25	0.00	0.00	0.00	81.30	0.00	92.07	n/a	0.01
	50	0.00	0.00	0.00	93.31	0.00	94.52	n/a	0.05
	100	0.00	0.00	0.00	98.86	0.00	97.37	n/a	0.02
	200	0.00	0.00	0.00	99.92	0.00	99.13	n/a	0.02

Table 5: Convergence failure rates in % for the balanced design with 5 visits. See Table 3 for a description of the LME models.

Balanced design with 8 visits									
		LME 1		LME 2		LME 3		LME 4	
Cov. type	m	`lme4`	`nlme`	`lme4`	`nlme`	`lme4`	`nlme`	`lme4`	`nlme`
CS	12	0.00	0.00	0.00	44.86	0.00	86.04	n/a	0.00
	25	0.00	0.00	0.00	38.17	0.00	80.28	n/a	0.00
	50	0.00	0.00	0.00	32.68	0.00	73.65	n/a	0.00
	100	0.00	0.00	0.00	27.64	0.00	64.29	n/a	0.00
	200	0.00	0.00	0.00	24.26	0.00	54.33	n/a	0.00
Toeplitz	12	0.00	0.00	0.00	0.09	0.00	11.55	n/a	6.64
	25	0.00	0.00	0.00	0.00	0.00	0.62	n/a	3.69
	50	0.00	0.00	0.00	0.00	0.00	0.01	n/a	0.72
	100	0.00	0.00	0.00	0.00	0.00	0.00	n/a	0.21
	200	0.00	0.00	0.00	0.12	0.00	0.00	n/a	0.01
Het. groups	12	0.00	0.00	0.00	43.17	0.00	83.93	n/a	0.02
	25	0.00	0.00	0.00	38.34	0.00	78.28	n/a	0.04
	50	0.00	0.00	0.00	31.67	0.00	70.00	n/a	0.02
	100	0.00	0.00	0.00	24.61	0.00	58.01	n/a	0.03
	200	0.00	0.00	0.00	17.47	0.00	43.38	n/a	0.05
Het. visits	12	0.00	0.00	0.00	63.94	0.00	81.47	n/a	0.07
	25	0.00	0.00	0.00	79.13	0.00	78.94	n/a	0.02
	50	0.00	0.00	0.00	92.50	0.00	73.92	n/a	0.01
	100	0.00	0.00	0.00	98.93	0.00	69.28	n/a	0.02
	200	0.00	0.00	0.00	99.90	0.00	67.58	n/a	0.02

Table 6: Convergence failure rates in % for the balanced design with 8 visits. See Table 3 for a description of the LME models.
